# Multiomics Profiling Reveals Distinct Immunosuppression and Metabolic Dysregulation in Aggressive Subtypes of Thyroid Cancer

**DOI:** 10.1016/j.mcpro.2026.101513

**Published:** 2026-01-19

**Authors:** Shanying Gui, Kate Huang, Jianling Qiang, Yunzhao Chen, Meifu Gan, Zhaochang Jiang, Jiazi Qian, Chenchen Yi, Yi Ding, Huihui Jiang, Fulong Zheng, Wanlin Lei, Lulu Jin, Xiaowei Zhang, Hezhi Fang, Maofeng Wang

**Affiliations:** 1 School of Basic Medical Sciences and Forensic Medicine, Hangzhou Medical College, Hangzhou, Zhejiang, China; 2 Department of Pathology, The First Affiliated Hospital of Wenzhou Medical University, Wenzhou, Zhejiang, China; 3 Department of Biomedical Sciences Laboratory, Affiliated Dongyang Hospital of Wenzhou Medical University, Dongyang, Zhejiang, China; 4 Cancer Center, Department of Pathology, Zhejiang Provincial People's Hospital (Affiliated People's Hospital), Hangzhou Medical College, Hangzhou, Zhejiang, China; 5 Department of Pathology, Taizhou Hospital of Zhejiang Province Affiliated to Wenzhou Medical University, Taizhou, Zhejiang, China; 6 Department of Pathology, Second Affiliated Hospital of Zhejiang University, School of Medicine, Hangzhou, Zhejiang, China; 7 Zhejiang Provincial Key Laboratory of Medical Genetics, College of Laboratory Medicine and Life Sciences, Wenzhou Medical University, Wenzhou, Zhejiang, China; 8 Department of Pathology, Affiliated Dongyang Hospital of Wenzhou Medical University, Dongyang, Zhejiang, China; 9 Department of Clinical Laboratory, National Cancer Center/National Clinical Research Center for Cancer/Cancer Hospital, Chinese Academy of Medical Sciences and Peking Union Medical College, Beijing, China

**Keywords:** ATC, FCGR2A, immune suppression, PDTC, thyroid cancer

## Abstract

Thyroid cancer comprises a heterogeneous group of malignancies with distinct clinical outcomes and molecular features, including papillary thyroid carcinoma (PTC), poorly differentiated thyroid carcinoma (PDTC), and anaplastic thyroid carcinoma (ATC). This study aimed to delineate the molecular and immune landscapes of these subtypes and identify potential biomarkers for the aggressive forms, ATC and PDTC. We assembled a well-annotated cohort of 120 formalin-fixed paraffin-embedded samples, including 35 ATC, 18 PDTC, 37 PTC cases, and 30 adjacent normal tissues (N) paired with PTC, collected over the past decade from multiple hospitals. To our knowledge, this represents the largest clinical ATC/PDTC cohort subjected to multiomics profiling and the first comprehensive proteomic analysis of these aggressive thyroid cancers. Using 4D–data-independent acquisition proteomics on 118 tumors (ATC 34, PDTC 18, PTC 36, and N 30), integrated with total RNA-seq on 69 samples (ATC 10, PDTC 5, PTC 31, and N 23), we revealed substantial molecular similarities between ATC and PDTC, both markedly distinct from PTC and adjacent normal tissues. ATC and PDTC exhibited significant enrichment in immune-related and metabolic pathways, with transcriptomic data indicating aggressive phenotypes and pronounced immunosuppression. Distinct immune landscapes of ATC and PDTC were revealed with neutrophil extracellular trap formation and M0 macrophage accumulation as key immunosuppressive mechanisms. Notably, Fc fragment of IgG receptor IIa (CD32) was identified as a promising biomarker for ATC, implicating a functional link between immune evasion and tumor aggressiveness. Our findings provide a comprehensive molecular and immunological characterization of thyroid cancer subtypes, offering novel insights into the pathogenesis of ATC and PDTC, and identifying potential targets for diagnosis and precision therapy.

## Highlights

• Largest multicentered ATC/PDTC cohort for multi-omics analysis.
• First 4D-DIA proteomic profiling of ATC/PDTC clinical samples.
• Aggressive molecular features and immunosuppressive TME in ATC/PDTC.
• FCGR2A (CD32) identified as a biomarker for ATC/PDTC.

## Graphical Abstract

**Figure:**
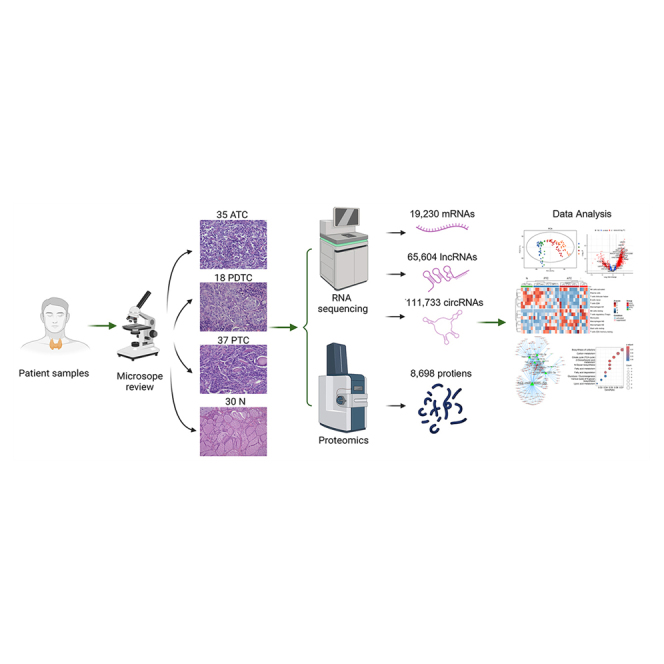

## In Brief

Using 4D-DIA proteomics integrated with total transcriptomics on 120 FFPE thyroid tumors, we chart molecular programs across PTC→PDTC→ATC. ATC shows heightened immune surveillance/evasion with suppressed fatty-acid/OXPHOS metabolism, whereas PDTC exhibits glycolysis-biased rewiring. We nominate the myeloid receptor FCGR2A as a protein biomarker of aggressive disease and validate key signals by IHC (n = 28), public bulk RNA sets, and single-cell data. These findings refine thyroid cancer taxonomy and suggest actionable immune–metabolic vulnerabilities.

Thyroid cancer is the most common endocrine malignancy, comprising papillary thyroid carcinoma (PTC), poorly differentiated thyroid carcinoma (PDTC), and anaplastic thyroid carcinoma (ATC) (1). These subtypes exhibit distinct clinical behaviors and outcomes (2). PTC, which accounts for approximately 85% of thyroid cancer cases, is well-differentiated and generally has a favorable prognosis, with a 10-year survival rate exceeding 90% (3). PDTC represents an intermediate subtype with partial loss of differentiation and worse survival rates, typically associated with increased metastatic potential and resistance to radioiodine therapy (4). ATC, although rare (accounting for <2% of cases), is the most aggressive form, with a median survival of less than 6 months due to rapid disease progression and resistance to nearly all conventional therapies (5). Due to the rareness of ATC and PDTC, there remains a critical need to elucidate the distinct molecular mechanisms underlying these aggressive subtypes in order to inform the development of effective targeted therapies (6).

At the molecular level, thyroid cancer progression is driven by cumulative genetic and epigenetic alterations (7). PTC is frequently associated with B-Raf proto-oncogene (BRAF^V600E^) mutations and PTC rearrangements, which are key drivers of tumor initiation and progression (8). PDTC and ATC exhibit more extensive genomic instability, with mutations in tumor protein p53, telomerase reverse transcriptase promoter, and PI3K and protein kinase B (AKT) pathway components contributing to their dedifferentiation and aggressiveness (9). These molecular characteristics not only define each subtype but also create therapeutic challenges, especially in PDTC and ATC, which often exhibit resistance to traditional treatments such as radioiodine therapy (10).

The tumor immune microenvironment (TIME) further differentiates thyroid cancer subtypes and influences their behavior (11, 12). ATC has a profoundly immunosuppressive TIME, characterized by high expression of immune checkpoint molecules such as programmed death-ligand 1 and programmed cell death protein 1, which facilitate immune evasion and tumor progression (13). This subtype is also associated with increased infiltration of tumor-associated macrophages and exhausted CD8+ T cells, further dampening the antitumor immune response (14). Conversely, PTC often shows an intermediate immune profile, with some degree of immune cell infiltration and immune checkpoint expression, particularly in tumors with aggressive mutations such as BRAF^V600E^ (15). Emerging research has highlighted novel immune checkpoint interactions, such as CD97/CD55, that contribute to immune escape and immune cell dysfunction in thyroid cancer (16).

Over the past decade, integrative multiomics has refined the molecular taxonomy of thyroid cancer across the PTC, PDTC, and ATC continuum. Large-cohort sequencing defined hallmark alterations and dedifferentiation programs in PDTC/ATC and benchmarked them against The Cancer Genome Atlas PTC, clarifying evolutionary links to high-grade transformation (7, 8, 9, 11, 17). Recent multiomics maps of the tumor microenvironment revealed macrophage-enriched, immunosuppressive states in ATC with features suggestive of relative immunotherapy sensitivity (8, 9). In parallel, studies implicate metabolic rewiring in undifferentiated disease, including activation of the mitochondrial one-carbon pathway and lipid metabolic dependencies that may create actionable vulnerabilities (11, 18). However, proteome-scale characterization in sizeable formalin-fixed paraffin-embedded (FFPE) cohorts remains scarce particularly for ATC and PDTC, leaving RNA–protein concordance and pathway-level effectors unresolved. Data-independent acquisition (DIA) now enables deep, reproducible proteomics from archival tissue, providing an opportunity to understand protein-level effectors.

In this study, we assembled a well-annotated cohort of 120 FFPE thyroid cancer samples from 35 ATC, 18 PDTC, and 37 PTC patients collected from four major hospitals. By employing 4D-DIA proteomics alongside transcriptomic profiling of long non-coding RNA (lncRNA), circular RNA (circRNA), and mRNA from tumor and adjacent normal tissues, we provided a comprehensive molecular characterization of these subtypes. To our knowledge, this represents the largest clinical cohort of ATC/PDTC patients subjected to multiomics profiling to date and constitutes the first in-depth proteomic investigation of these aggressive thyroid cancers. Our findings elucidate the distinct molecular pathways and immune landscapes of ATC and PDTC, highlight Fc fragment of IgG receptor IIa (FCGR2A, or CD32) as a potential biomarker, and offer novel insights into therapeutic vulnerabilities that may support more personalized and effective treatment strategies.

## Experimental Procedures

### Ethical Approval

Deidentified archival FFPE tissue samples were sourced from nine major hospitals, mainly from the Pathology Tissue Lab in Affiliated Dongyang Hospital of Wenzhou Medical University (Dongyang Hospital). The tissue collection was conducted in accordance with the Declaration of Helsinki (2013 revision) and was approved by the Institutional Review Board (approval number: 2024-YX-060). Each sample was handled in strict compliance with Health Insurance Portability and Accountability Act (1996) regulations, Pathology Department diagnostic requirements, and hospital by-laws. Given the retrospective nature of the study and the use of archived, anonymized specimens, the requirement for informed consent was waived by the Institutional Review Board.

### Experimental Design and Statistical Rationale

A total of 120 FFPE samples were collected from patients with histologically confirmed ATC, PDTC, PTC, or adjacent normal thyroid tissue, of which118 FFPE samples (34 ATC, 18 PDTC, 36 PTC, and 30 normal thyroid) passed proteomic quality control (QC) and were included in the final proteomics analysis. Due to the rareness of ATC and PDTC samples, the study design comprising relatively large sample sizes per group and technical replicate injections was chosen to provide sufficient statistical power and reproducibility for detecting proteomic differences between the thyroid cancer subtypes. A pooled sample was analyzed as the QC sample with 16 technical duplicates for every 10-sample run to assess analytical reproducibility. All samples were prepared and acquired in a single LC-MS/MS batch with consistent instrument settings and QC metrics to avoid significant batch effects. Fewer FFPE samples were included in whole transcriptomics (10 ATC, 5 PDTC, 31 PTC, and 23 N) due to RNA degradation. Validation was performed with bulk transcriptomes (GSE33630, GSE65144, GSE29265, GSE53072), single-cell transcriptomics (GSE232237), and protein-level immunohistochemistry (IHC) staining on 28 representative FFPE tissues (11 ATC, 7 PDTC, and 10 PTC). This cohort represents, to our knowledge, the largest proteomic study of ATC/PDTC to date, providing sufficient power to detect robust differences despite the rarity of these cancers.

### Patient Samples and Preparation

A total of 120 FFPE samples from patients with histologically confirmed ATC, PDTC, PTC, or adjacent normal thyroid tissue were included mainly from The First Affiliated Hospital of Wenzhou Medical University, and some supplemented from other eight hospitals (Supplemental Table S1). Samples with poor tissue quality, degraded RNA/protein, or prior neoadjuvant treatment were excluded. Both male and female patients were included in the study, and sex information was recorded as part of the clinical metadata for all samples. Demographic information, including age and weight at the time of diagnosis, was collected for all subjects and is summarized in Table 1. All FFPE samples were confirmed histologically using H&E staining and classified according to the 2022 World Health Organization Classification of Thyroid Tumors (19). Tissue sections (10 μm thick) were prepared using a HistoCore rotary microtome (Cat.149BIO000C1, Leica) and processed on Leica water bath systems for downstream analysis. Tissue sections were dewaxed, rehydrated through a graded ethanol series, stained with hematoxylin, and counterstained with eosin (Cat.C0105S, Beyotime). Sections were then dehydrated and mounted using neutral resin (Cat.G8590, Solarbio) for microscopic evaluation.

**Table 1:** Clinical and demographic characteristics of the study cohort

Characteristic	ATC	PDTC	PTC	*P* value
Gender(n)				
Male	17	7	15	0.34
Female	13	11	22	
Age	68.27 ± 2.03	65 ± 3.39	48.16 ± 2.22	<0.01
Tumor No.	1.13 ± 0.07	1.07 ± 0.07	1.60 ± 0.18	0.03
Maximum diameter of tumor (cm)	3.88 ± 0.34	4.58 ± 0.36	1.33 ± 0.16	<0.01
T (n)				
T1	3	1	29	<0.01
T2	8	4	3	
T3	13	10	2	
T4	2	1	2	
NA	9	2	1	
N (n)				
N0	5	6	6	0.22
N1	12	10	31	
NA	18	2	0	
M (n)				
M0	23	15	36	
M1	2	2	0	0.15
NA	10	1	1	
T3 (ng/ml)	0.77 ± 0.05	1.08 ± 0.11	1.12 ± 0.04	<0.01
T4 (ng/ml)	97.75 ± 6.56	93.87 ± 6.93	94.15 ± 2.75	0.12
FT3 (pg/ml)	2.65 ± 0.11	3.33 ± 0.16	3.40 ± 0.05	<0.01
FT4 (ng/dl)	0.99 ± 0.07	0.98 ± 0.09	0.94 ± 0.02	0.66
TSH (mIU/ml)	2.55 ± 0.65	1.60 ± 0.26	1.83 ± 0.19	0.27

Abbreviations: ATC, anaplastic thyroid carcinoma; NA, not available; PDTC, poorly differentiated thyroid carcinoma; PTC, papillary thyroid carcinoma.

Values are presented as mean ± SD or number (n). The tumor–normal mapping and per-sample details are provided in Supplemental Table S1 (“Paired Sample” sheet). Continuous variables are summarized as mean ± standard deviation (or median interquartile range, as appropriate) and compared across ATC, PDTC, and PTC using one-way ANOVA with Tukey’s *post hoc* test (or Kruskal–Wallis with Dunn’s test when normality was not met). Categorical variables are compared using χ^2^ or Fisher’s exact tests. Statistical significance was defined as *p* < 0.05.

TSH, thyroid-stimulating hormone.

### Proteomics Analysis

DIA-based proteomic profiling was carried out on FFPE thyroid tissues representing multiple disease groups. In total, 34 ATC samples, 18 PDTC samples, 36 PTC samples, and 30 N samples (Supplemental Table S1) passed QC and were successfully analyzed after QC. Tryptic peptides from each sample were subjected to nano-flow liquid chromatography–mass spectrometry (LC-MS/MS). Peptide separations were performed on a 15 cm × 75 μm inner diameter C18 analytical column (1.6 μm particles, IonOpticks) using a nanoLC system at a flow rate of 400 nl/min. Mobile phase A was 0.1% formic acid in water and mobile phase B was 0.1% formic acid in acetonitrile. The LC gradient was programmed as follows: 5% B at 0 min, linearly ramping to 22% B by 20 min, then to 37% B by 24 min, and to 80% B from 27 to 30 min (20). Eluting peptides were analyzed on a timsTOF Pro mass spectrometer (Bruker Daltonics) operated in DIA mode (data-independent acquisition Parallel Accumulation–Serial Fragmentation [diaPASEF]) using oToF Control v6. Source conditions were set to a capillary voltage of 1.4 kV, dry gas temperature of 180 °C, and dry gas flow of 3.0 l/min. Full-scan mass spectrometry (MS)1 spectra were acquired over an *m/z* range of 100 to 1700. For DIA MS2 acquisition, quadrupole isolation windows were scheduled as a function of TIMS scan time in the *m/z*–ion mobility space (diaPASEF). The diaPASEF MS2 windows covered 338.55 to 1188.55 *m/z* with 25 *m/z* window widths and no *m/z* overlap (adjacent boundary windows) across an ion mobility range of 0.6894 to 1.3363 (1/K0). The acquisition cycle comprised one MS1 survey scan followed by 11 diaPASEF scans (34 isolation windows in total), and the diaPASEF ramp time was 75 ms per TIMS frame, resulting in a total cycle time of ∼0.97 s (the full window scheme is provided in the Supplemental Table S8). Collision energy was ramped linearly as a function of ion mobility from 59 eV at 1/K0 = 1.6 V s/cm^2^ to 20 eV at 1/K0 = 0.7 V s/cm^2^ (20). An indexed retention time peptide standard mixture was spiked into each sample to facilitate retention time alignment across runs. All samples were analyzed in a single batch to maintain instrumental consistency. QC information was summarized in Supplemental Figures S1 and S2.

### Proteomics Data Processing

Raw mass spectrometric data were processed and analyzed using Spectronaut Pulsar (version 18.4, Biognosys). Peptide identification was performed in a library-free (directDIA) manner by searching against the UniProt human reference proteome (UniProtKB proteome ID UP000005640, downloaded February 1, 2023), which contains approximately 20,600 canonical protein entries (∼83,600 total sequences including isoforms). The database search parameters assumed trypsin/P specificity (cleavage after lysine or arginine) with up to two missed cleavages allowed. Carbamidomethylation of cysteine residues was set as a fixed modification, while methionine oxidation and protein N-terminal acetylation were included as variable modifications (20). Mass tolerances for precursor and fragment ions were set to dynamic values determined by Spectronaut’s internal mass calibration (*i*.*e*., adaptive tolerances optimized per run). Identification confidence was controlled at a false discovery rate (FDR) of 1% at both the peptide precursor and protein level (Q-value <0.01) (20). Protein identifications required at least one unique peptide per protein (Supplemental Figure S3). Quantification of peptides was conducted at the MS2 level by extracting fragment ion chromatogram areas, and a local normalization algorithm (as implemented in Spectronaut) was applied to correct for systematic signal variation across runs.

Further data filtering and imputation steps were applied to build a robust protein abundance matrix. Proteins were retained for quantitation only if they had quantitative values in at least two samples overall and had values present (nonmissing) in ≥50% of the samples in at least one of the four groups (ATC, PDTC, PTC, or N). For any protein passing these criteria, if a particular group still contained missing values but had ≥50% of its samples quantified for that protein, the missing values in that group were imputed by the mean of the observed values for that protein in the same group. Any remaining missing values (for proteins with sporadic detection) were then imputed with a small constant value, half of the minimum detected intensity value in the entire dataset, to represent values below the detection limit. Protein intensity values were log_2_-transformed to stabilize variance, and the median protein intensity in each sample was centered to correct for any loading differences. This processed and normalized proteomics dataset was used for all downstream analyses.

### Transcriptomics Analysis

Due to RNA degradation and occasionally sample contamination in some of the FFPE samples, 10 ATC, 5 PDTC, 31 PTC, and 23 N samples passed QC for further examination. Shanghai OE Biotech Company offered total transcriptome sequencing services, covering mRNA, lncRNA, and circRNA detection. Total RNA was isolated from FFPE samples using the RecoverAll Total Nucleic Acid Isolation Kit (Cat.AM1975, Life Technologies). Ribosomal RNA was depleted using the TruSeq Stranded Total RNA with Ribo-Zero Gold kit (Cat.RS-122-2301, Illumina). Libraries were constructed from fragmented RNA and sequenced on the Illumina NovaSeq 6000 platform. Clean reads were obtained by trimming adapters and filtering low-quality sequences using Fastq. Reads were aligned to the human genome with HISAT2 (RRID: SCR_015530), and gene expression levels were quantified as fragments per kilobase of transcript per million mapped reads. The log_2_-transformed and median centered transcriptomic dataset was used for all downstream analyses.

### Immune Cell Profiling

TIME characteristics were evaluated using transcriptomic data. Immune infiltration scores were calculated with ESTIMATE (RRID: SCR_026090) (21), and the abundance of immune cell subtypes was estimated using CIBERSORT (RRID:SCR_016955) (22). Neutrophil extracellular trap (NET) formation and the activity of immune-suppressive markers, such as regulatory T cells and M2 macrophages, were assessed through pathway analysis and protein quantification.

### Immunohistochemistry

IHC validation for protein markers were performed on 28 representative thyroid tumor tissues (11 ATC, 7 PDTC, and 10 PTC). Tissue sections (4 μm thick) were deparaffinized with xylene and rehydrated through graded ethanol. Antigen retrieval was performed using a citrate buffer solution (pH 6.0) at 95 °C for 15 min. Endogenous peroxidase activity was blocked with 3% hydrogen peroxide for 10 min at room temperature. Primary antibodies—including FCGR2A (RRID: AB_2246912, Cat.15625-1-AP, Proteintech, 1:600 dilution), ubiquitin-conjugating enzyme E2 C (UBE2C) (RRID: AB_11232220, Cat.66087-1-Ig, Proteintech, 1:500), and nucleotide-binding protein-like (NUBPL) (RRID: AB_2878402, Cat.17393-1-AP, Proteintech, 1:200)—were diluted in antibody dilution buffer and applied to tissue sections. The slides were incubated with the primary antibodies at 4 °C overnight. After washing with PBS, secondary horseradish peroxidase-conjugated antibodies (Cat.PV9000, ZSBiO) were applied, followed by incubation at room temperature for 1 h. Signal development was performed using DAB chromogen substrate (Cat.UM-9002, ZSBiO), and sections were counterstained with hematoxylin. Stained slides were mounted with neutral resin and imaged using a digital pathology scanner. The staining intensity and percentage of positive cells were evaluated in five random high-power fields (20x magnification) by two independent pathologists. An immunoreactivity score (0–3 scale: 0, negative; 1, weak; 2, moderate; 3, strong) was calculated by multiplying the intensity and percentage scores for each marker.

### Statistical Analysis

All analyses were performed in R v4.4.1 (two-sided tests unless stated). Continuous variables were assessed for normality (Shapiro–Wilk). Normally distributed data were compared using Student’s *t* test (two groups) or one-way ANOVA (≥3 groups) with Tukey’s *post hoc* tests. Non-normal data were compared using Wilcoxon rank-sum (two groups) or Kruskal–Wallis (≥3 groups) with Dunn’s *post hoc* tests; when multiple pairwise tests were performed, *p* values were adjusted by Benjamini–Hochberg (BH) FDR. Categorical variables were compared using χ^2^ tests or Fisher’s exact tests when expected counts were <5. Statistical significance was defined as *p* < 0.05 (or FDR <0.05 where multiple-testing correction is specified below). Effect sizes are reported as log_2_ fold change (log_2_FC) for omics data and as mean differences (with 95% confidence interval) where applicable.

#### Unsupervised Analyses

Principal component analysis (PCA) was performed on log_2_-transformed, median-normalized proteomic intensities and variance-stabilized RNA counts (centered and scaled). Hierarchical clustering used 1 − Pearson correlation as the distance metric with Ward.D2 linkage. Heat maps display row-wise Z-scores of features.

#### Proteomics (DIA) Differential Analysis

After preprocessing (local normalization in Spectronaut, log_2_ transformation, sample-wise median centering; see Methods), between-group differences in protein abundance were assessed with unpaired two-sided *t*-tests, except for PTC *versus* N-paired samples with paired two-sided *t*-tests. Proteins were called differentially expressed proteins (DEPs) with *p* < 0.05 and |log_2_FC| ≥ 1 (Supplemental Table S2). For multigroup displays (*e*.*g*., PTC *versus* PDTC *versus* ATC), ANOVA with Tukey’s *post hoc* tests was used for annotation; multiple comparisons were BH-adjusted. Correlations between protein abundance and clinical variables were performed using Spearman (nonparametric) tests with BH correction across features.

#### Transcriptomics (RNA-seq) Differential Analysis

Differentially expressed genes (DEGs) were identified using DESeq2 (RRID:SCR_015687) R package. DESeq2’s Wald test was applied with default size-factor normalization and dispersion estimation; *p* values were corrected by BH FDR, and genes with FDR (*q*) < 0.05 and |log_2_FC| ≥ 1 were considered significant (Supplemental Table S3). For visualization (volcano plots, heat maps), shrunken log_2_FC (when used) followed DESeq2 recommendations.

#### Pathway and Gene-Set Analyses

Overrepresentation analyses used the hypergeometric test with BH correction against Kyoto Encyclopedia of Genes and Genomes (KEGG) (RRID:SCR_012773). The background (universe) was defined per modality: all quantified proteins (proteomics) or all expressed genes passing RNA-seq filters (transcriptomics). Gene set enrichment analysis (GSEA, RRID:SCR_003199; fgsea implementation) was run with 10,000 permutations on preranked lists (*e*.*g*., by signed −log_10_ *p* or by log_2_FC where appropriate). Gene sets with FDR <0.25 were considered enriched per GSEA convention; FDR <0.05 indicates strong evidence.

#### Multiple Testing and Robustness

Unless otherwise specified, family-wise multiple testing within each analysis block (*e*.*g*., all proteins, all genes, all pathways) was addressed by BH FDR. All statistics were conducted in R with base stats, DESeq2 (23), fgsea (24), clusterProfiler (25), and rstatix (https://rpkgs.datanovia.com/rstatix/), and illustrated by ggplot2 (https://rpkgs.datanovia.com/ggpubr/) where applicable.

## Results

### Overview of Clinical Information and Sample Characterization

**Fig. 1:**
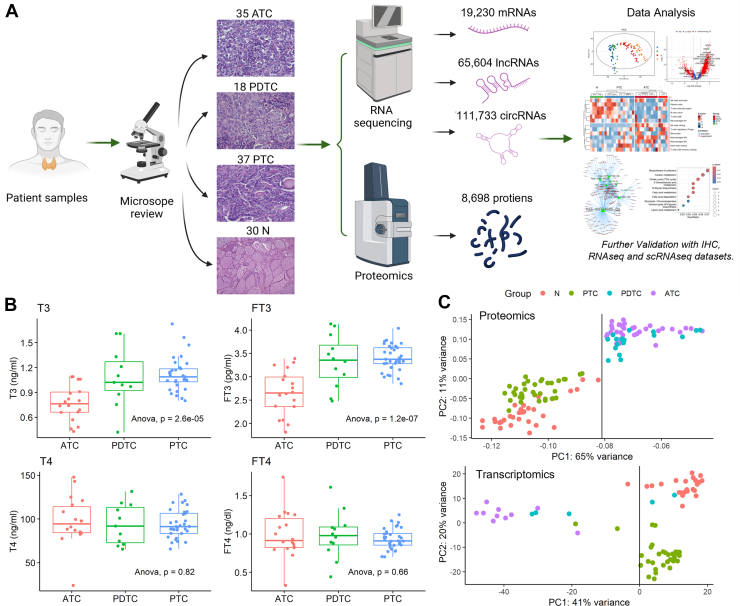
**Overview of study design and clinical data analysis.***A*, workflow illustrating the collection of ATC, PDTC, PTC, and N patient samples, followed by proteomic and transcriptomic analyses, integrated with publicly available datasets from The Cancer Genome Atlas and GTEx. Cohort and QC summary: proteomics samples include 34 ATC, 18 PDTC, 36 PTC, and 30 N; RNA-seq samples include 10 ATC, 5 PDTC, 31 PTC, and 23 N (See Supplemental Table S1 for the per-sample modality and QC status). *B*, measured levels of T3, FT3, T4, and FT4 across ATC, PDTC, and PTC samples compared with one-way ANOVA with Tukey’s *post hoc* test. *C*, principal component analysis (PCA) results from proteomics and transcriptomics datasets. ATC, anaplastic thyroid carcinoma; PDTC, poorly differentiated thyroid carcinoma; PTC, papillary thyroid carcinoma; QC, quality control.

We collected 120 FFPE samples from 85 thyroid cancer patients including ATC (n = 35), PDTC (n = 18), PTC (n = 37), and N (n = 30) tissues, with the N samples derived from the adjacent normal regions of the PTC samples (Fig. 1*A*, Supplemental Table S1). Clinical information for the cohort is summarized in Table 1. ATC/PDTC was found presenting older age, with larger tumors and more advanced stage than PTC, which were established phenotypic correlates of dedifferentiation in high-grade thyroid cancer consistent with prior large-cohort reports and recent reviews (7, 26, 27, 28). PCAs of all samples indicate no clear separation of clinical variates (Supplemental Figure S4). Levels of T3, free T3 (FT3), T4, free T4 (FT4), and thyroid-stimulating hormone (TSH) were measured for all patients. Notably, T3 and FT3 levels were significantly different among ATC, PDTC, and PTC patients (Fig. 1*B*), while no significant differences were observed across different tumor stages (T stages, Supplemental Figure S5, *A* and *B*). 4D-DIA proteomics identified a total of 8698 proteins, with a median of 4840 proteins among all samples (Supplemental Figure S5*C*). PCA of both proteomics and transcriptomics data consistently showed a cluster of ATC and PDTC distinct from PTC and N (Fig. 1*C*), highlighting the molecular similarities between ATC and PDTC and their separation from other groups. Based on sample availability and quality, proteomics was prioritized for subsequent bioinformatic analyses.

### Proteomic Analysis Reveals Distinct Subtypes

**Fig. 2:**
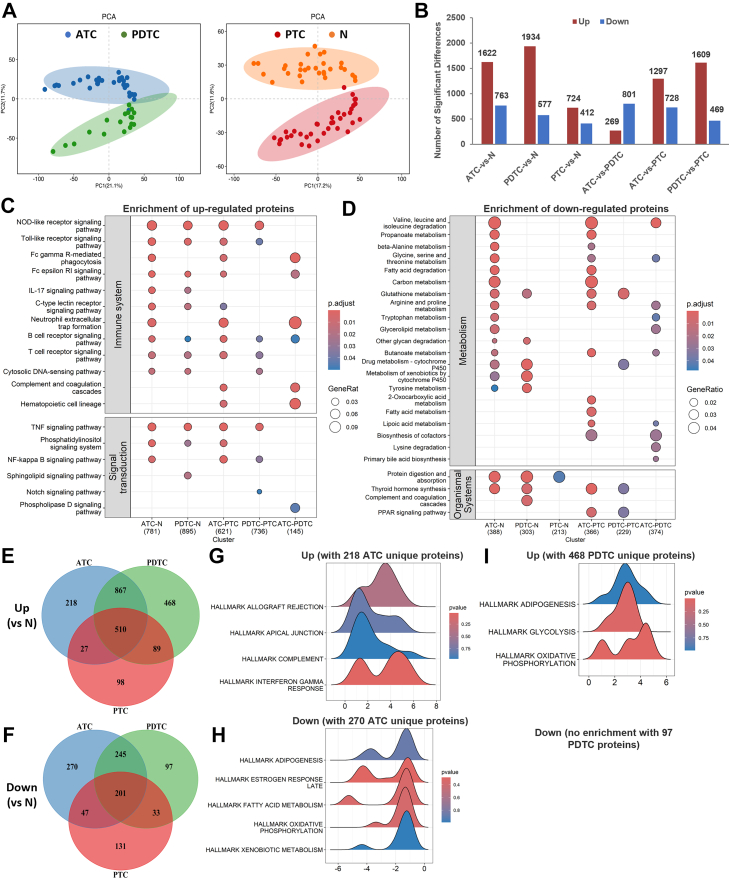
**Summary of 4D-DIA proteomics analysis.***A*, PCA results comparing ATC/PDTC and PTC/N groups. *B*, differentially expressed proteins (DEPs) identified from pairwise comparisons with |log_2_FC| ≥ 1 and *p* < 0.05 between groups. *C* and *D*, KEGG pathway enrichment analysis of upregulated (*C*) and downregulated (*D*) DEPs across groups. *E* and *F*, *Venn diagrams* displaying upregulated (*E*) and downregulated (*F*) DEPs compared to N. *G*–*I*, gene set enrichment analysis (GSEA) of DEPs uniquely upregulated (*G*) or downregulated (*H*) in ATC and uniquely upregulated in PDTC (*I*). The specific proteins contributing to each pathway shown in Figure 2 are provided in Supplemental Table S4. ATC, anaplastic thyroid carcinoma; DIA, data-independent acquisition; KEGG, Kyoto Encyclopedia of Genes and Genomes; log_2_FC, log_2_ fold change; PCA, principal component analysis; PDTC, poorly differentiated thyroid carcinoma; PTC, papillary thyroid carcinoma.

Although PCA of the proteomic data revealed significant similarities between ATC and PDTC, as well as between PTC and N, ATC formed a distinctly separate cluster from PDTC, and PTC was similarly distinct from N (Fig. 2*A*). ATC and PDTC samples exhibited significant proteomic differences, with 269 proteins upregulated and 801 downregulated in ATC samples (Fig. 2*B*). Over 1600 proteins were upregulated, and hundreds were downregulated in ATC and PDTC compared to N, while PTC showed fewer DEPs relative to N (Fig. 2*B*). Among group-wise comparisons, KEGG pathway enrichment of upregulated proteins in ATC and PDTC showed many immune-related pathways, such as B cell and T-cell receptor signaling, Fc gamma R-mediated phagocytosis, and neutrophil extracellular trap (NET) formation (Fig. 2*C*). Conversely, downregulated proteins in ATC and PDTC were primarily associated with amino acid and glucose metabolism pathways, including branched-chain amino acid degradation and propanoate metabolism (Fig. 2*D*).

Subtype-specific genes were sorted for GSEA to demonstrate distinct functional profiles for the aggressive ATC and PDTC subtypes (Fig. 2, *E* and *F*). ATC was characterized by upregulation of immune surveillance or evasion, such as allograft rejection and interferon-gamma responses (Fig. 2*G*), alongside downregulation of fatty acid metabolism and oxidative phosphorylation (Fig. 2*H*). PDTC, in contrast, showed a predominant enrichment of glycolysis and oxidative phosphorylation pathways (Fig. 2*I*), indicating a metabolic shift compared to ATC and PTC samples. KEGG enrichment based on pairwise comparisons also exhibited the same trends (Supplemental Figure S6). The specific proteins contributing to each pathway shown in Figure 2 are provided in Supplemental Table S4. Collectively, these results define a subtype-specific immune–metabolic axis in thyroid cancer.

### Molecular Classification of Thyroid Cancer Subtypes

**Fig. 3:**
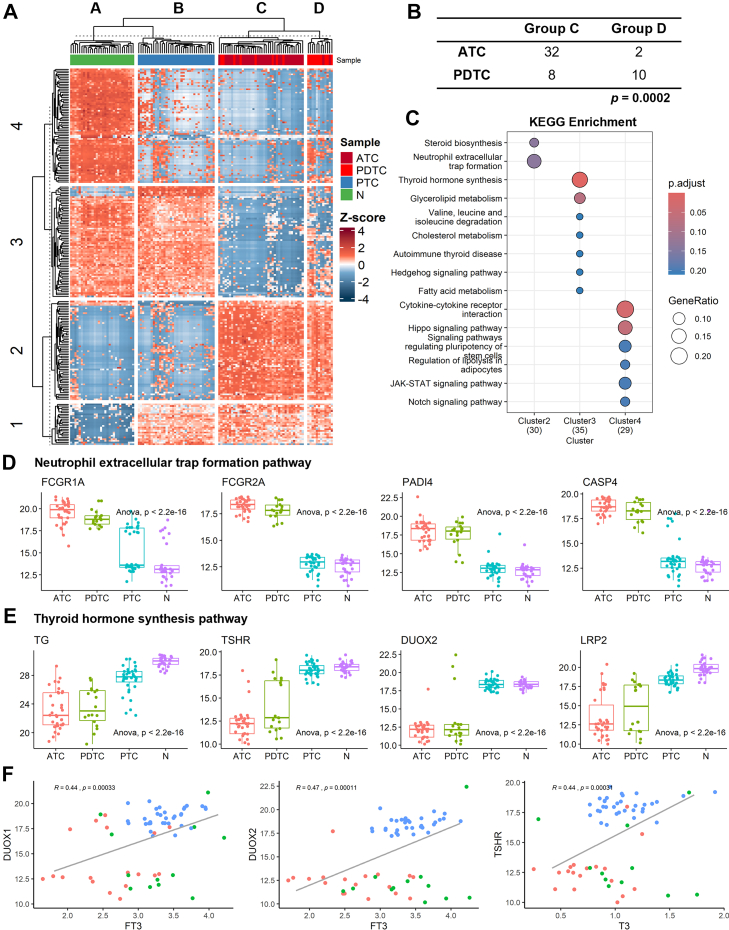
**Molecular classification of thyroid cancer subtypes based on DEPs.***A*, heat map of the *top* 200 DEPs with the highest |log_2_FC| and *p* < 0.05 between groups clustered using *k*-means. *B*, distribution of group C and D corresponding to ATC/PDTC, with chi-square test results confirming the clustering validity. *C*, KEGG enrichment of proteins clustered in the heat map. *D*–*E*, Distribution of key proteins in NET formation (*D*) and thyroid hormone synthesis (*E*) pathways across groups compared with one-way ANOVA with Tukey’s *post hoc* test. *F*, correlation between thyroid hormone synthesis-related proteins and thyroid hormone levels. ATC, anaplastic thyroid carcinoma; DEP, differentially expressed protein; KEGG, Kyoto Encyclopedia of Genes and Genomes; log_2_FC, log_2_ fold change; NET, neutrophil extracellular trap; PDTC, poorly differentiated thyroid carcinoma.

We selected the top 200 DEPs in ATC *versus* N contrast ranked by max |log_2_FC| with no forced balance of upregulation/downregulation, of which the minimum |log_2_FC| were used to filter top DEPs in PDTC/PTC *versus* N contrasts. All three contrasts merged and yielded a total of 221 Top DEPs (Supplemental Table S5). Hierarchical clustering effectively grouped the samples into four categories (group A–D, Fig. 3*A*), in which group A and B contained all N and PTC samples, respectively. Despite some overlap between PDTC and ATC samples within group C and D, chi-square tests confirmed that the *k*-means grouping largely preserved the pathological classifications and reflected molecular characteristics specific to each subtype (Fig. 3*B*).

Heat map analysis revealed four distinct molecular clusters: cluster 1 and cluster 4 showed high expression in tumor and normal samples, respectively, while cluster two was predominantly expressed in ATC-PDTC (group C/D) and cluster 3 in PTC-N (group A/B). Enrichment analysis of these clusters highlighted distinct pathways associated with tumor biology. Cluster two was enriched in NET formation, a hallmark pathway in ATC-PDTC, whereas cluster 3 was strongly enriched in the thyroid hormone synthesis pathway, predominantly active in PTC-N (Fig. 3*C*). Notably, the thyroid hormone synthesis pathway was significantly downregulated in ATC-PDTC, consistent with clinical findings of decreased T3 and FT3 levels in these subtypes (Fig. 1*B*). Further investigation into these pathways revealed that key proteins involved in NETs formation—including Fc gamma receptor Ia, FCGR2A, peptidyl arginine deiminase four (PADI4), and caspase four (CASP4) (29)—were significantly upregulated in ATC-PDTC (Fig. 3*D*), reflecting an immunosuppressive and inflammatory tumor microenvironment. Conversely, genes associated with thyroid hormone synthesis, such as thyroglobulin, TSH receptor, dual oxidase 1/2, and low-density lipoprotein receptor-related protein two (30)—were downregulated in ATC-PDTC (Fig. 3*E*), indicating the loss of thyroid-specific function during tumor dedifferentiation. Correlation analyses further confirmed that dual oxidase 1 and dual oxidase 2 were significantly associated with FT3 levels, while TSH receptor showed a strong correlation with T3 (Fig. 3*F*). These findings demonstrate that DEP-based molecular classification can effectively capture the distinct immune and metabolic characteristics of thyroid cancer subtypes. The upregulation of NET-related genes and the suppression of thyroid hormone synthesis pathways underscore the aggressive phenotype and metabolic disruption characteristic of ATC and PDTC.

### Transcriptomics Analysis Indicated Distinctly Aggressive Characteristics of ATC and PDTC

**Fig. 4:**
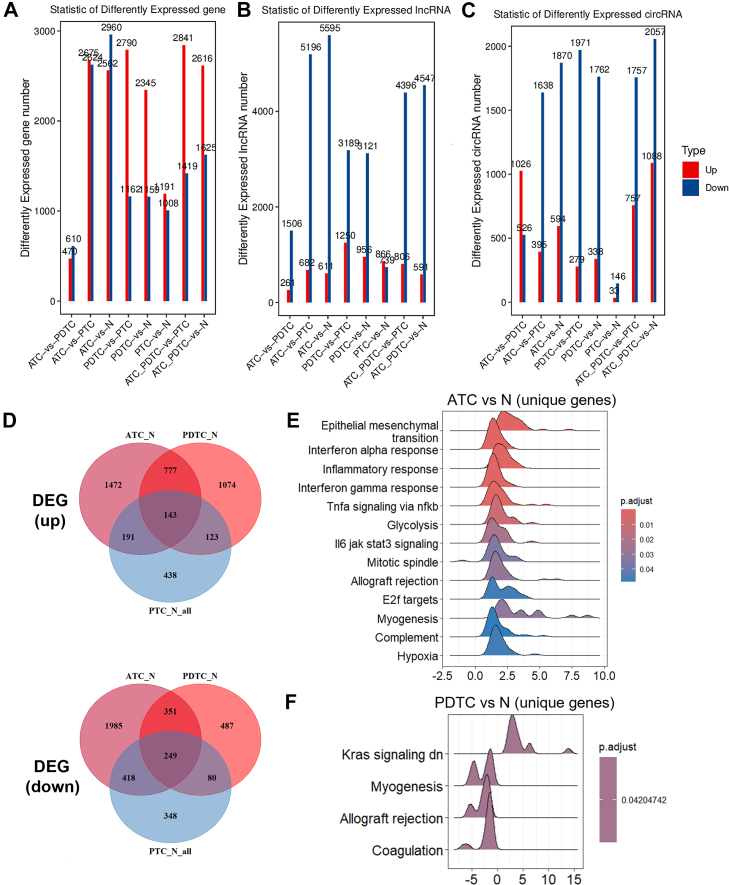
**Transcriptomic analyses of thyroid cancer subtypes.***A*–*C*, numbers summarized of differentially expressed mRNAs (*A*), long non-coding RNAs (*B*), and circRNAs (*C*) with |log_2_FC| ≥ 1 and FDR <0.05 between groups. *D*, Venn diagram of differentially expressed genes (DEGs) compared to N. *E* and *F*, GSEA of ATC-specific (*E*) and PDTC-specific (*F*) DEGs. ATC, anaplastic thyroid carcinoma; DEG, differentially expressed gene; FDR, false discovery rate; GSEA, gene set enrichment analysis; log_2_FC, log_2_ fold change; PDTC, poorly differentiated thyroid carcinoma.

RNA extraction from FFPE samples yielded fewer high-quality samples for ATC and PDTC, primarily due to RNA fragmentation during FFPE sample storage. PCA showed that ATC, PTC, and N samples formed distinct clusters, while PDTC samples were less defined due to limited numbers (Fig. 1*C*, Supplmenetal Figs. S7–S9). DEGs mirrored the proteomic findings, with ATC and PDTC exhibiting doubled DEGs compared to PTC or N (Fig. 4*A*). To ensure the reliability of our transcriptomic data, we compared it to publicly available ATC and PTC datasets, GSE33630 (31), GSE65144 (18), GSE29265, and GSE53072 (32), and confirmed that our results maintained high quality and consistency with a high percentage of overlapping DEGs between datasets (Supplemental Figure S10). Noncoding RNA profiles revealed extensive downregulation in ATC and PDTC, with over 90% of differentially expressed noncoding RNAs showing decreased expression, highlighting their loss of regulatory control in aggressive subtypes (Fig. 4, *B* and *C*). Sorting out unique DEGs in ATC and PDTC (Fig. 4*D*) demonstrated gene sets significantly enriched in tumor-related pathways, such as epithelial mesenchymal transition and inflammatory response for ATC, and allograft rejection for PDTC (Fig. 4*E*). Further noncoding RNA analyses also illustrated the similar findings, showing a significantly altered cell cycle and immune response in ATC and PDTC samples (Supplemental Figs. S11–S15). Therefore, proteomic and transcriptomic analyses revealed highly consistent results, highlighting the distinctly aggressive characteristics of ATC and PDTC, with significant alterations observed in immune-related pathways.

### Immunosuppression and NET Formation in ATC and PDTC Samples

**Fig. 5:**
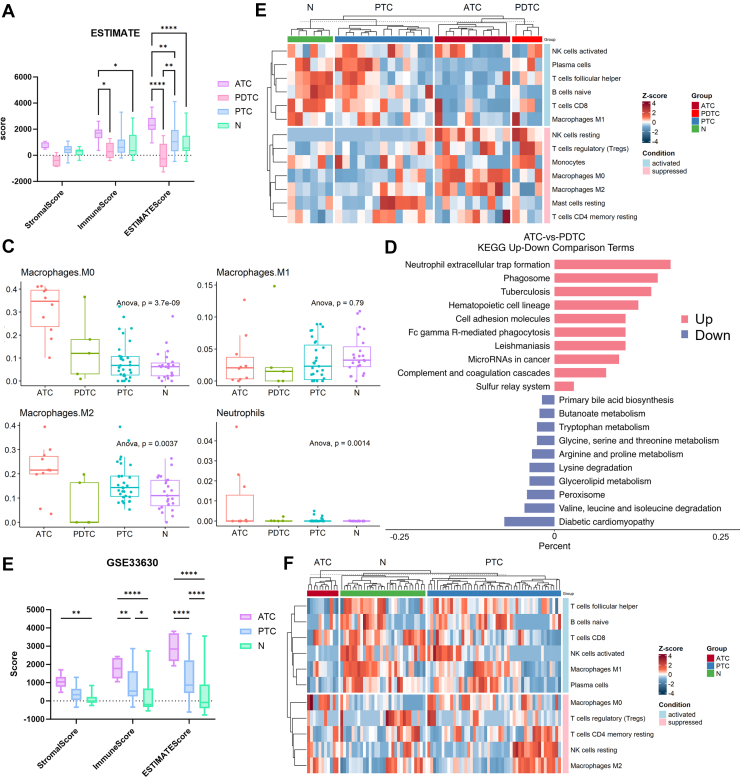
**Immune infiltration analysis from transcriptomic data.***A*, ESTIMATE analysis of immune and stromal components in transcriptomic samples. *B* and *C*, CIBERSORT-derived immune cell proportions across groups demonstrated in heat maps (*B*) and box plots (*C*) examined by one-way ANOVA with Tukey’s *post hoc* test. *D*, KEGG pathway enrichment analysis of DEGs in ATC *versus* PDTC. *E*, ESTIMATE analysis of immune and stromal scores in transcriptomic data from GSE33630. *F*, CIBERSORT immune cell deconvolution in GSE33630. ∗ *p* < 0.05; ∗∗ *p* < 0.01; and ∗∗∗∗ *p* < 0.0001. ATC, anaplastic thyroid carcinoma; DEG, differentially expressed gene; KEGG, Kyoto Encyclopedia of Genes and Genomes; PDTC, poorly differentiated thyroid carcinoma.

ATC and PDTC samples demonstrated strong immune pathway enrichment in the proteomics data. ESTIMATE analysis of the transcriptomics data revealed high immune and stromal scores for ATC, suggesting a complex TIME (Fig. 5*A*). Using CIBERSORT to deconvolute immune cell proportions demonstrated that immune activation markers, including CD8+ T cells and natural killer (NK) cells, were significantly reduced in ATC-PDTC compared to PTC-N, while immune-suppressive markers, such as resting NK cells, regulatory T cells, and M0 macrophages, were elevated in ATC (Fig. 5, *B* and *C*), further supporting its immunosuppressive TIME. NET formation, a hallmark of ATC, was prominently enriched even when compared to PDTC (Fig. 5*D*), with proteins such as PADI4 driving neutrophil activity and tumor progression. Additionally, the immune-suppressive characteristic was also confirmed on an independent dataset GSE33630 (Fig. 5, *E* and *F*). Due to the distinct aggressive and immunosuppressive features of ATC and PDTC, we combined ATC and PDTC samples comparing with PTC samples, the proteomic and transcriptomic results both indicated the same characteristics (Supplemental Tables S6 and S7).

### FCGR2A as a Key Biomarker of Aggressive Thyroid Cancer

**Fig. 6:**
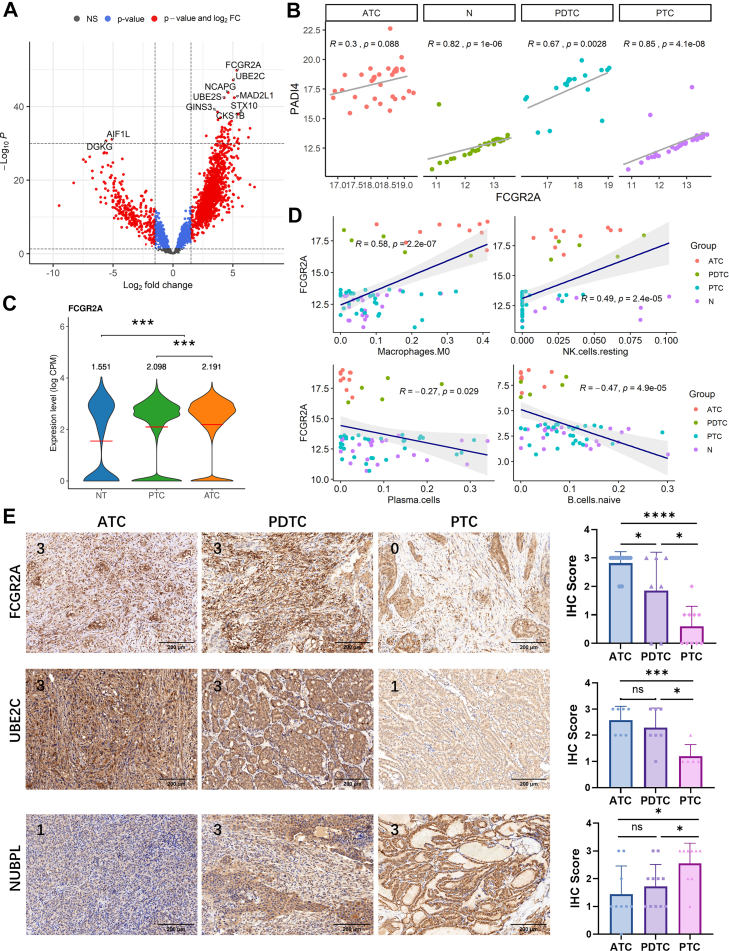
**Identification of potential biomarkers in ATC/PDTC.***A*, volcano plot of DEPs in ATC/PDTC *versus* PTC with |log_2_FC| ≥ 1 and *p* < 0.05. *B*, correlation analysis of FCGR2A with NET formation markers PADI4 across groups. *C*, single-cell RNA-seq data (GSE232237) showing FCGR2A expression predominantly in macrophages, with the highest expression in ATC. *D*, correlation analysis of FCGR2A expression with M0 macrophages, NK cells, and B cells examined by Spearman analysis. *E*, representative figures of IHC analysis validation of FCGR2A, UBE2C, and NUBPL expression in ATC (n = 11), PDTC (n = 7), and PTC (n = 10) tissues. The numbers at the *upper left corner* of each IHC image (0–3) indicate the immunoreactivity score for the representative case. The IHC scores were fully plotted in the bar plots assessed by pairwise student’s *t* tests. ns, not significant; ∗*p* < 0.05; ∗∗∗*p* < 0.001; and ∗∗∗∗*p* < 0.0001. ATC, anaplastic thyroid carcinoma; FCGR2A, Fc fragment of IgG receptor IIa; IHC, immunohistochemistry; log_2_FC, log_2_ fold change; NK, natural killer; PDTC, poorly differentiated thyroid carcinoma; PTC, papillary thyroid carcinoma; PADI4, peptidyl arginine deiminase 4; NUBPL, nucleotide-binding protein-like; UBE2C, ubiquitin-conjugating enzyme E2 C.

As the most significantly upregulated protein when comparing combined ATC and PDTC with PTC samples, FCGR2A emerged as a potential biomarker for ATC and PDTC, identified (Fig. 6*A*). Its expression was significantly correlated with two other NET formation-related proteins, PADI4 and CASP4 (33, 34), across all groups (Fig. 6*B*, Supplemental Figure S16). Single-cell RNA-seq analysis from the GSE232237 dataset (35) further revealed that FCGR2A was predominantly expressed in macrophages, with the highest expression observed in macrophages from ATC samples (Supplemental Figs. S17–S20, Fig. 6*C*). Correlation analyses showed that FCGR2A expression was positively correlated with NK cells and M0 macrophages, but negatively associated with B cells, highlighting its role in the immunosuppressive TIME (Fig. 6*D*). To validate its expression in clinical samples, IHC confirmed the significant upregulation of FCGR2A in ATC and PDTC tissues, reinforcing its role in shaping the suppressive immune landscape of ATC and PDTC subtypes (Fig. 6*E*). The other two protein signatures, UBE2C and NUBPL, also showed significantly upregulated and downregulated in ATC, respectively. IHC of these two proteins validated their expression difference in ATC and PDTC samples (Fig. 6*E*). UBE2C and NUBPL also maintain the same correlation trends with M0 macrophage, NK resting cells, and B cells (Supplemental Figure S21). Given its consistent association, FCGR2A represents a critical biomarker of thyroid cancer aggressiveness.

## Discussion

In this study, we performed proteomic and transcriptomic analyses on ATC, PDTC, PTC, and normal thyroid tissues, revealing significant molecular and immune landscape differences. ATC and PDTC shared common features, particularly immune suppression and metabolic dysregulation, distinguishing them from PTC and normal tissues. Enrichment analysis identified key pathways associated with immune evasion, notably NET formation, and downregulation of thyroid hormone synthesis, consistent with dedifferentiation in ATC and PDTC (36, 37).

A major finding of our study was the immunosuppressive tumor microenvironment in ATC and PDTC, characterized by upregulated NET formation, a pathway linked to immune suppression and tumor progression (38). Proteins such as PADI4 and CASP4, involved in NET formation, suggest a role for neutrophils in fostering a proinflammatory yet immune-suppressive microenvironment (33, 34). These findings suggest that neutrophils play a dual role in ATC progression: promoting an immunosuppressive environment while facilitating tumor growth and metastasis (39). NETs have been implicated in immune evasion and metastasis by trapping tumor cells and releasing immunosuppressive factors (40, 41, 42). In addition to NETs, tumor-associated macrophages showed distinct subtype-specific distributions (43). ATC exhibited a predominance of M0 and M2 macrophages, both contributing to immune suppression (44). M0 macrophages may transition into M2 under tumor influence (45), further promoting an immunosuppressive niche (46). Conversely, PTC and normal tissues had higher levels of immune-activating M1 macrophages (47), indicating a more active immune response (17). The depletion of CD8+ T cells and NK cells in ATC and PDTC further supports an immunosuppressive landscape (48, 49), reinforcing the role of tumor-associated macrophages and neutrophils in immune evasion (50, 51). These findings highlight the interplay between immune cell infiltration and tumor behavior in aggressive subtypes (52, 53).

Additionally, we identified FCGR2A (CD32) and UBE2C as key biomarkers of tumor aggression and immune suppression. FCGR2A, a receptor involved in antibody-dependent phagocytosis (54), was the most upregulated protein in ATC and PDTC and correlated with immune suppressive markers like PADI4 and CASP4. IHC confirmed its elevated expression in ATC tissues, supporting its role in immune regulation. Similarly, UBE2C, a regulator of cell cycle progression, was highly expressed in ATC and PDTC, reinforcing its role in tumor proliferation and immune evasion. Their consistent upregulation suggests their potential as biomarkers for distinguishing aggressive thyroid cancer subtypes and as targets for IHC-based diagnosis.

Our integrated proteomic and transcriptomic findings recapitulate and extend several established themes across the PTC, PDTC, and ATC continuum. We identified the close molecular proximity of ATC and PDTC with clear separation from PTC, which mirrors large-cohort genomic and/or transcriptomic studies defining high-grade transformation and dedifferentiation (7, 9). We also observed the prominently immunosuppressive TME marked by macrophage reprogramming and reduced cytotoxic compartments, which aligned with recent TME multiomics analyses showing that ATC, while more lethal, exhibits immune features suggestive of relative immunotherapy sensitivity (11). Pathway-level metabolic rewiring in ATC/PDTC (*e*.*g*., oxidative and lipid processes) was concordant with mitochondrial one-carbon pathway activation in undifferentiated disease (18, 35), as well as suppression of thyroid-hormone synthesis pathways and their correlation with circulating hormone measures which support functional dedifferentiation as a biological axis of aggressiveness. Notably, FCGR2A emerges here as a robust macrophage-linked protein marker, cohering with myeloid-enriched ATC TME reported previously, providing protein-level support from archival FFPE tissues to bridge RNA–protein gaps.

In summary, our study provides novel insights into the molecular and immune landscapes of thyroid cancer subtypes, emphasizing the immunosuppressive features of ATC and PDTC. The identification of NET formation and M0 macrophage elevation highlights the importance of immune suppression in tumor progression. Additionally, FCGR2A and UBE2C emerge as promising biomarkers for aggressive thyroid cancers, with potential diagnostic and therapeutic applications. These findings underscore the need for further investigation into immune modulation and metabolic pathways to develop targeted treatments for aggressive thyroid cancers.

## Data Availability

The mass spectrometry proteomics data (RAW and search files) have been deposited to the ProteomeXchange Consortium (https://proteomecentral.proteomexchange.org) *via* the iProX partner repository (55, 56) with the dataset identifier PXD068266. The annotated counts data and fragment per kilobase of transcript per million mapped reads results were uploaded to BioStudies under accession S-BSST1925 including separate files for mRNA, lncRNA, and circRNA. Full DEP and DEG results were provided in the Supplemental Tables S2, S3, S6 and S7. Public datasets used for orthogonal validation are available from GEO: GSE33630, GSE65144, GSE29265, GSE53072 (bulk transcriptomes), and GSE232237 (single-cell transcriptomes). Any additional information, including analysis scripts used to generate figures/tables, will be provided by the corresponding author upon reasonable request.

## Supplemental Data

This article contains supplemental data.

## Conflict of Interest

The authors declare no competing interests.
