# Supplementary material for: Multiomics Profiling Reveals Distinct Immunosuppression and Metabolic Dysregulation in Aggressive Subtypes of Thyroid Cancer
Source: Mol Cell Proteomics. 2026 Jan 19;25(3):101513. doi: 10.1016/j.mcpro.2026.101513 (PMC12964017; doi:10.1016/j.mcpro.2026.101513)
Supplement: Supplemental Materials [file mmc9.docx]

**Supplementary Tables**

**Table S1.** Sample information. “Omics Samples” sheet includes all sample ID with the corresponding source hospital, sample ID in transcriptomics and proteomics, whether passed QC, and the thyroid cancer type. “Paired Sample” sheet includes the pair information of PTC sample and its adjacent normal sample ID.

**Table S2.** Differentially expressed protein lists of pairwise comparisons. Each sheet contains DEPs of one comparison (e.g. ATC-vs-N, ATC-vs-PDTC, ATC-vs-PTC, PDTC-vs-N, PDTC-vs-PTC, and PTC-vs-N) and the full table of normalized and annotated protein information.

**Table S3.** Differentially expressed gene lists of pairwise comparisons, with foldchange (FC), *p*-value, and change direction (if significant) of each comparison. Full gene list was included without filtering.

**Table S4.** Gene set enrichment based on KEGG or GSEA with full information for each listed pathway and protein ID listed in Figure 2.

**Table S5.** The top 200 proteins from combined thyroid cancer types of heatmap in Figure 3A.

**Table S6.** Differentially expressed protein lists of combined ATC and PDTC samples compared with PTC samples, with FC threshold of 2 and q-value threshold of 0.05.

**Table S7.** Differentially expressed gene lists of combined ATC and PDTC samples compared with PTC samples, with FC threshold of 2 and q-value threshold of 0.05.

**Supplementary Figures**

**Figure S1.** LC–MS/MS quality control and run-order assessment. (A) Peak capacity: Distribution of chromatographic peak capacity per run; all samples > 200, indicating robust separations. (B) Median full width at half maximum (FWHM) per run; median < 0.2 min, consistent with stable peak shapes.

**Figure S2.** (A) Mix-QC identification overlap. Venn diagram of 16 pooled Mix-QC injections, showing a large shared set of identified proteins (intersection > 75%), indicative of stable identifications over time. (B) Mix-QC reproducibility. Pairwise Pearson correlation heatmap of protein intensities across the 16 Mix-QC runs (log₂ scale), demonstrating high between-run concordance.

**Figure S3.** Annotated spectra of single-peptide protein FCGR2A.

**Figure S4.** PCA analysis of proteomics of all samples colored by age (A) and tumor size (B).

**Figure S5.** Clinical characteristics related to thyroid hormone levels and tumor staging. (A) TSH levels in ATC, PDTC, and PTC. (B) Thyroid hormone levels across different T stages. (C) Quality control and Number of proteins identified per sample.

**Figure S6.** KEGG pathway enrichment of DEPs from pairwise comparisons between groups of 4D-DIA proteomic analysis.

**Figure S7.** Transcriptomic analysis quality control and functional enrichment. (A) PCA analysis of transcriptomic samples. (B) Volcano plot of DEGs comparing ATC/PDTC vs PTC.

**Figure S8.** KEGG enrichment of DEGs up- (A) and down-(B) regulated in combined ATC-PDTC samples vs PTC samples.

**Figure S9.** KEGG enrichment of DEGs up- (A) and down-(B) regulated in combined PTC samples vs N samples.

**Figure S10.** Venn diagram of validated DEGs from published transcriptomic datasets comparing ATC or PTC samples to N.

**Figure S11.** KEGG enrichment of lncRNA-associated target genes in ATC-PDTC vs N (A) and ATC-PDTC vs PTC (B).

**Figure S12.** Network analysis of lncRNA-miRNA interactions in pairwise comparisons including ATC-PDTC vs N (A), ATC-PDTC vs PTC (B), and ATC vs PDTC (C).

**Figure S13.** Interaction network of lncRNAs and transcription factors between ATC vs PDTC (A) and ATC-PDTC vs N (B).

**Figure S14.** KEGG enrichment of circRNA-associated target genes in ATC-PDTC vs N (A) and ATC-PDTC vs PTC (B).

**Figure S15.** Network analysis of circRNA-miRNA interactions in ATC-PDTC vs PTC (A) and ATC vs PDTC (B).

**Figure S16.** Correlation analysis between FCGR2A and CASP4.

**Figure S17.** Single-cell validation of FCGR2A expression across cell types and thyroid cancer subtypes of all samples (GSE232237). (A) Cell-type annotation (UMAP) embedding of the published scRNA-seq dataset (GSE232237) with major lineages labeled using BlueprintEncode reference signatures. Clusters include macrophages/monocytes, T/NK cells, B cells/DCs, epithelial/tumor cells, endothelial cells, fibroblasts, and keratinocytes. (B) Feature plot of FCGR2A. UMAP colored by FCGR2A expression (log-normalized counts; logCPM) with signals concentrated in myeloid compartments (macrophages), consistent with a myeloid-linked marker. (C) Groupwise expression of FCGR2A expression (logCPM) in normal thyroid (NT), PTC, and ATC cells. Mean values are shown above each group (NT 0.016; PTC 0.073; ATC 0.088). Statistical comparisons used Kruskal–Wallis with Dunn’s pairwise tests (BH correction); *** indicates q < 0.001 for ATC > PTC > NT.

**Figure S18.** Single-cell validation of FCGR2A expression in ATC subtypes of GSE232237. (A) Cell-type annotation of ATC samples in GSE232237 with major lineages labeled using BlueprintEncode reference signatures. (B) Feature plot of FCGR2A. (C) Quantitated FCGR2A expression (logCPM) in ATC cells.

**Figure S19.** Single-cell validation of FCGR2A expression in PTC subtypes of GSE232237. (A) Cell-type annotation of PTC samples in GSE232237 with major lineages labeled using BlueprintEncode reference signatures. (B) Feature plot of FCGR2A. (C) Quantitated FCGR2A expression (logCPM) in PTC cells.

**Figure S20.** Single-cell validation of FCGR2A expression in normal thyroid (NT) of GSE232237. (A) Cell-type annotation of NT samples in GSE232237 with major lineages labeled using BlueprintEncode reference signatures. (B) Feature plot of FCGR2A. (C) Quantitated FCGR2A expression (logCPM) in NT cells.

**Figure S21.** Correlation analysis between protein biomarkers UBE2C and NUBPL with immune infiltration.

**Figure S1.**

**
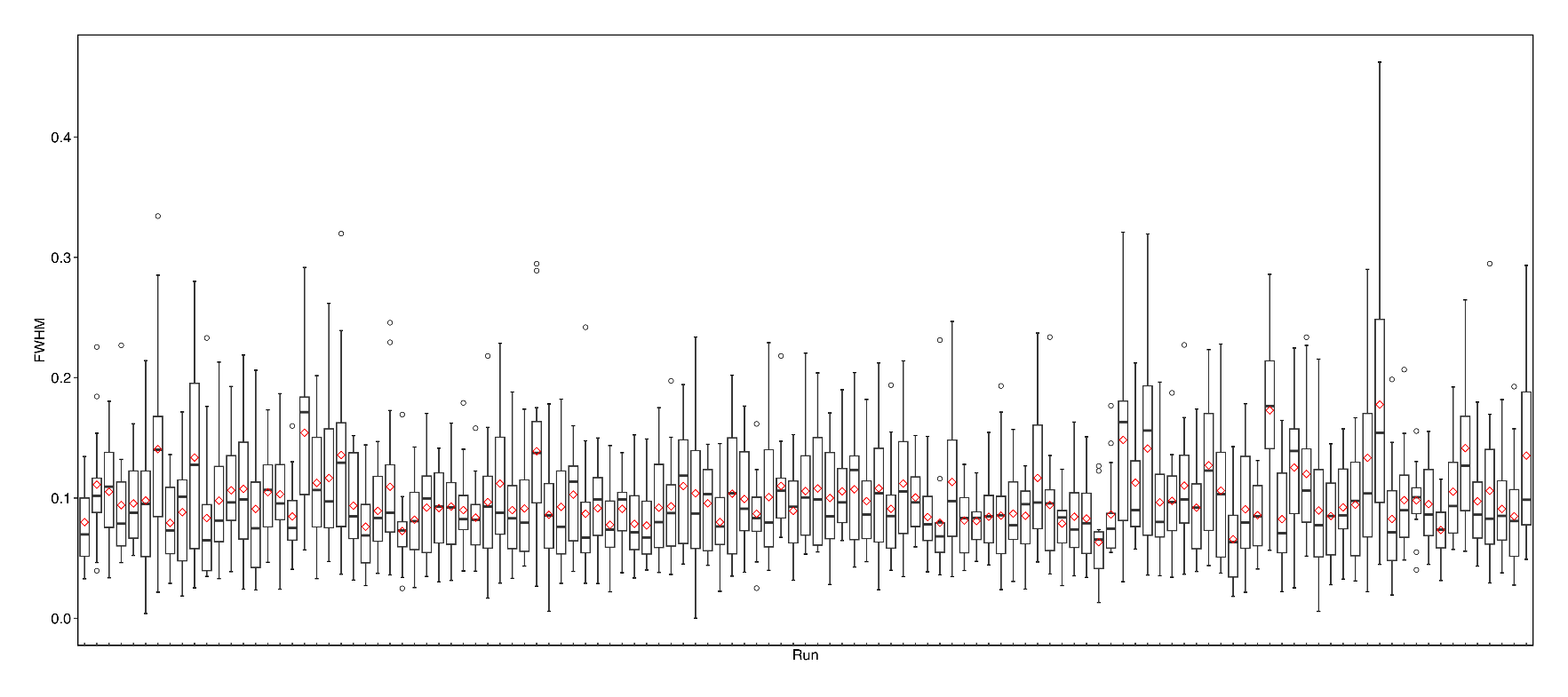
A**

**
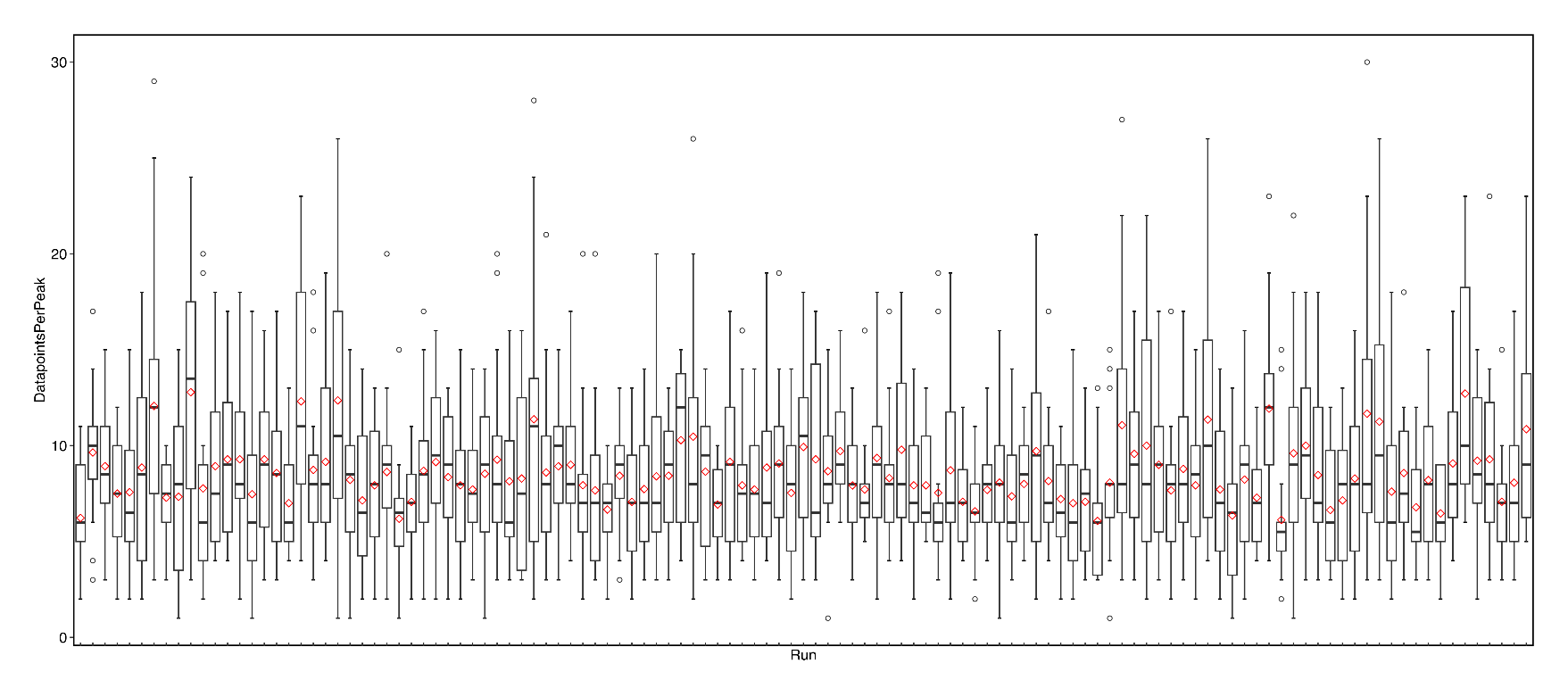
B**

**Figure S2A.**

**
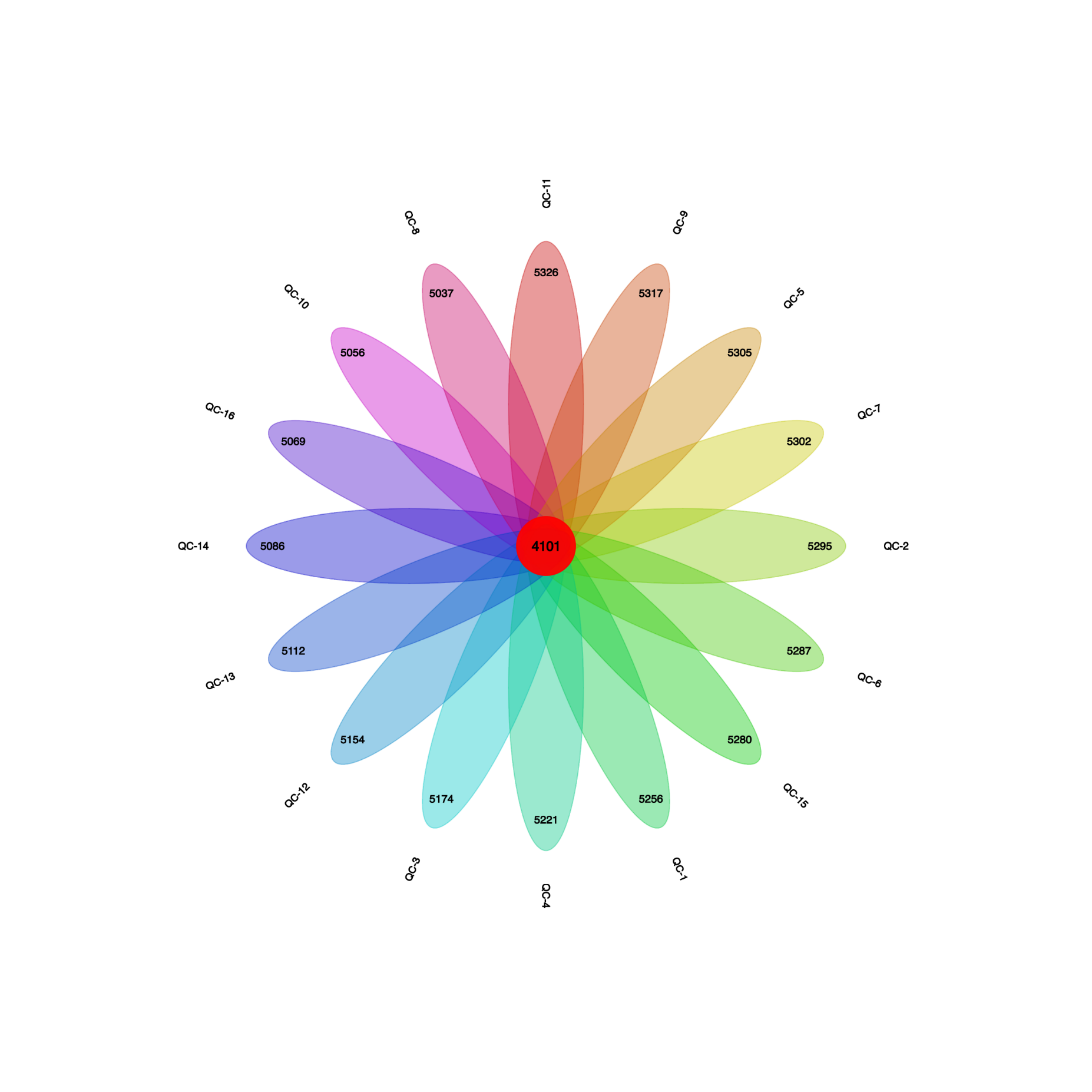
**

**Figure S2B.**

**
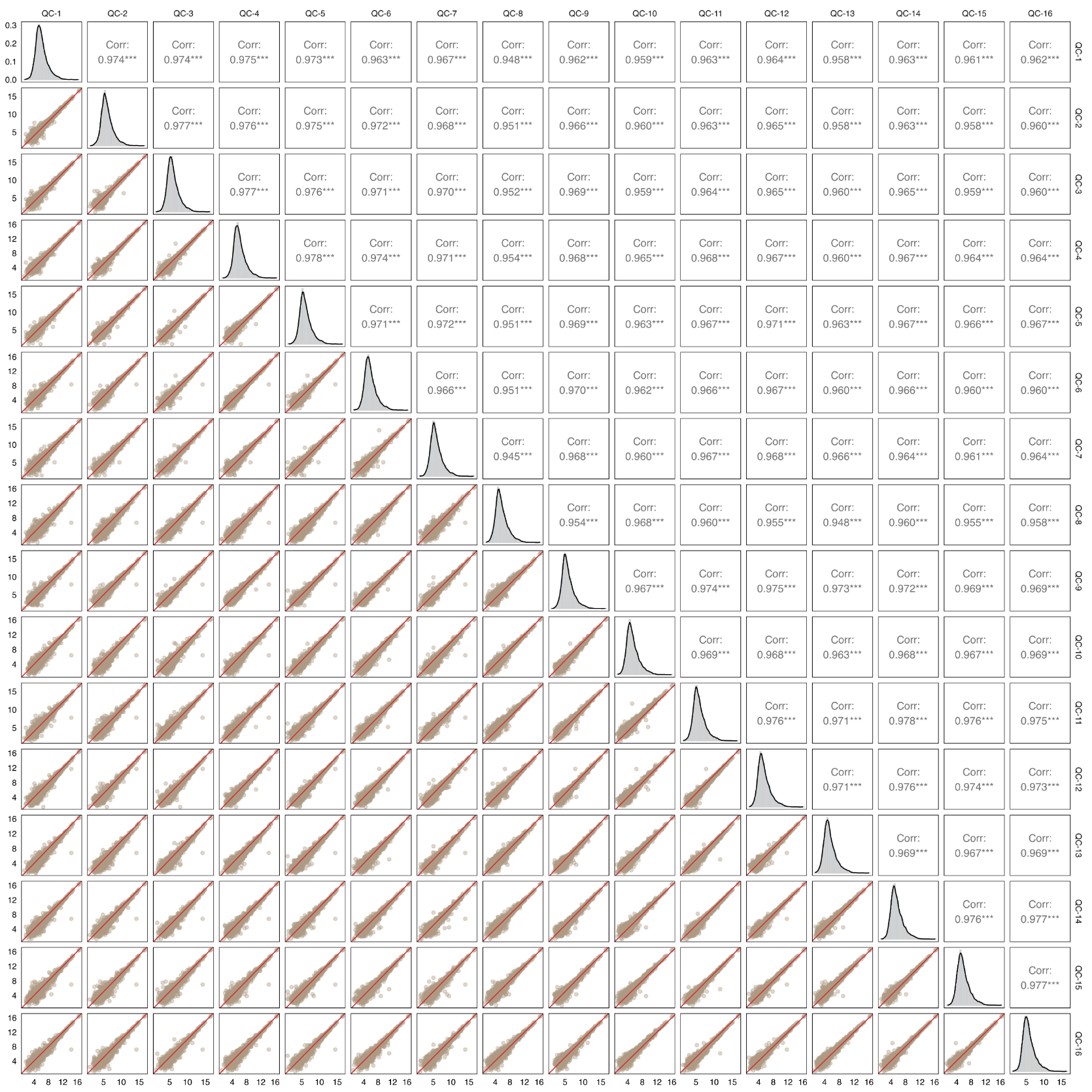
**

**Figure S3.**

**
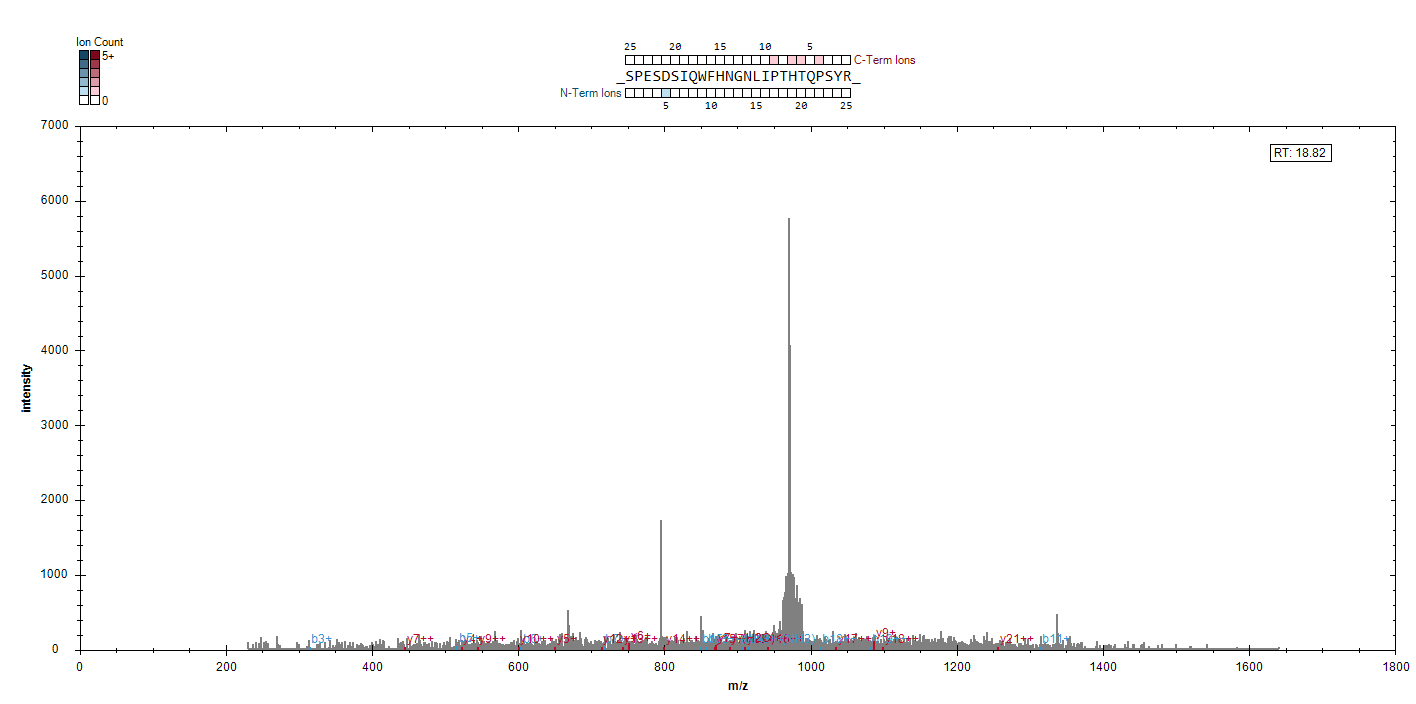
**

**Figure S4.**

**
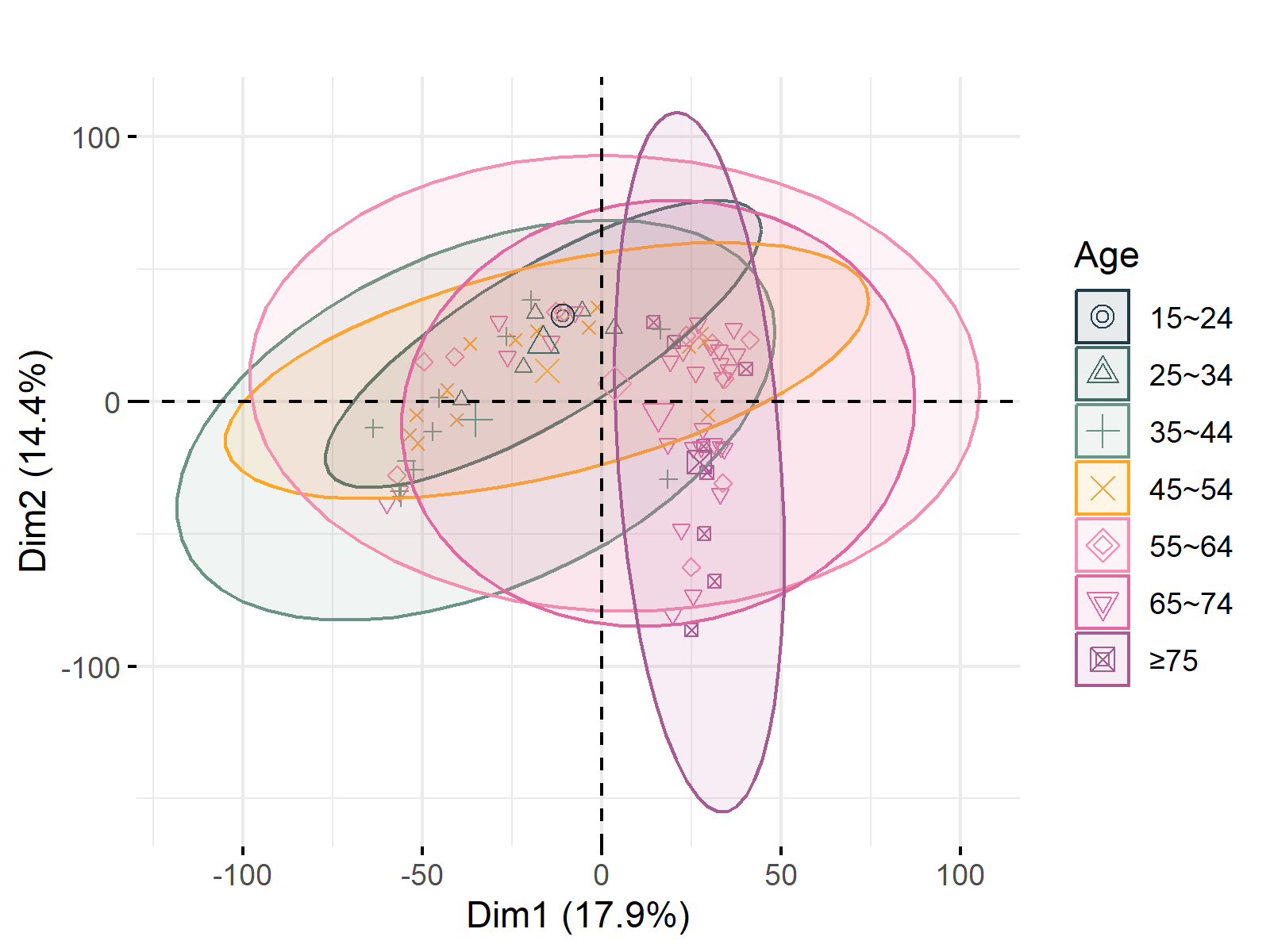
A.**

**
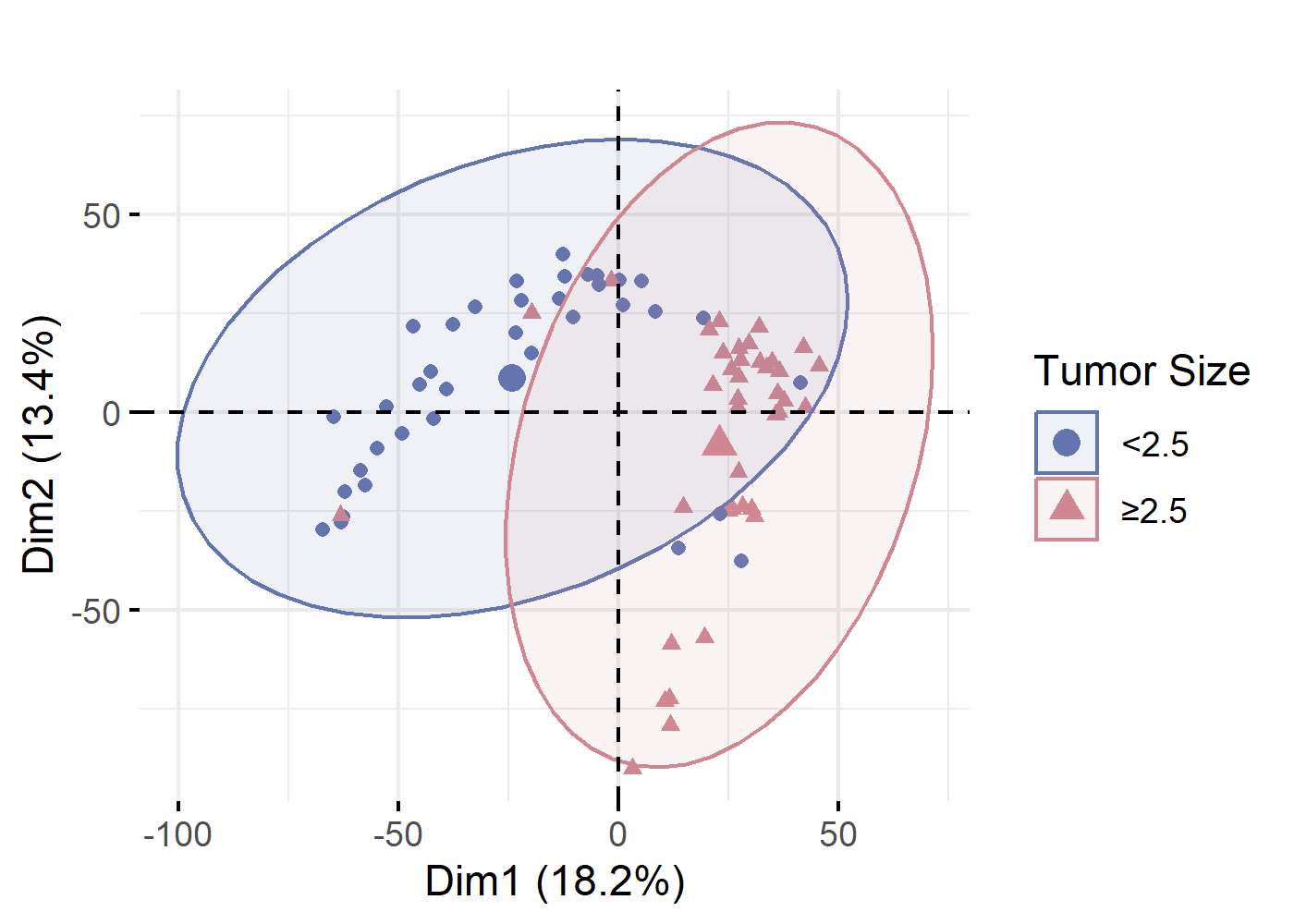
B**

**Figure S5.**


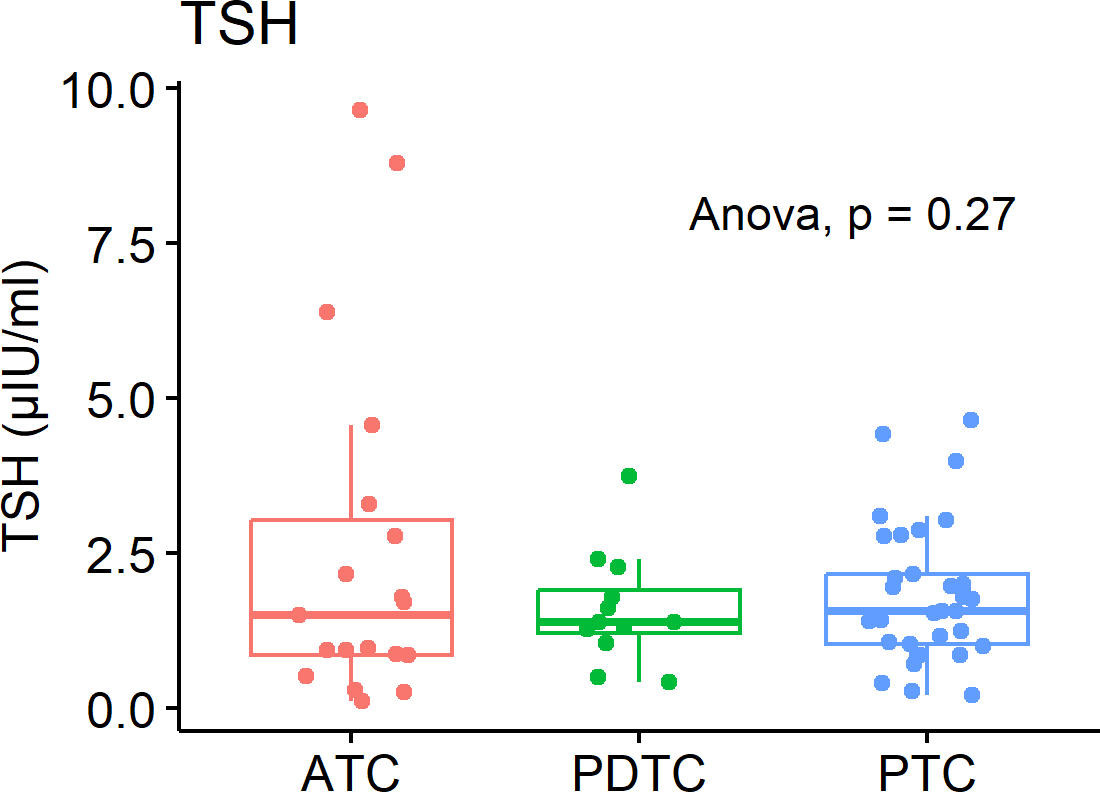

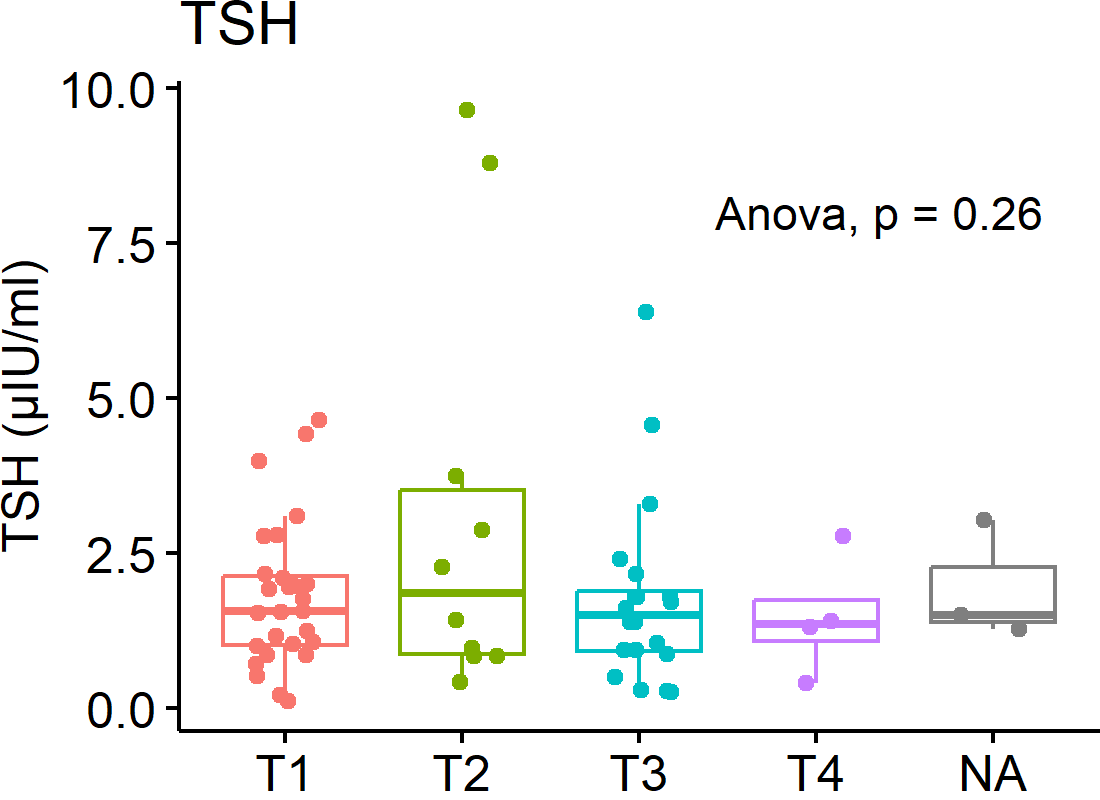
**A**


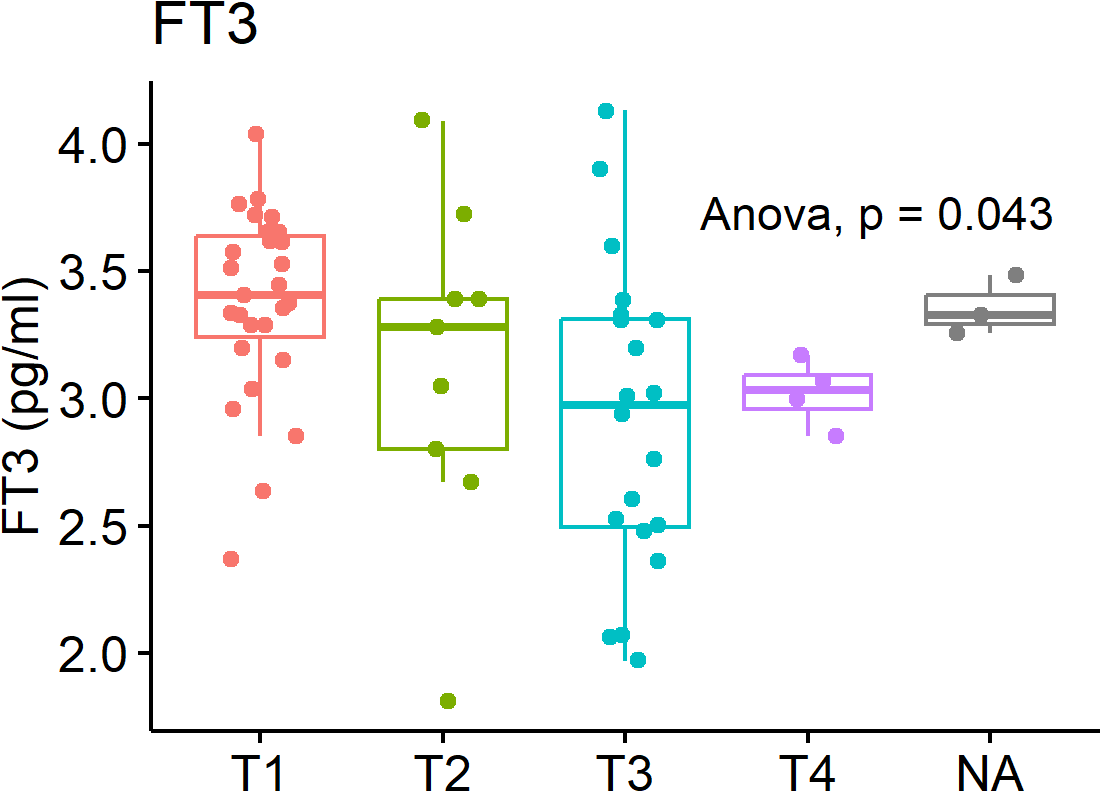

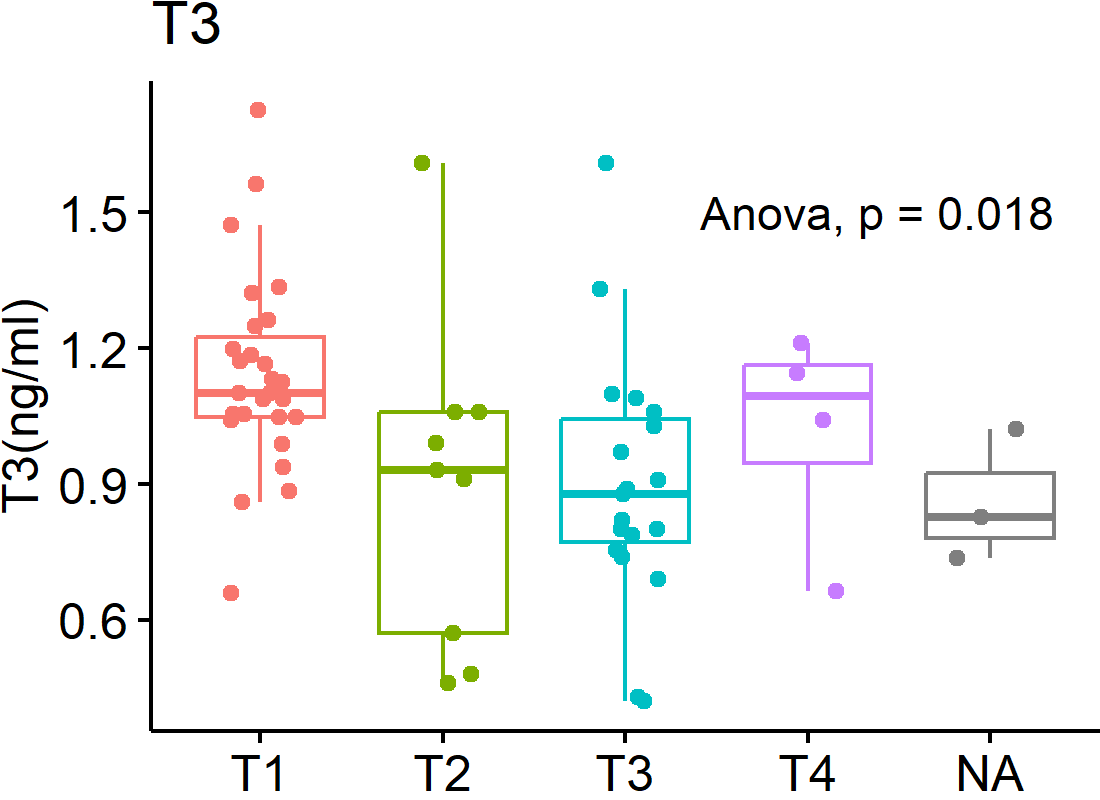
**B**


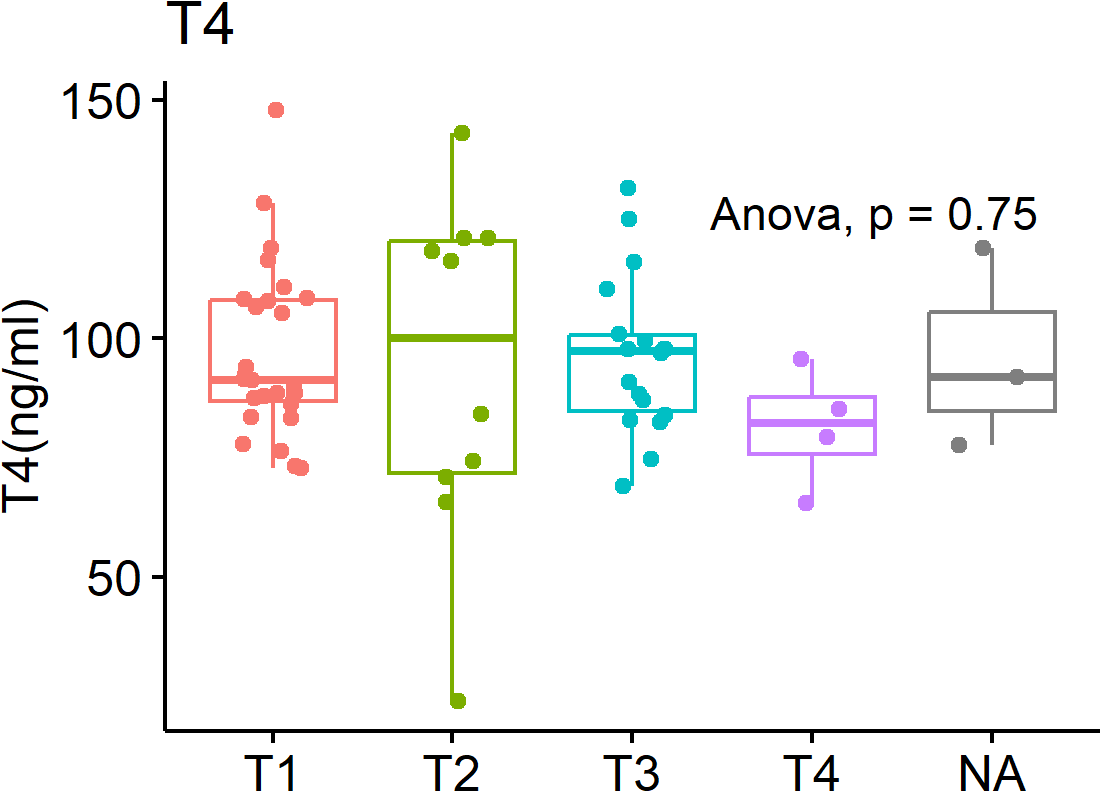

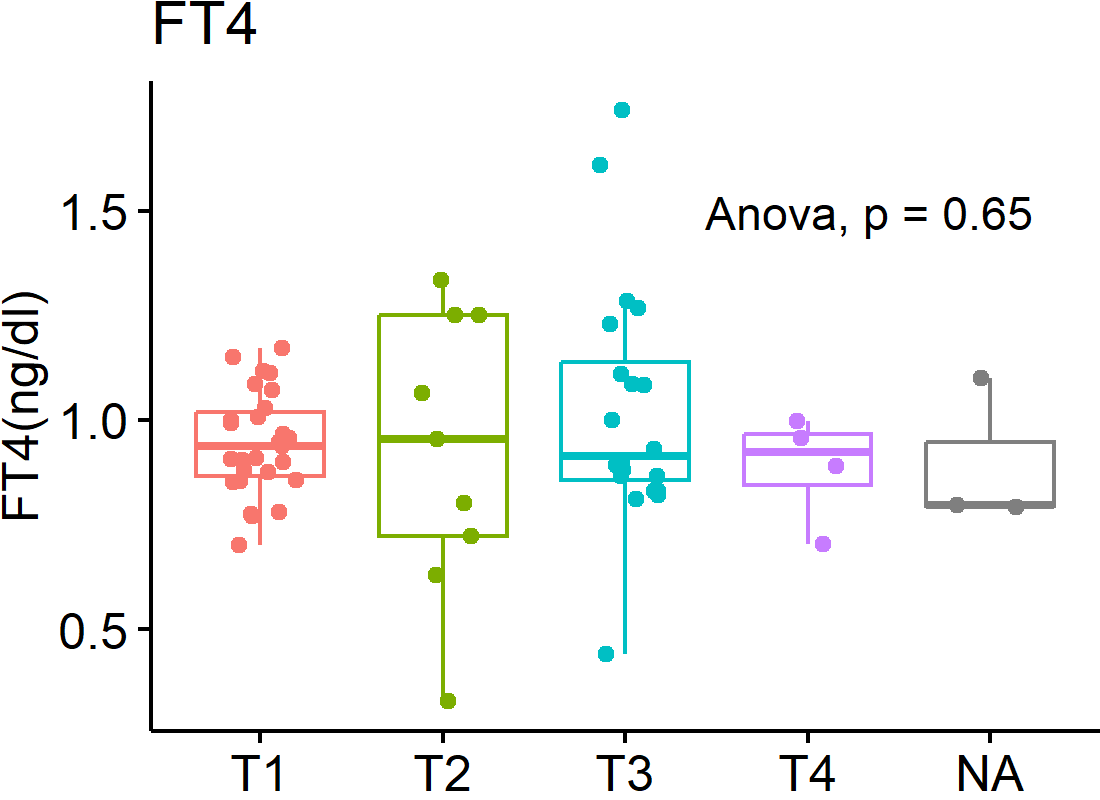


**C**


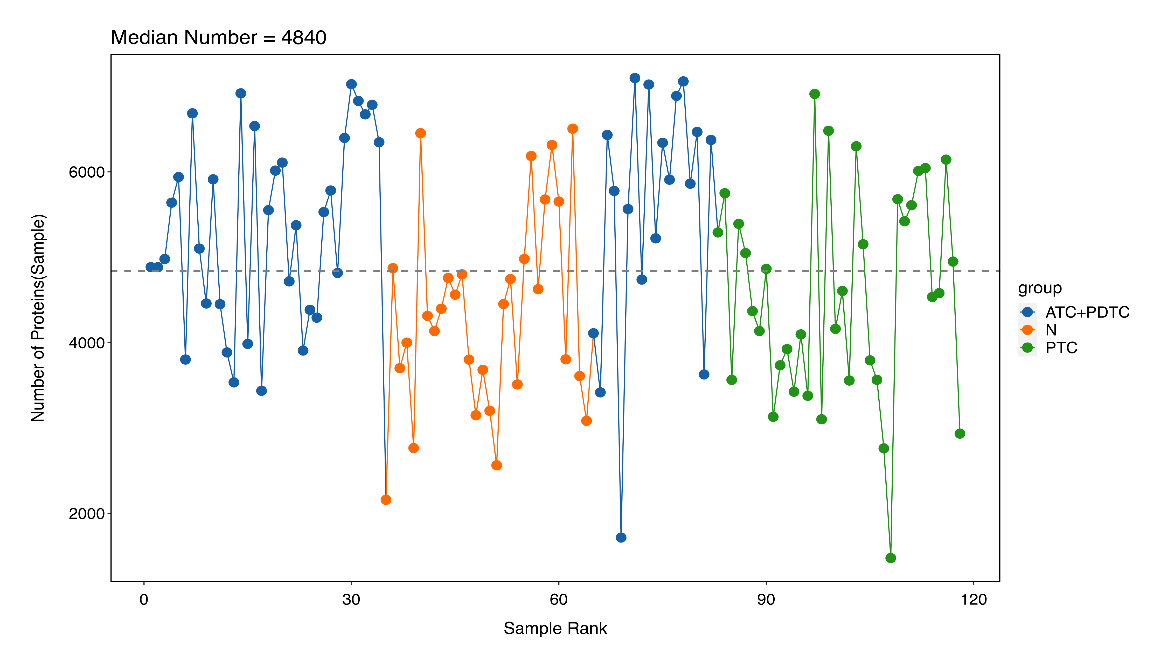


**ATC**

**N**

**PDTC**

**PTC**

**Figure S6.**

1.
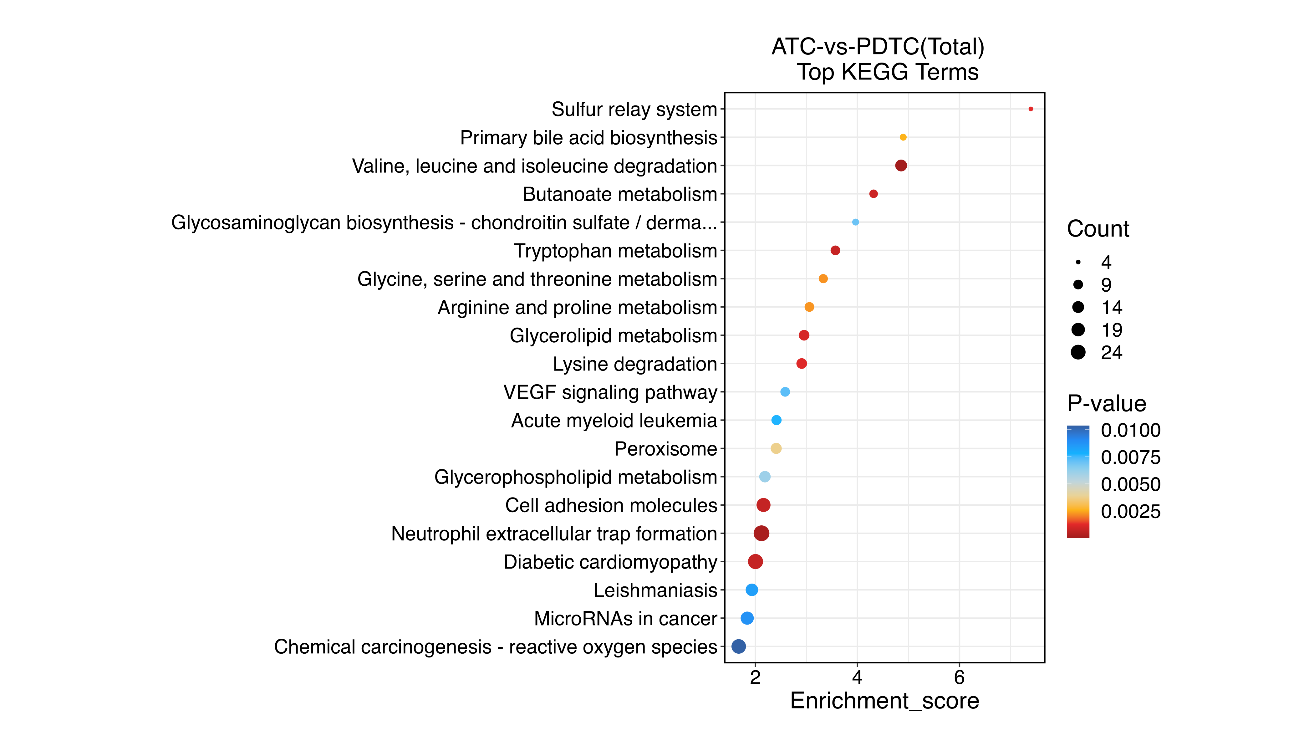
**ATC VS PDTC**
2. **
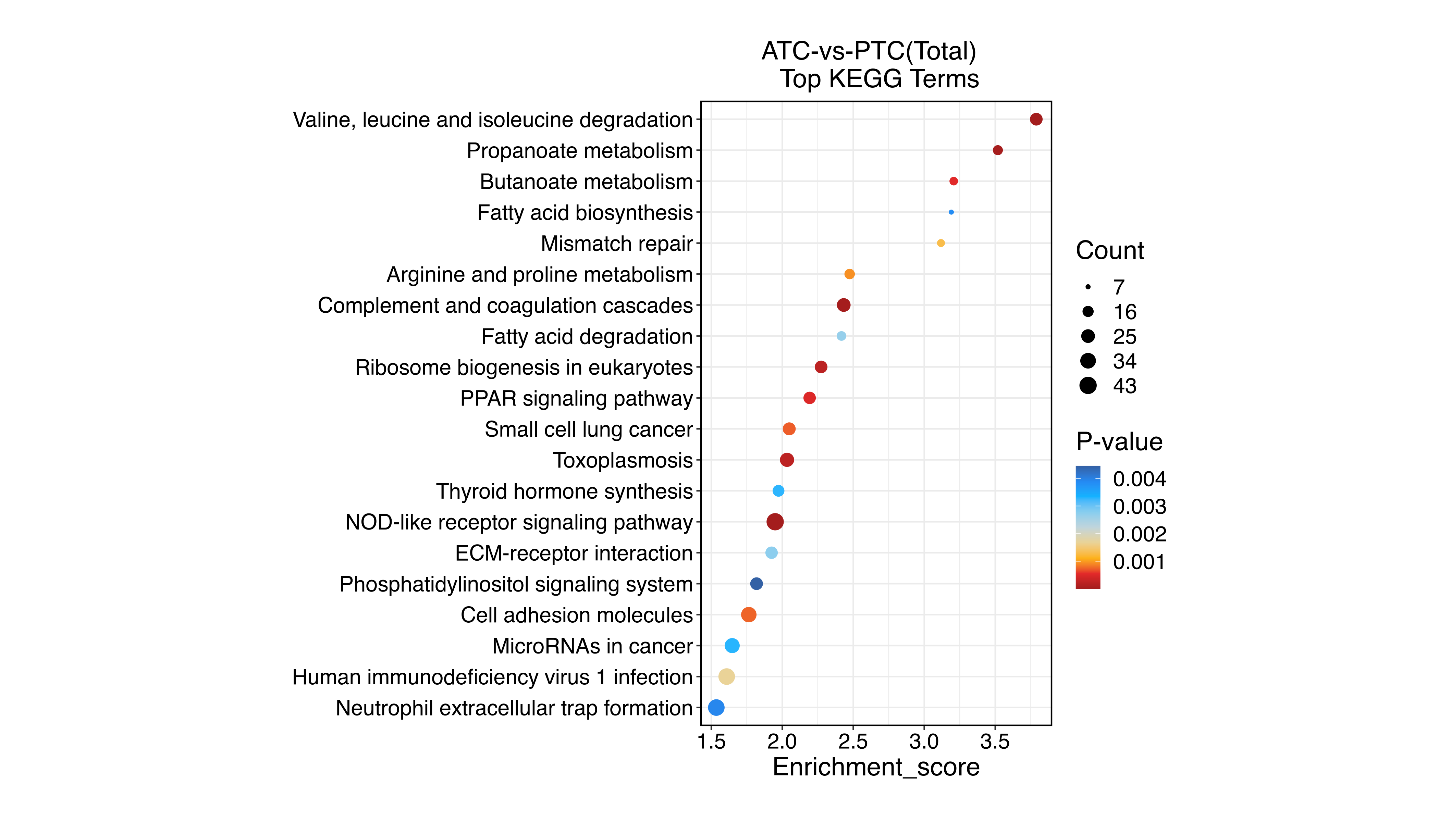
ATC VS PTC**

**Figure S6 (continued).**

1. **
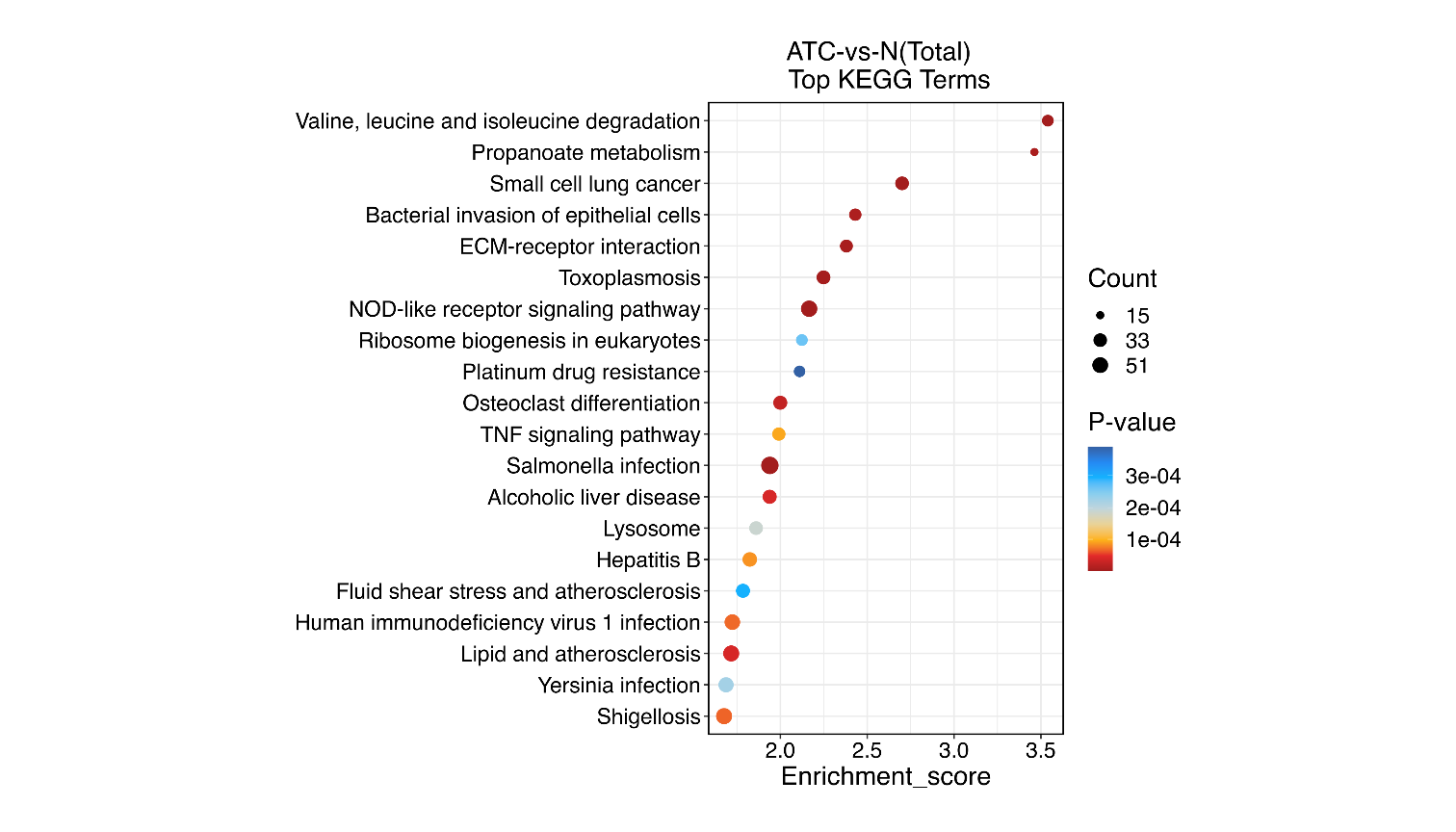
ATC VS N**

**
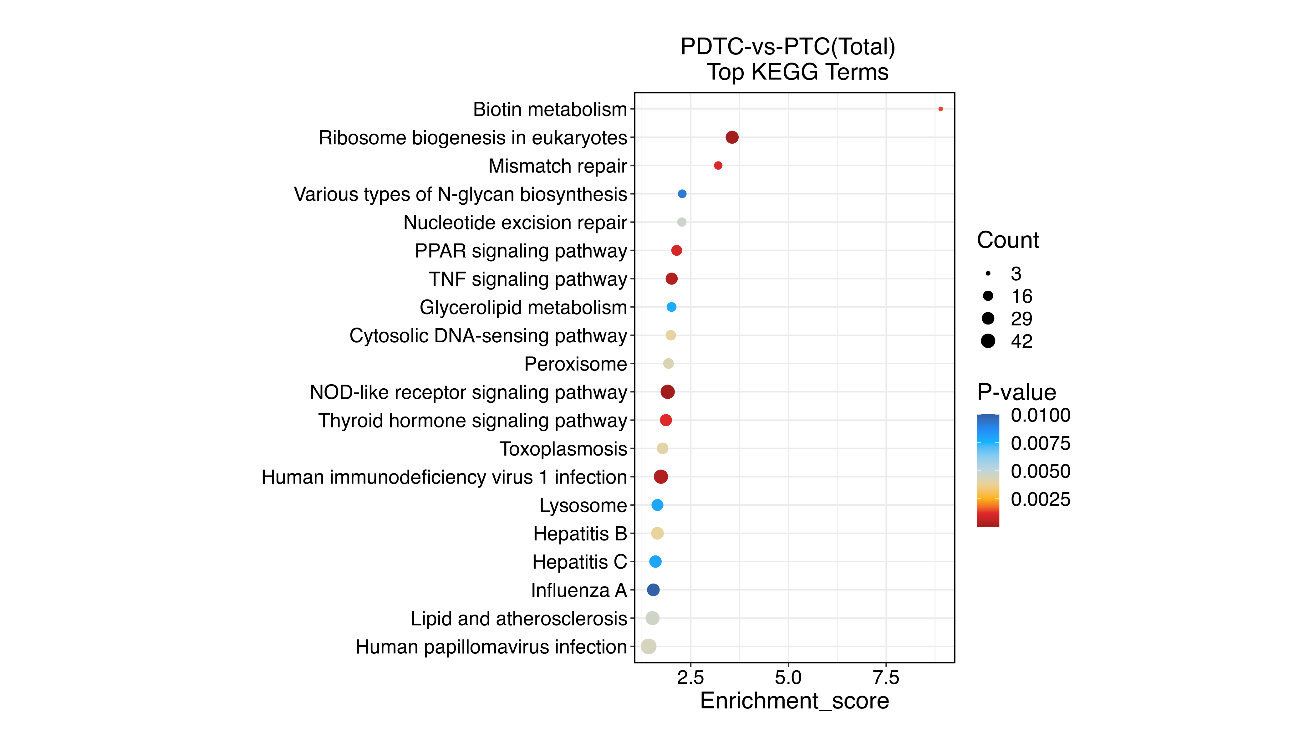
D. PDTC VS PTC**

**Figure S6 (continued).**

**
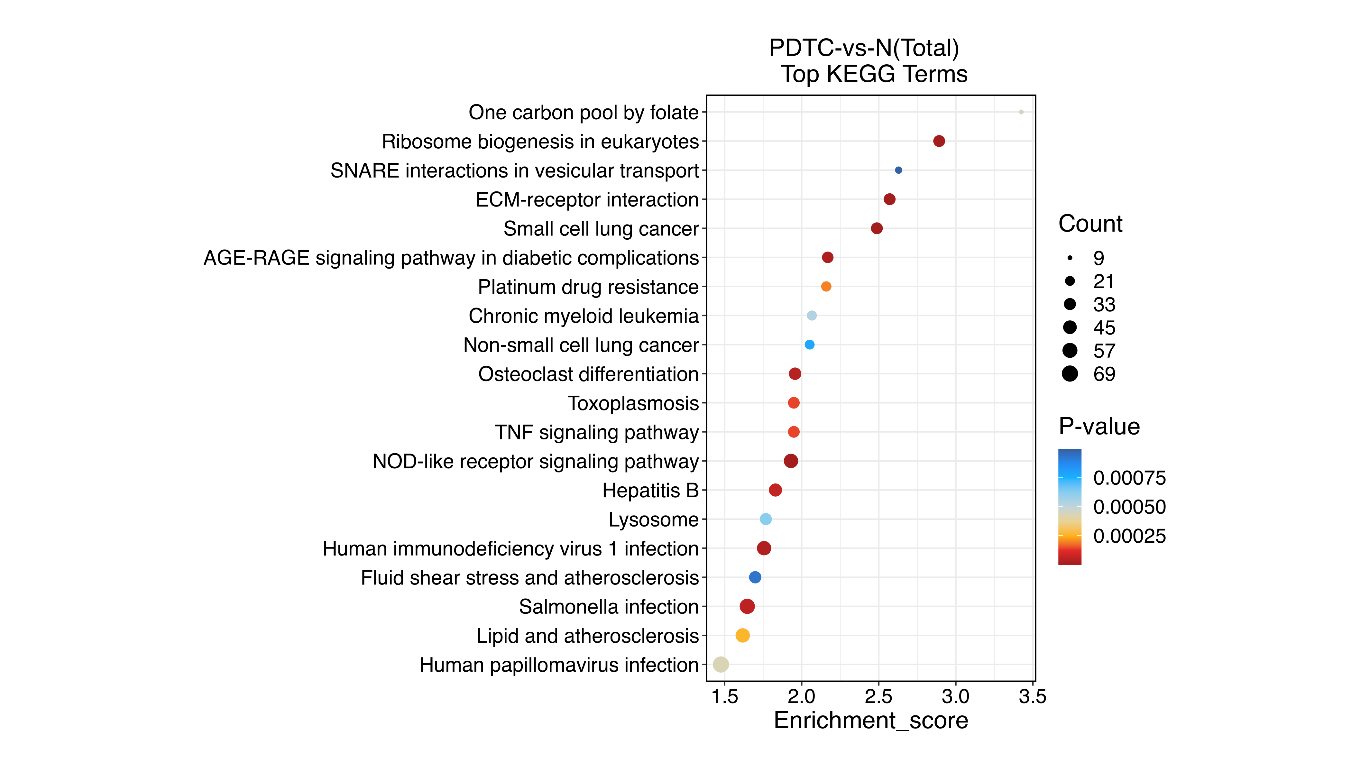
E. PDTC VS N**

**
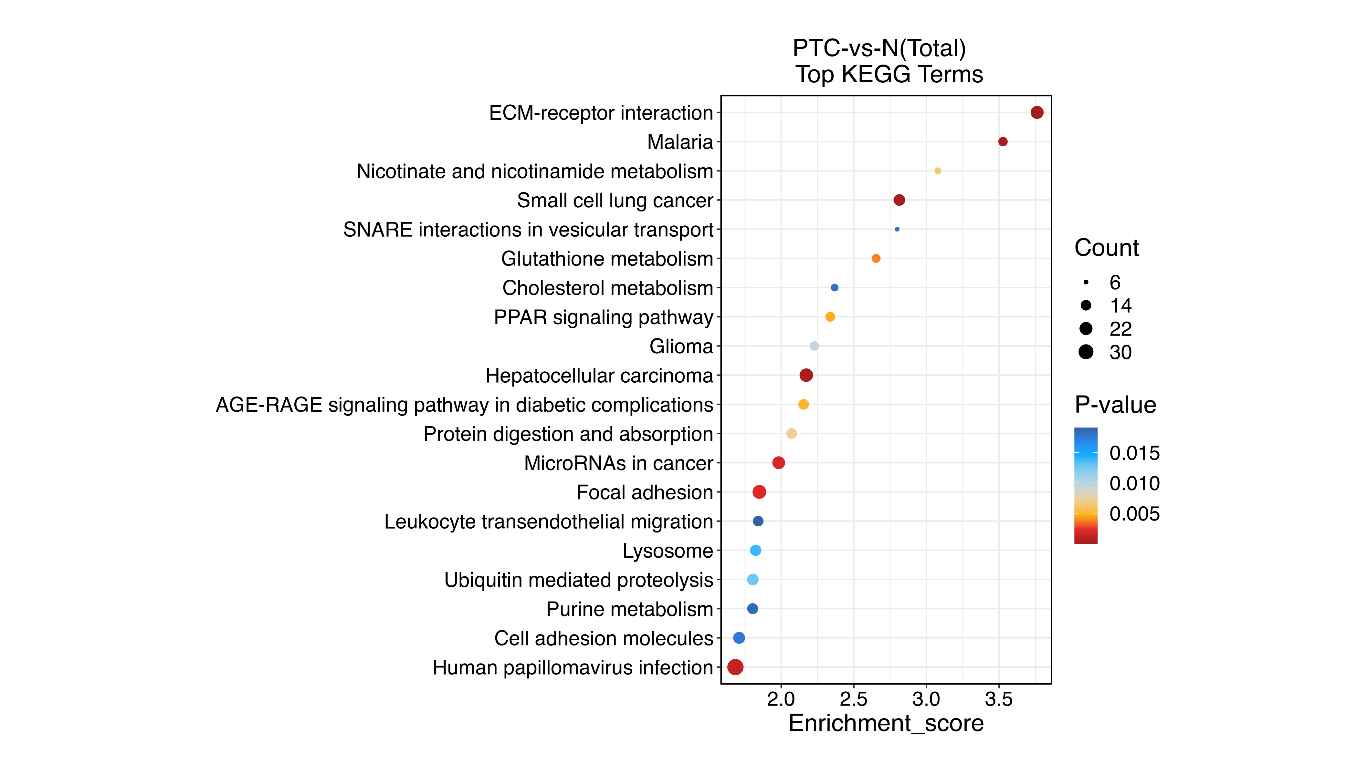
F. PTC VS N**

**Figure S7.**

**
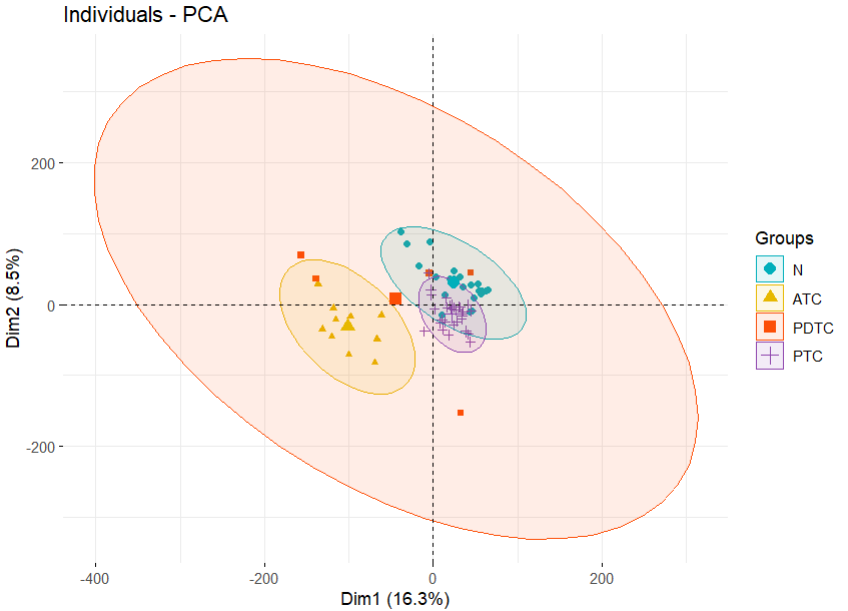
A**

**
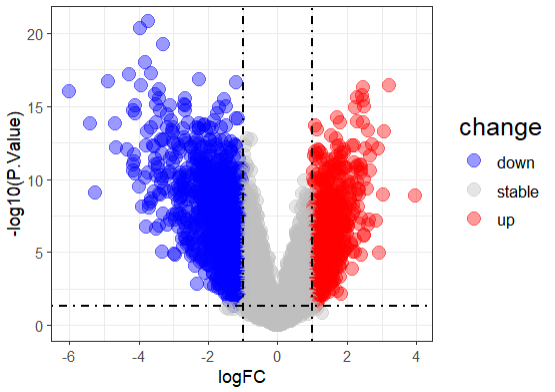
**

**B**

**Figure S8.**

**
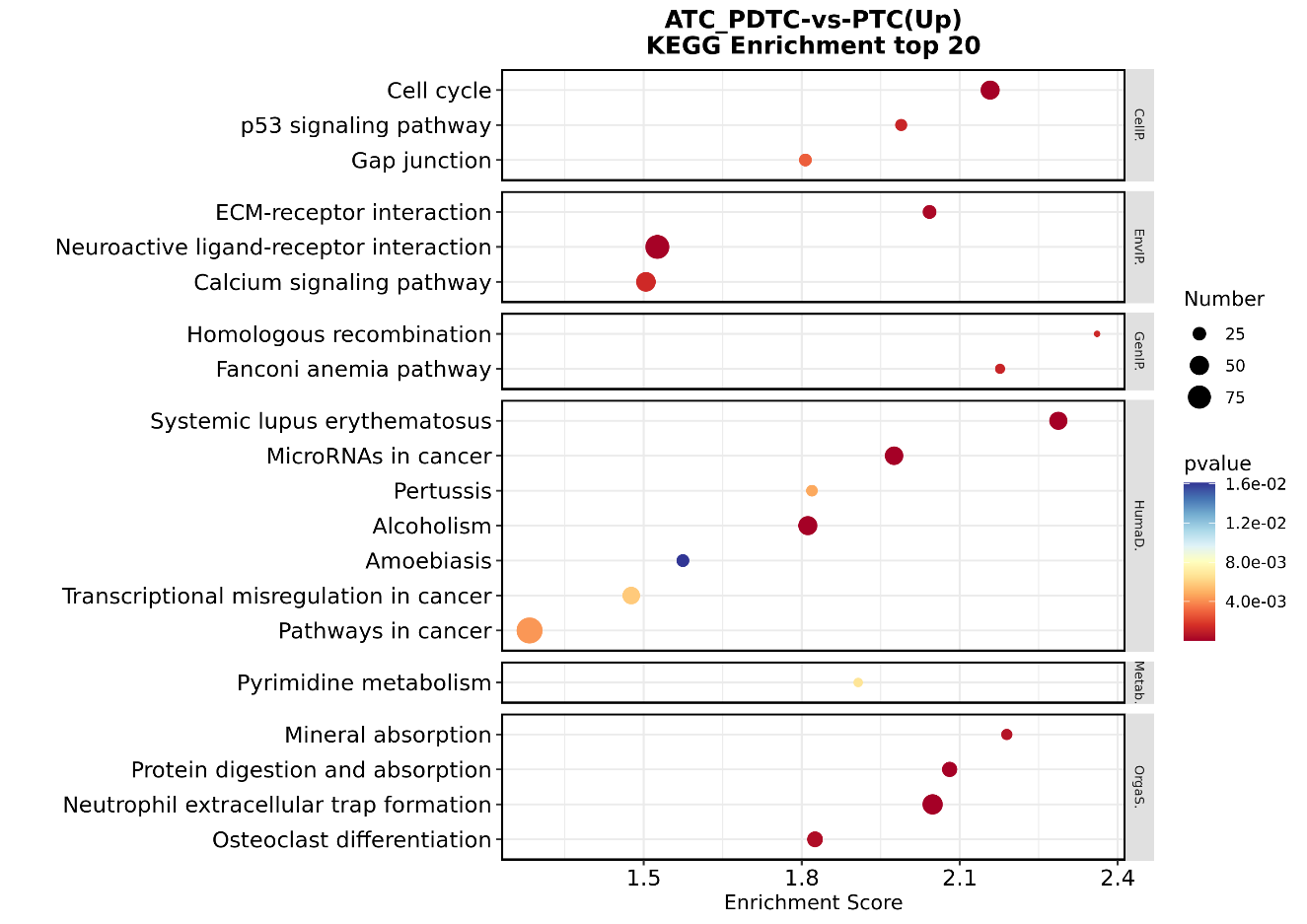
A**

**
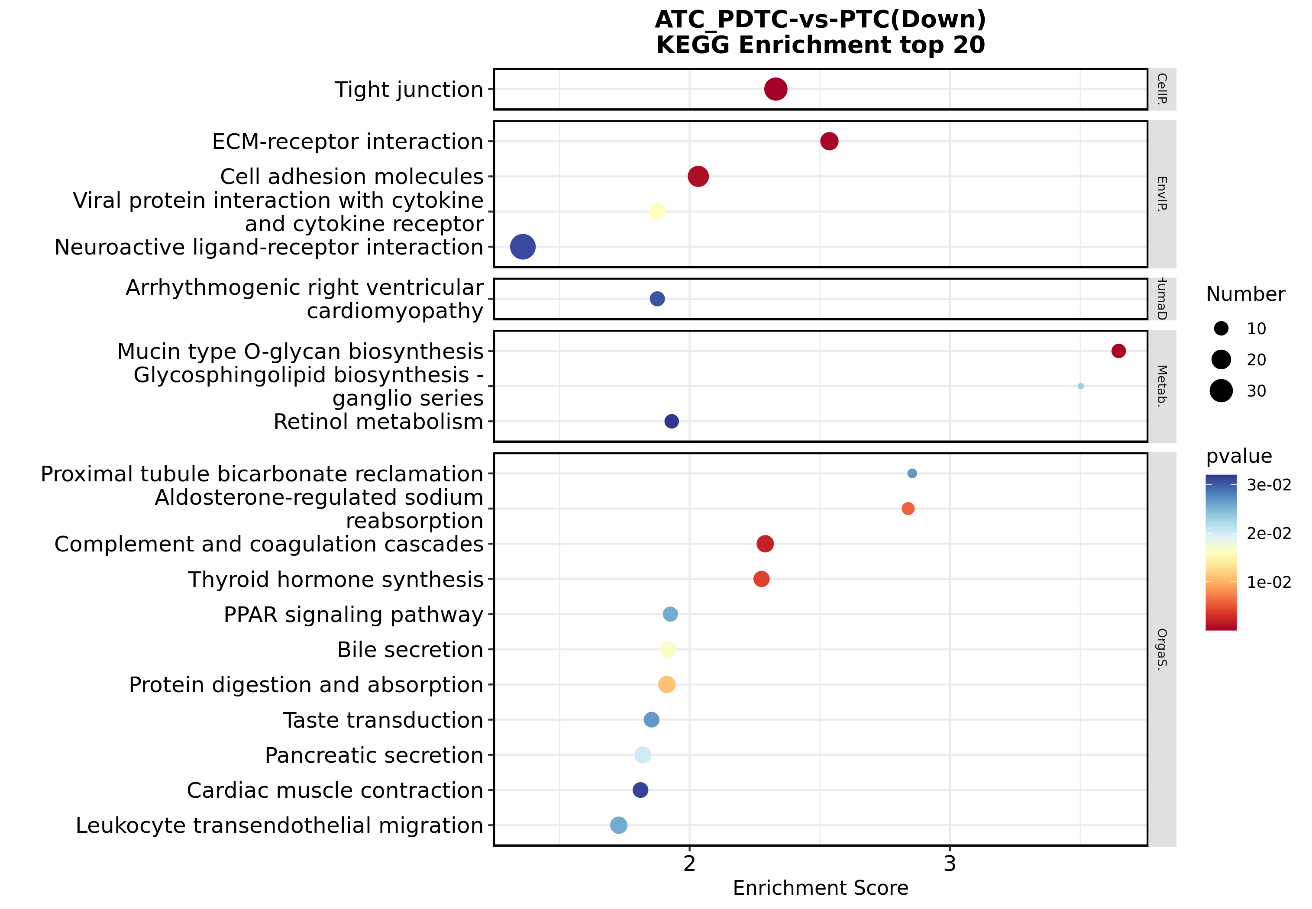
B**

**Figure S9.**

**
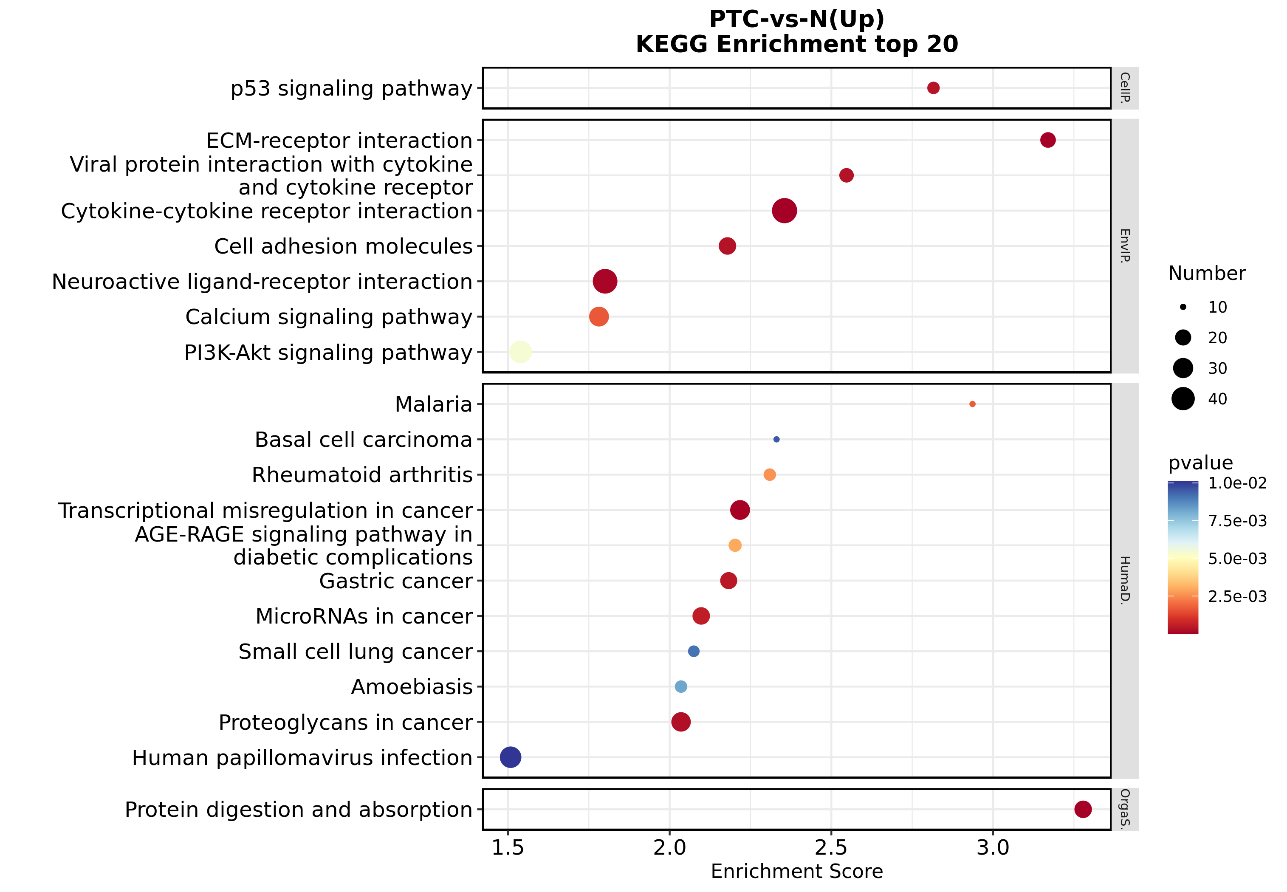
A**

**
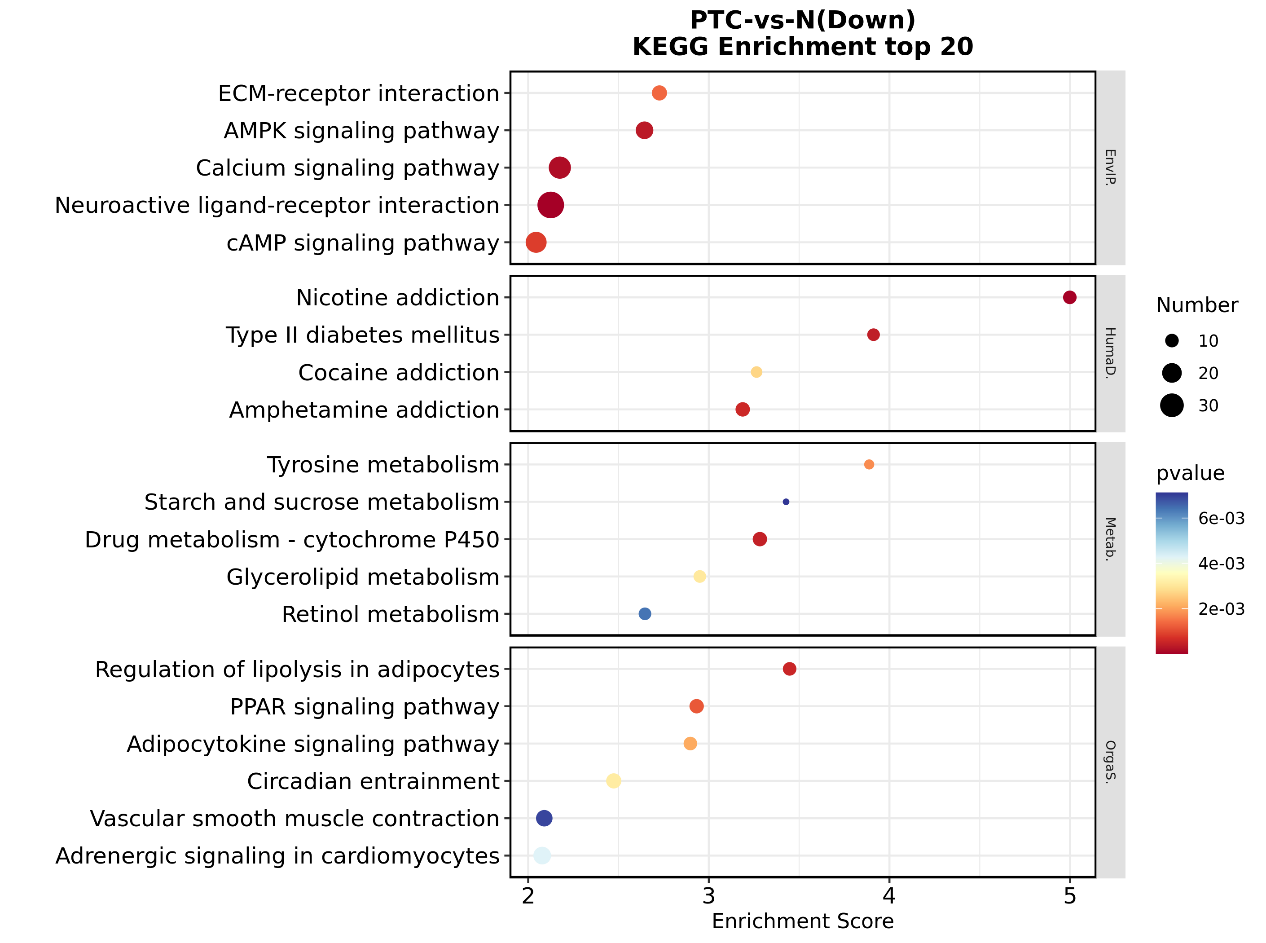
B**

**Figure S10.**

1. **ATC vs N**

**DEG (down)**

**DEG (up)**

**
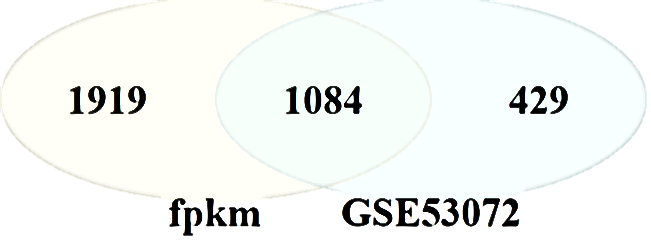

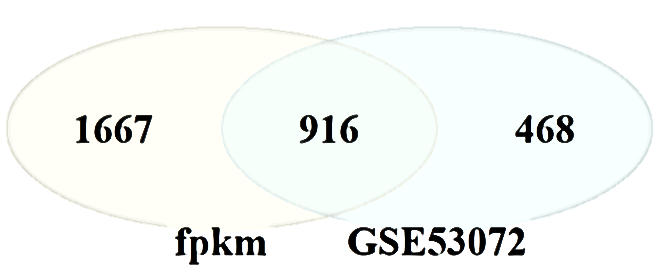

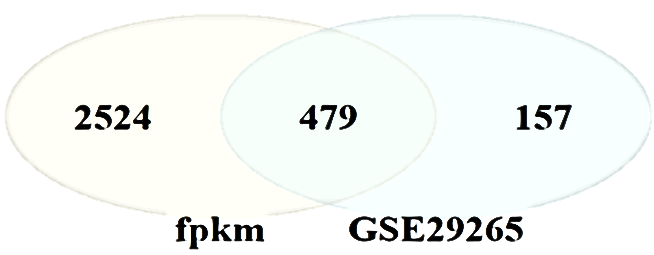

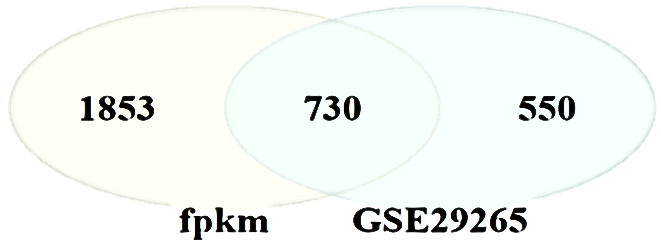

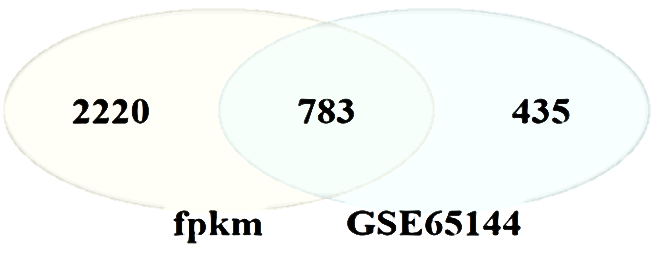

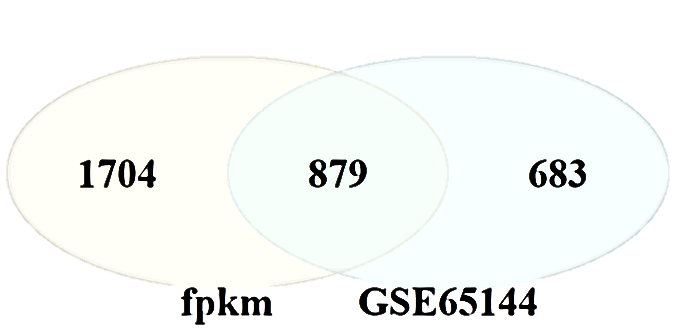

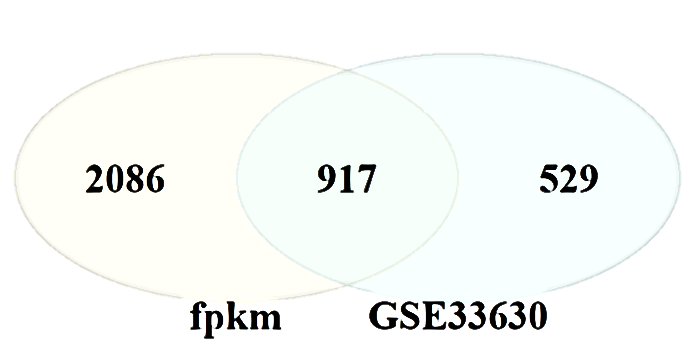
**
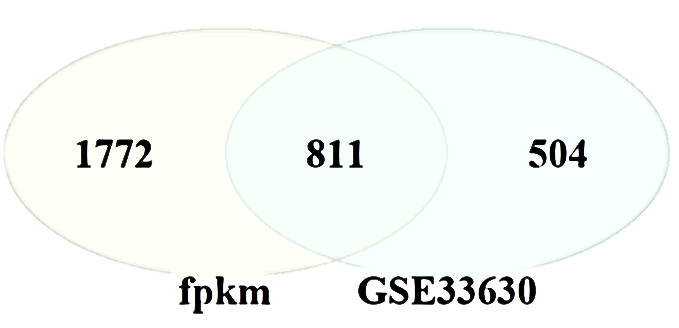


**Figure S10 (continued).**

1. **ATC VS PTC**

**DEG (up)**

**DEG (down)**

**
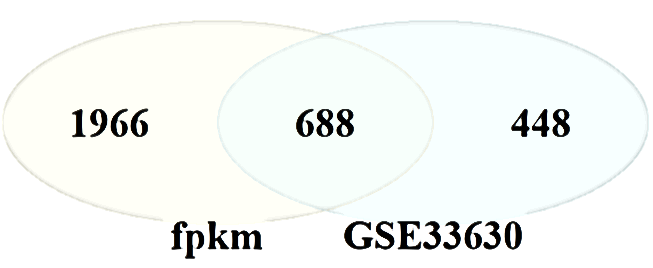

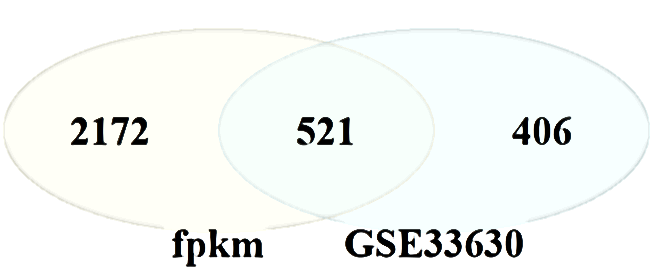
**

**
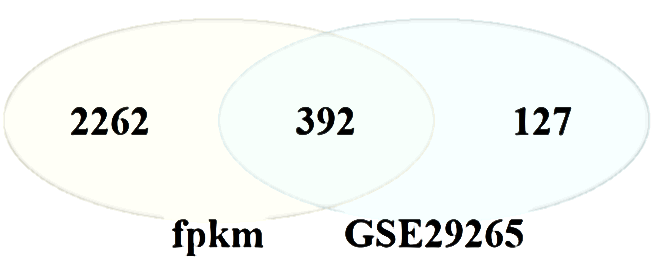

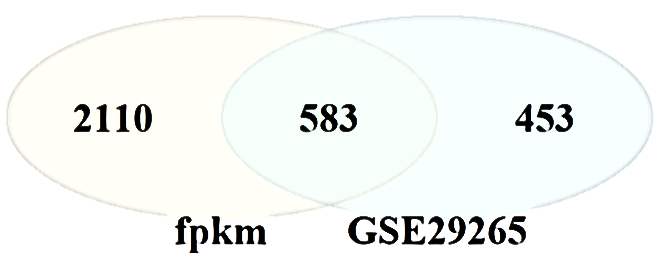
**

1. **PTC VS N**

**DEG (down)**

**DEG (up)**

**
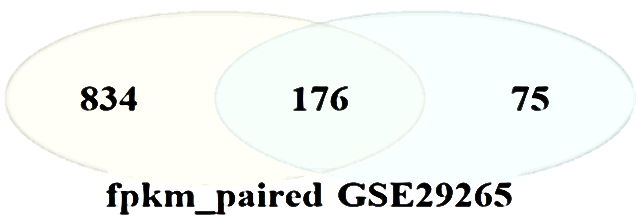

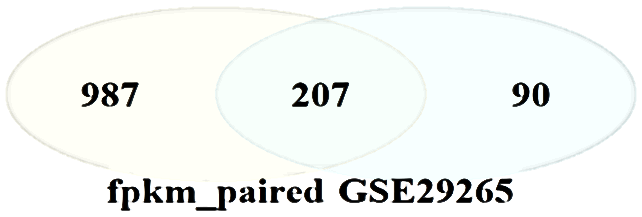
**

**
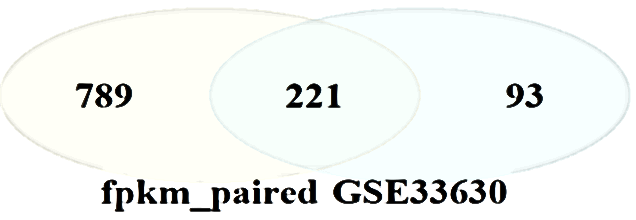

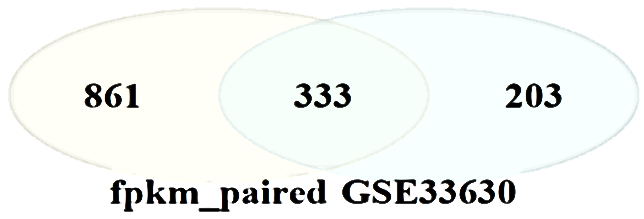
**

1. **Paired PTC VS N**

**DEG (up)**

**DEG (down)**

**
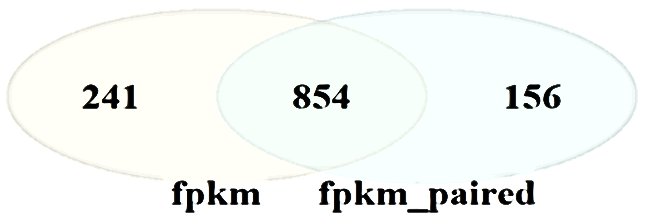
**
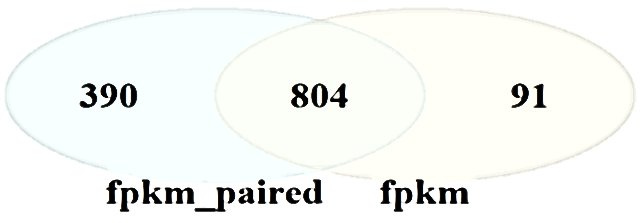


**Figure S11.**

**
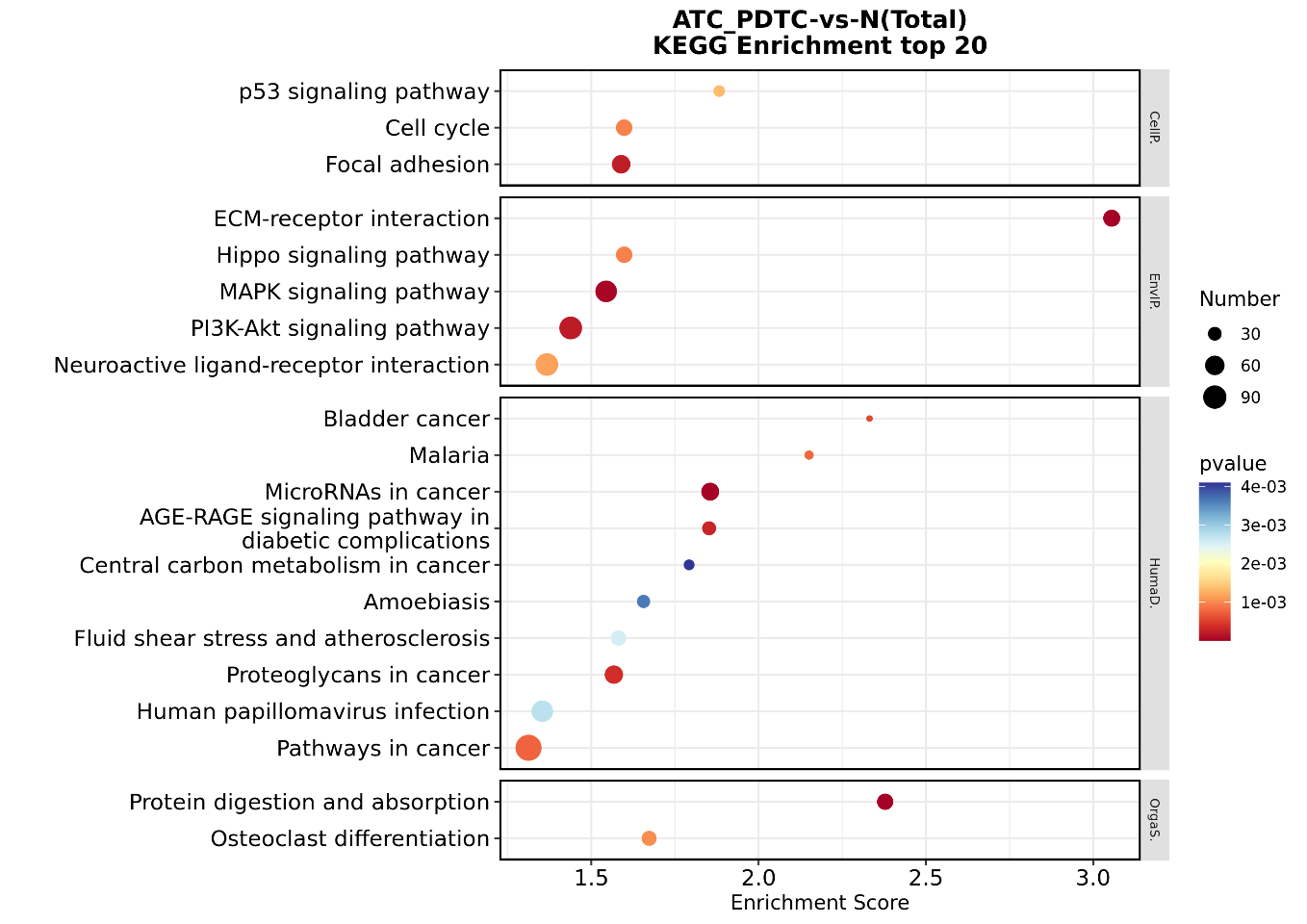
A**

**
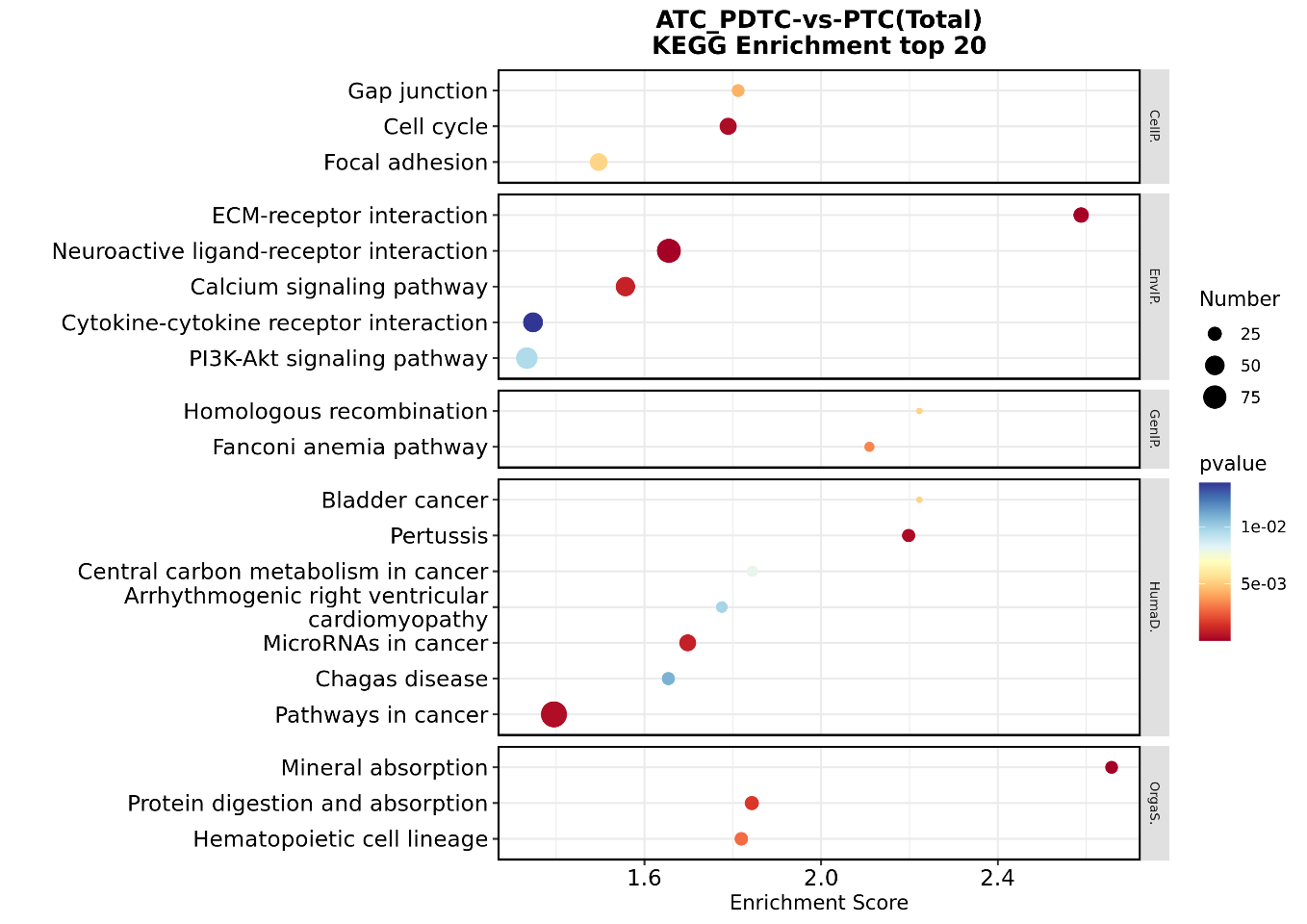
B**


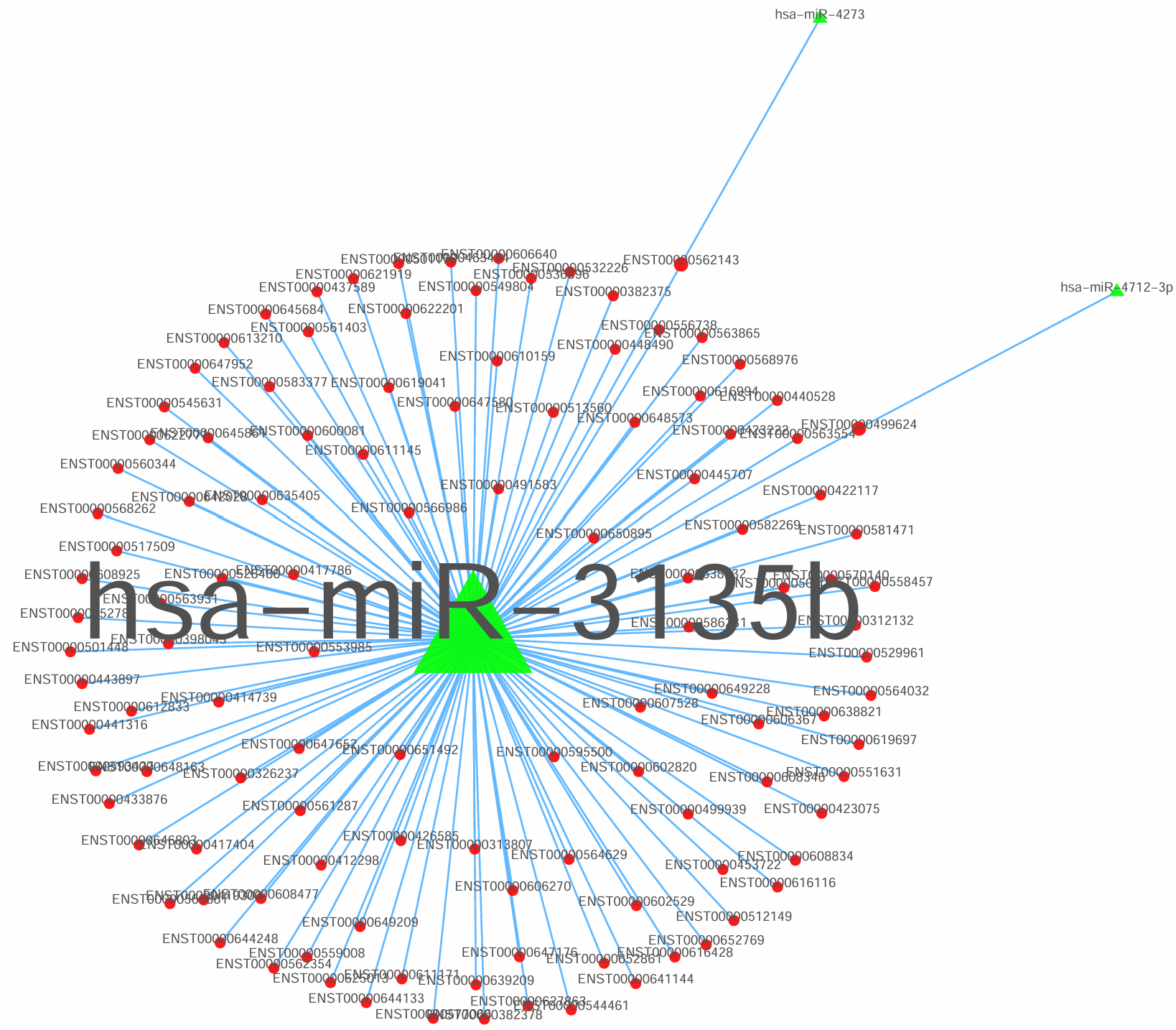
**Figure S12.**

**A. ATC-PDTC vs N**

**Figure S12 (continued).**

**B. ATC-PDTC vs PTC**

**
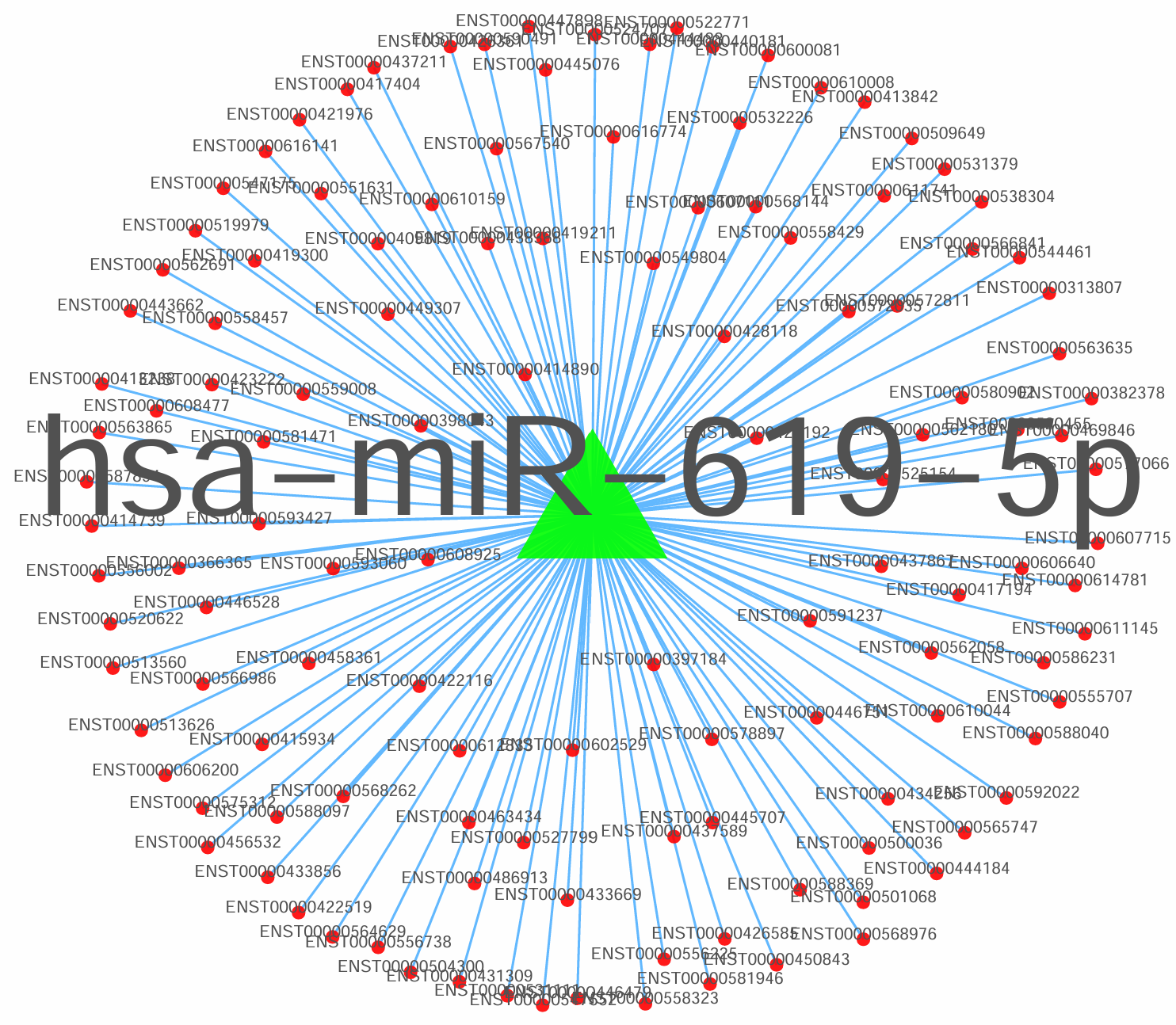
**


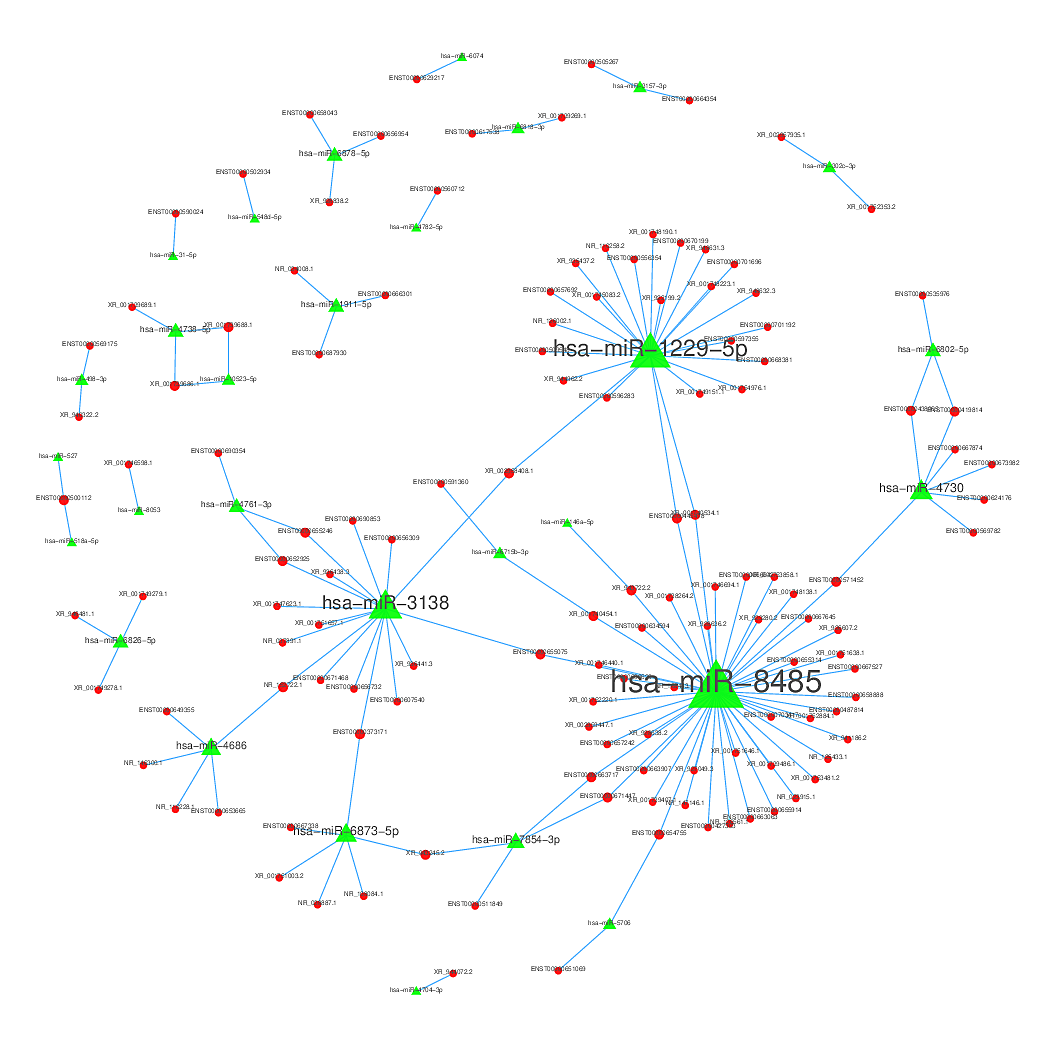
**Figure S12 (continued).**

**C. ATC vs PDTC**

**Figure S13.**

1.
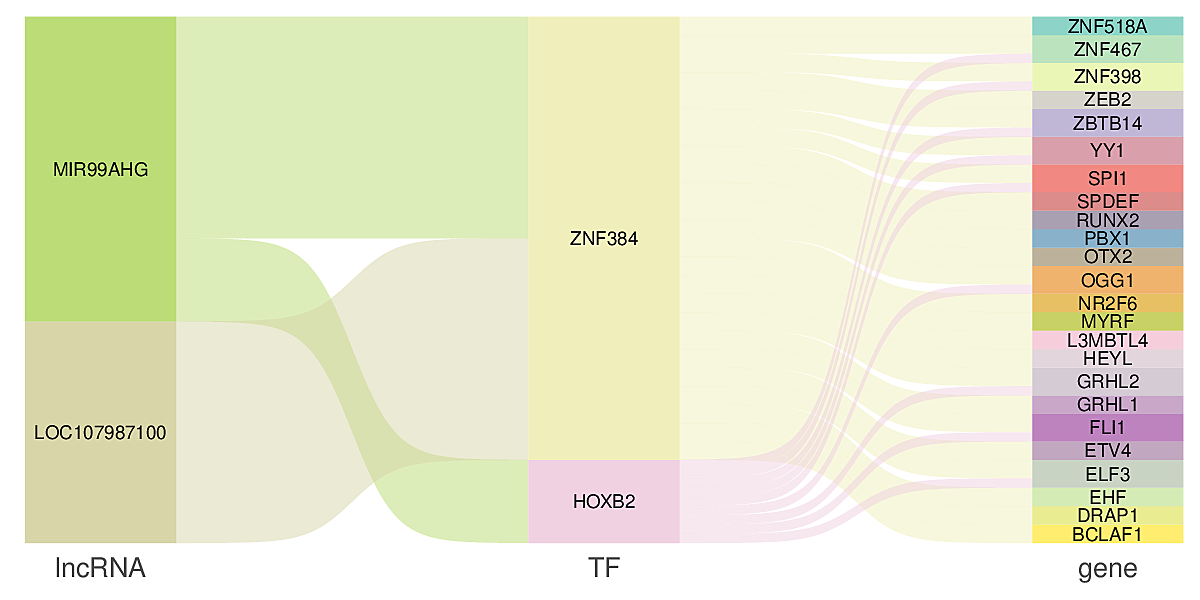
**ATC vs PDTC**

**Figure S13 (continued).**

1. **
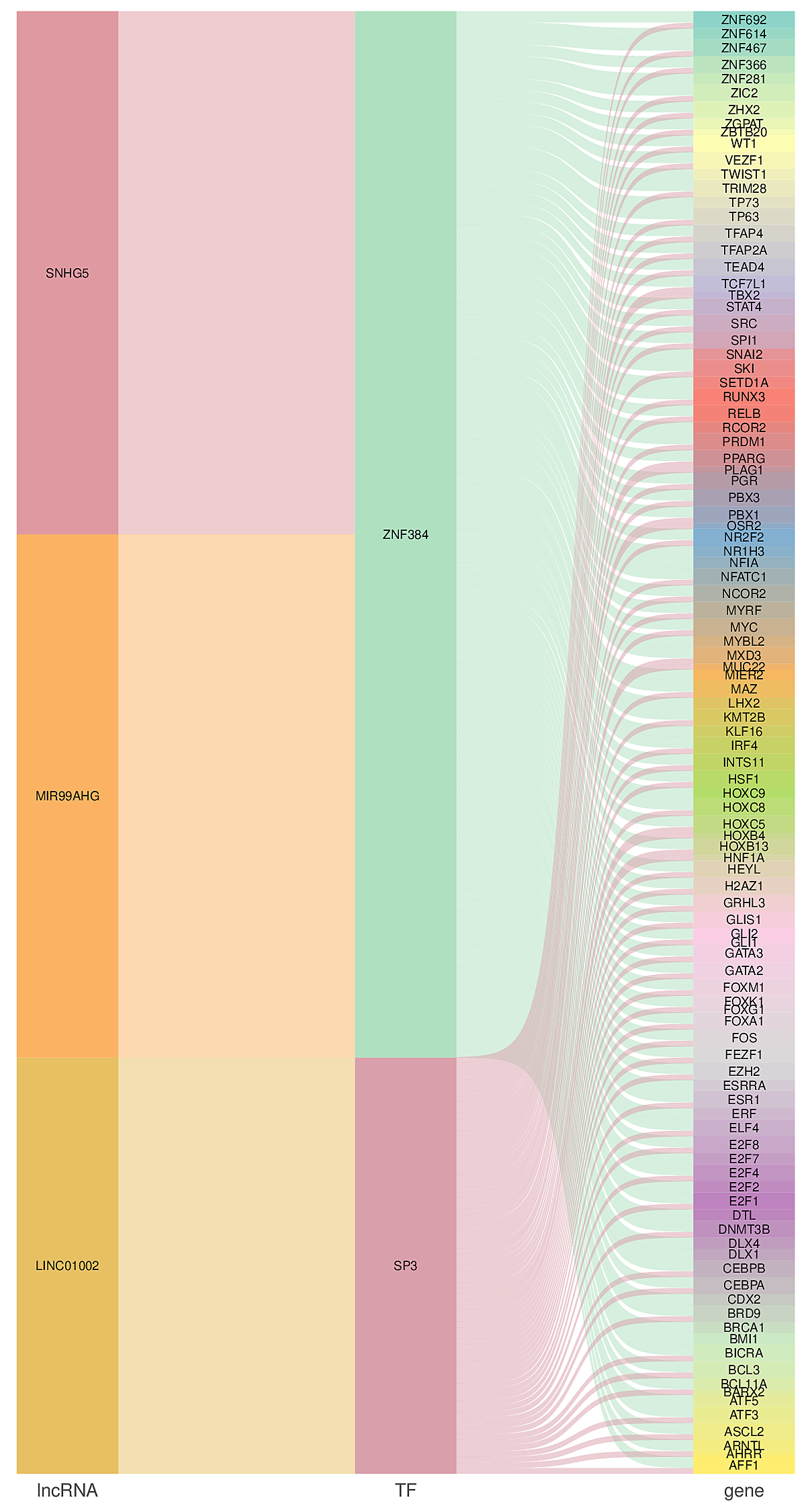
ATC-PDTC vs PTC**

**
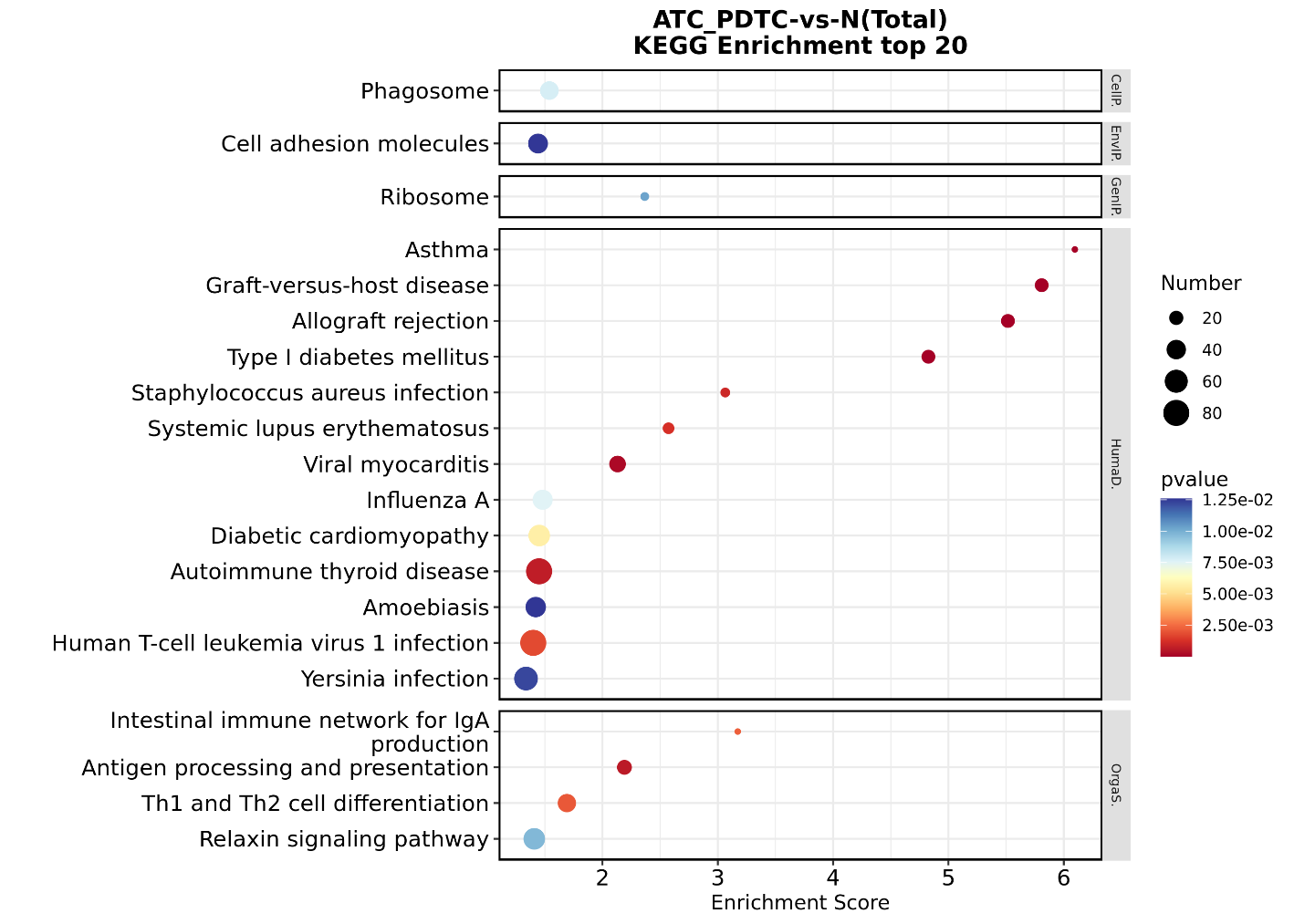
Figure S14**

**A**

**
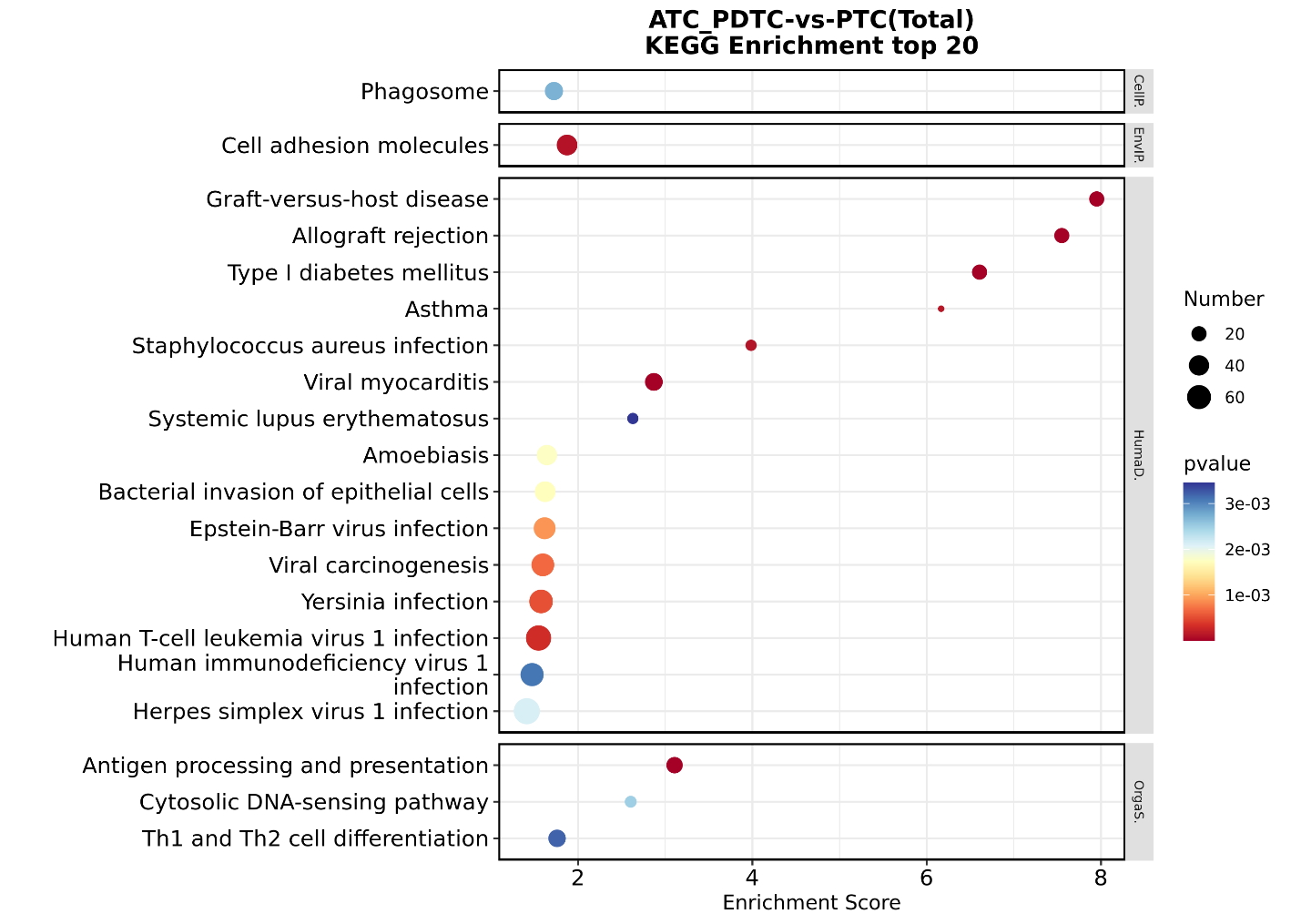
**

**B**


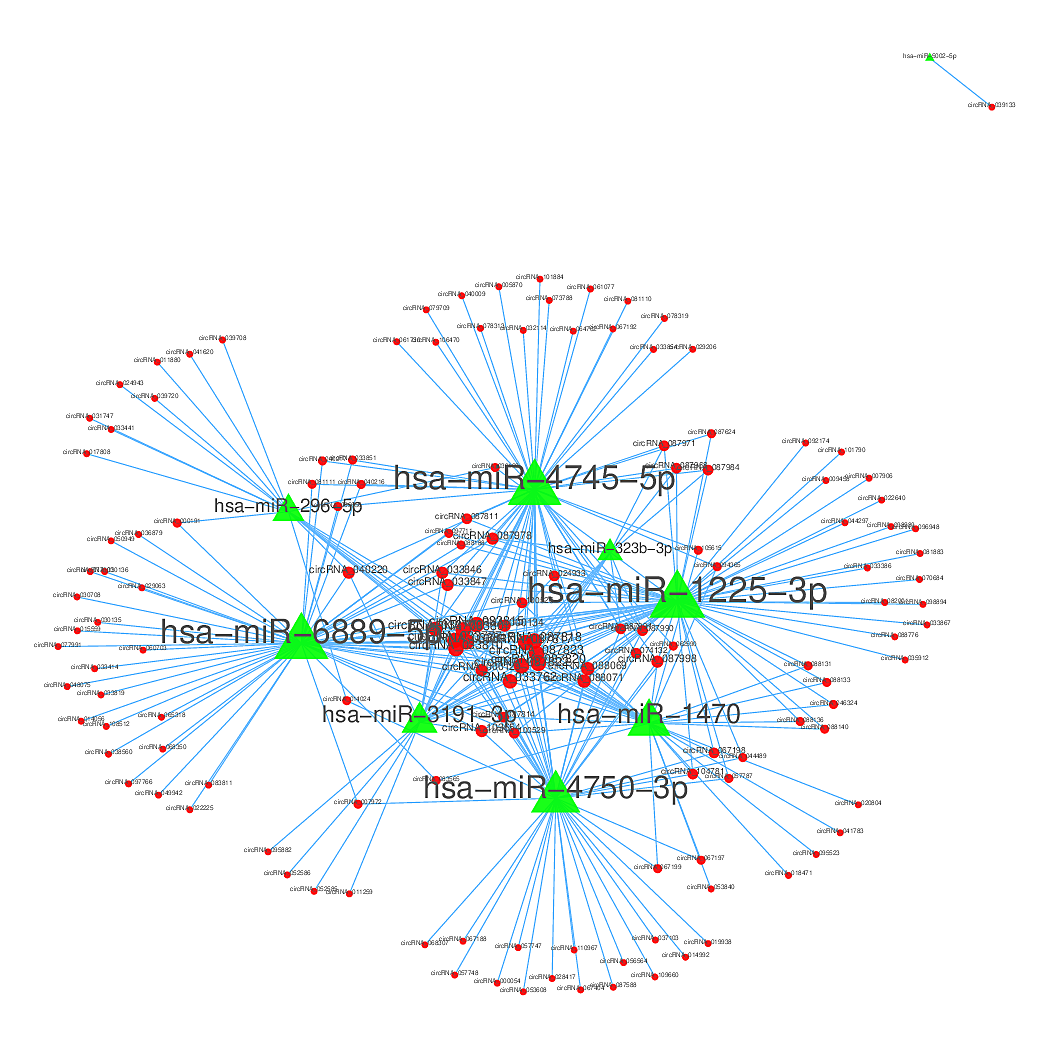
**Figure 15.**

1. **ATC-PDTC vs PTC**


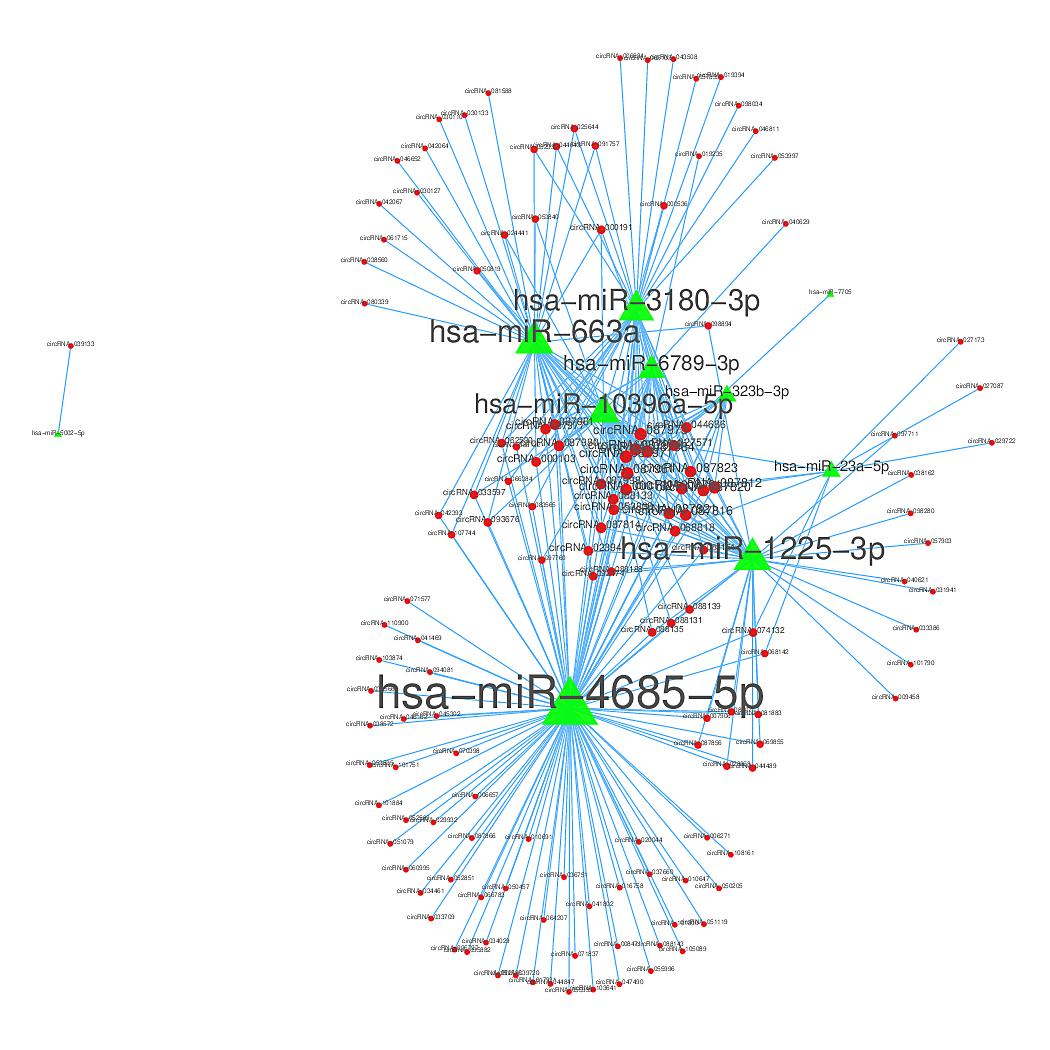
**Figure 15 (continued).**

**B. ATC vs PDTC**

**
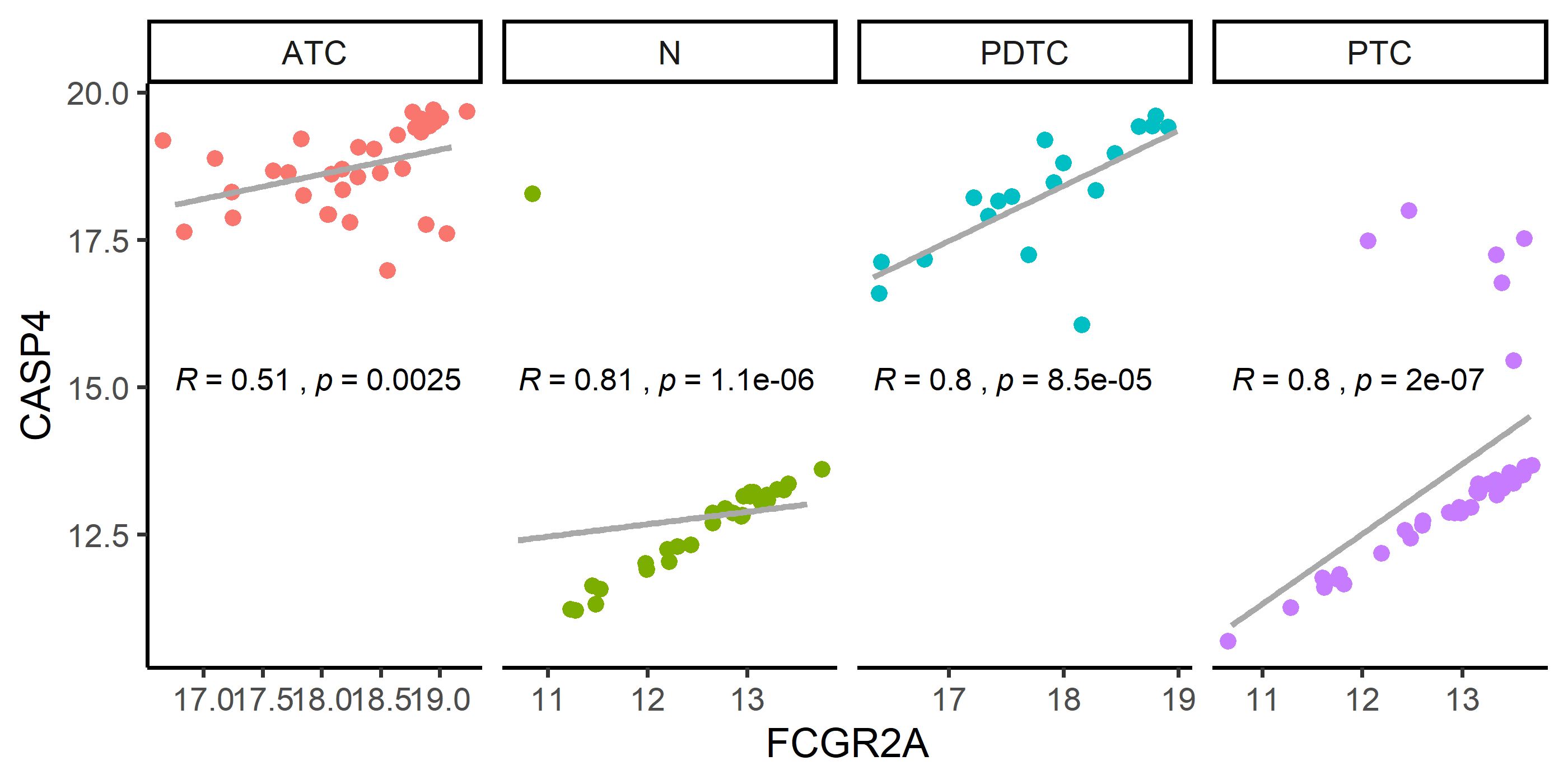
Figure S16.**

**
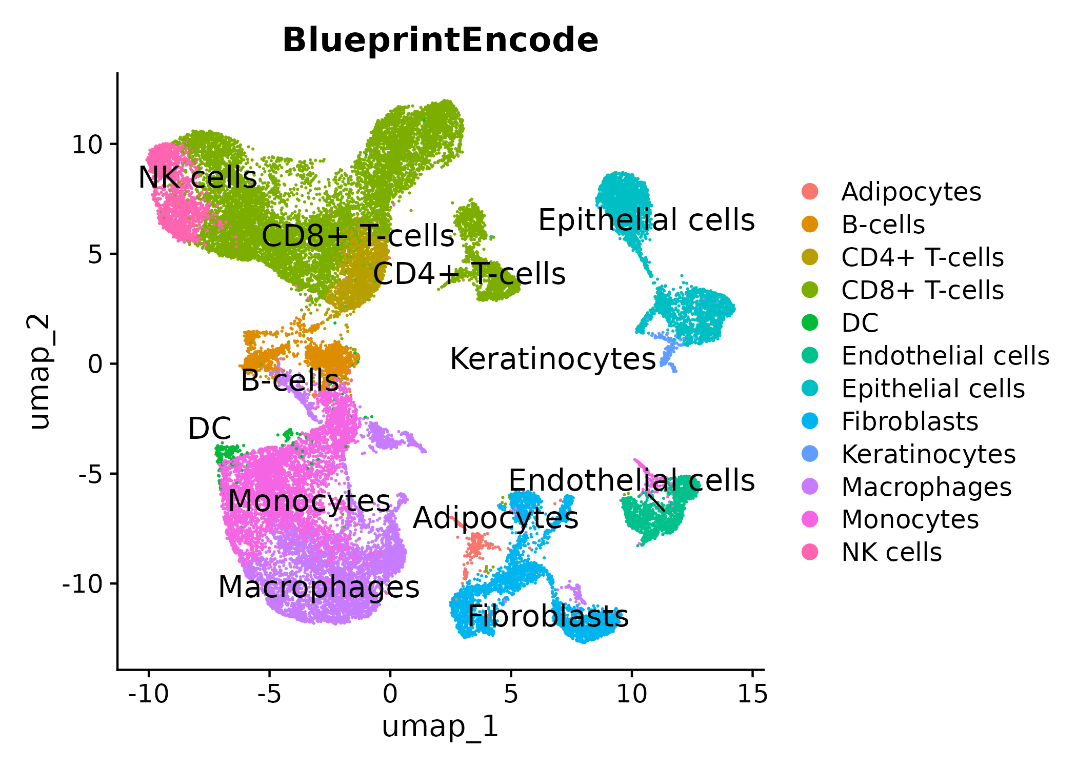
Figure 17.**

**A**

**
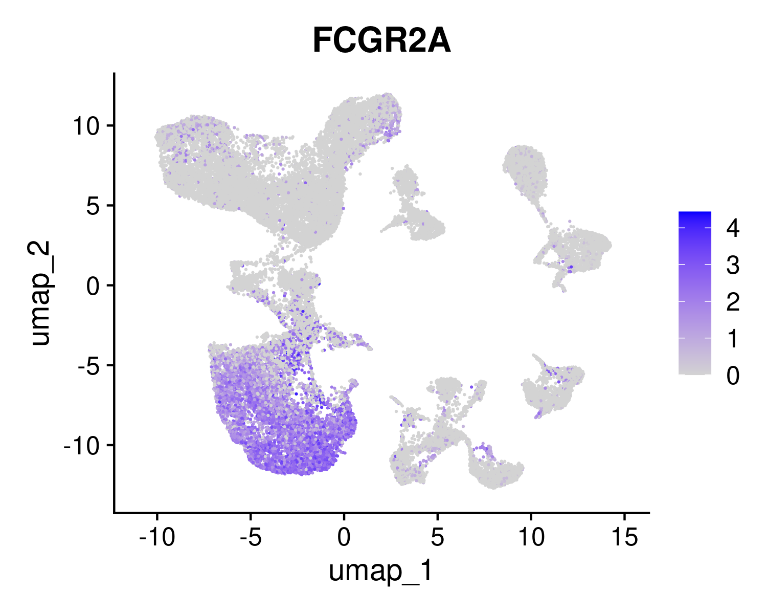
B**

**
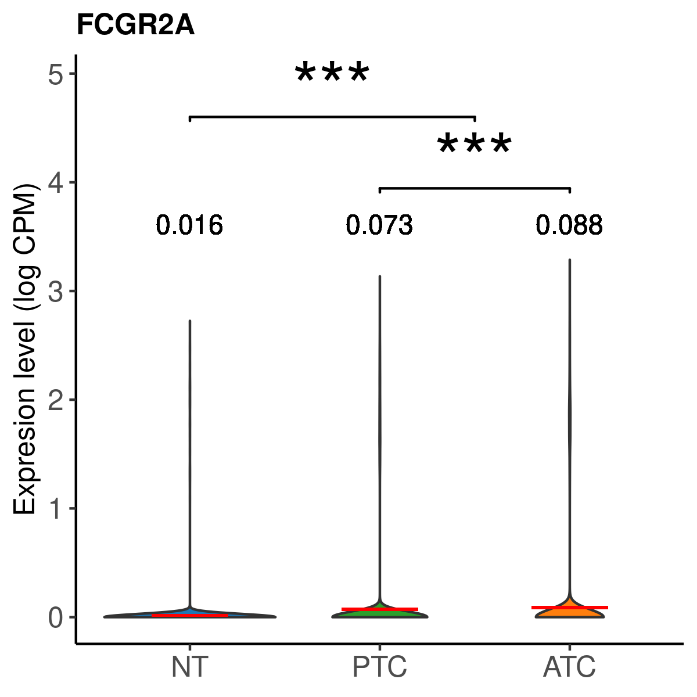
**

**C**


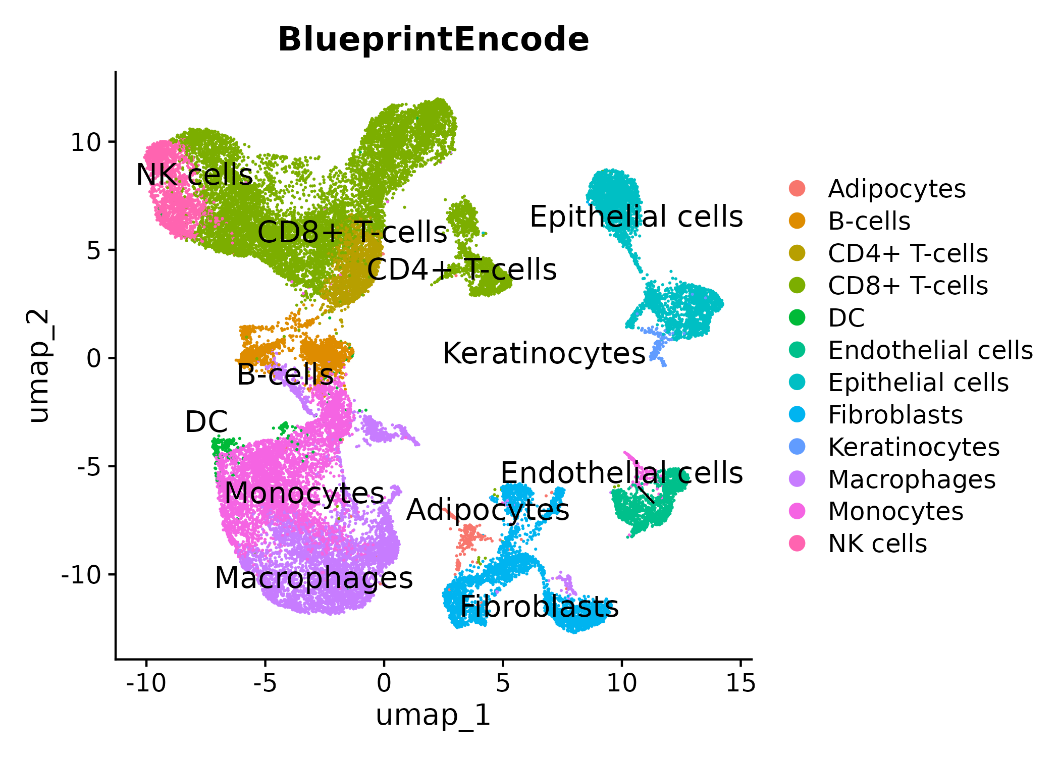
**Figure 18.**

**A**


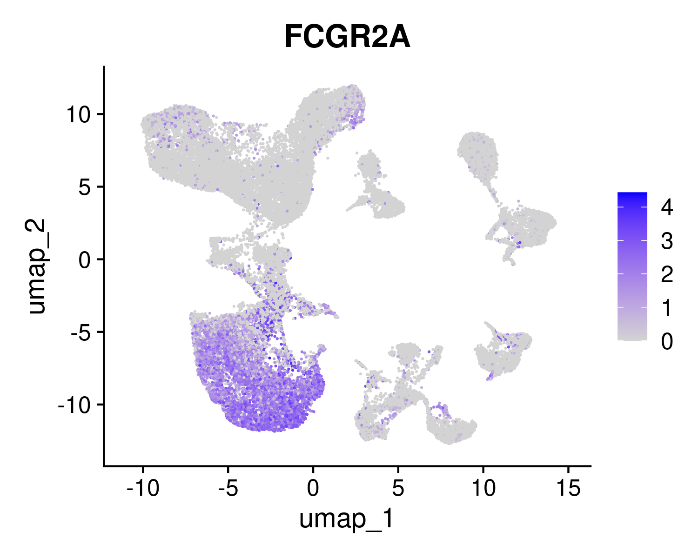


**B**


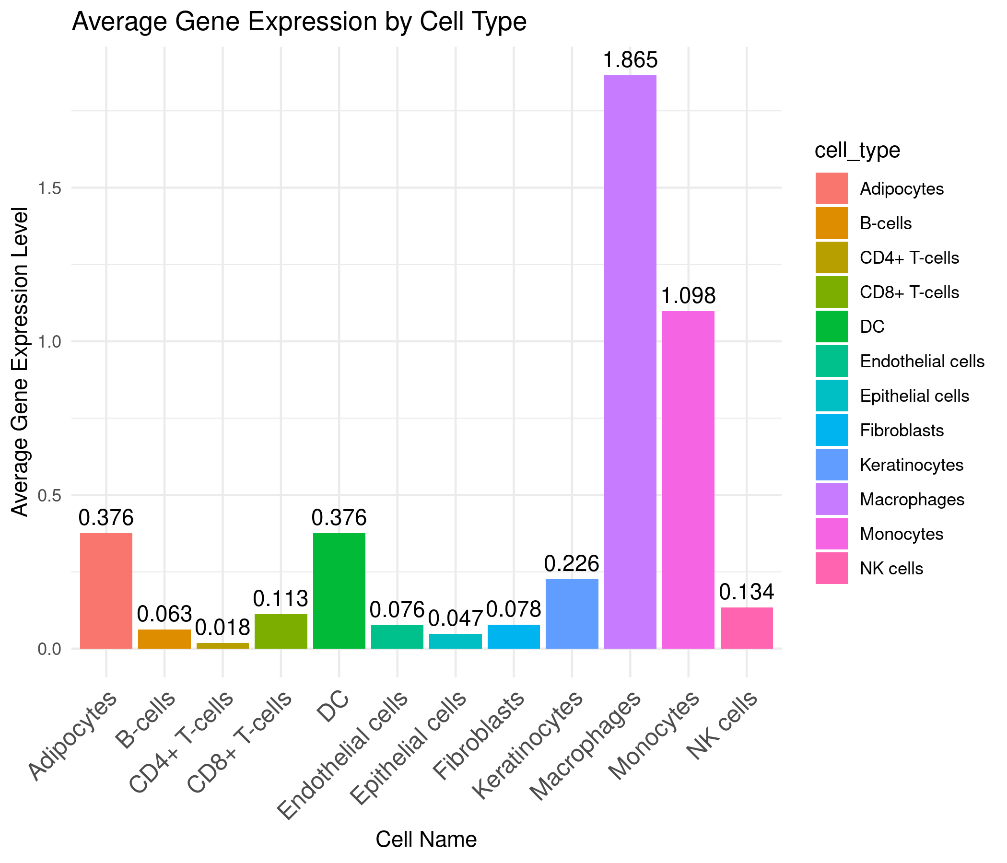


**C**

**
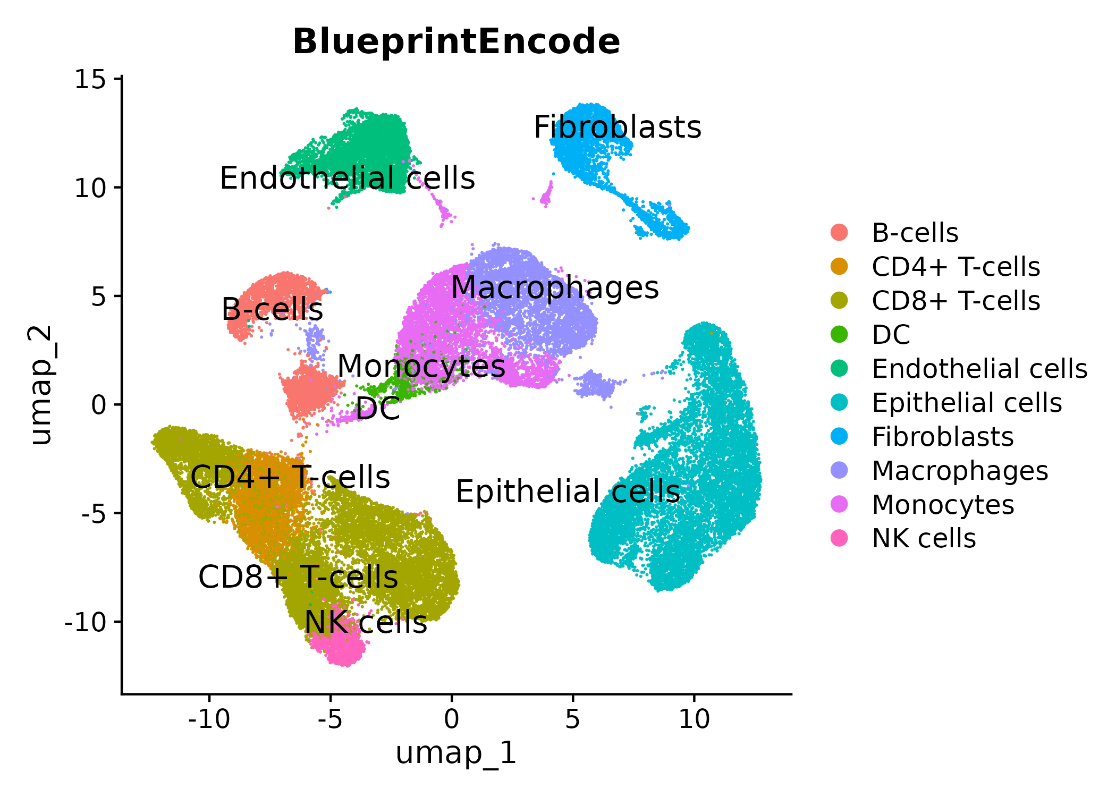
Figure S19.**

**A**

**
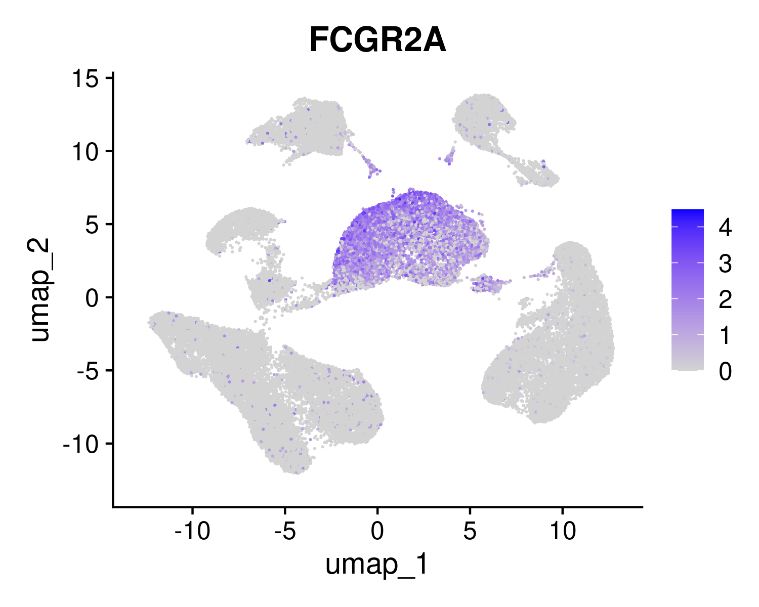
B**

**
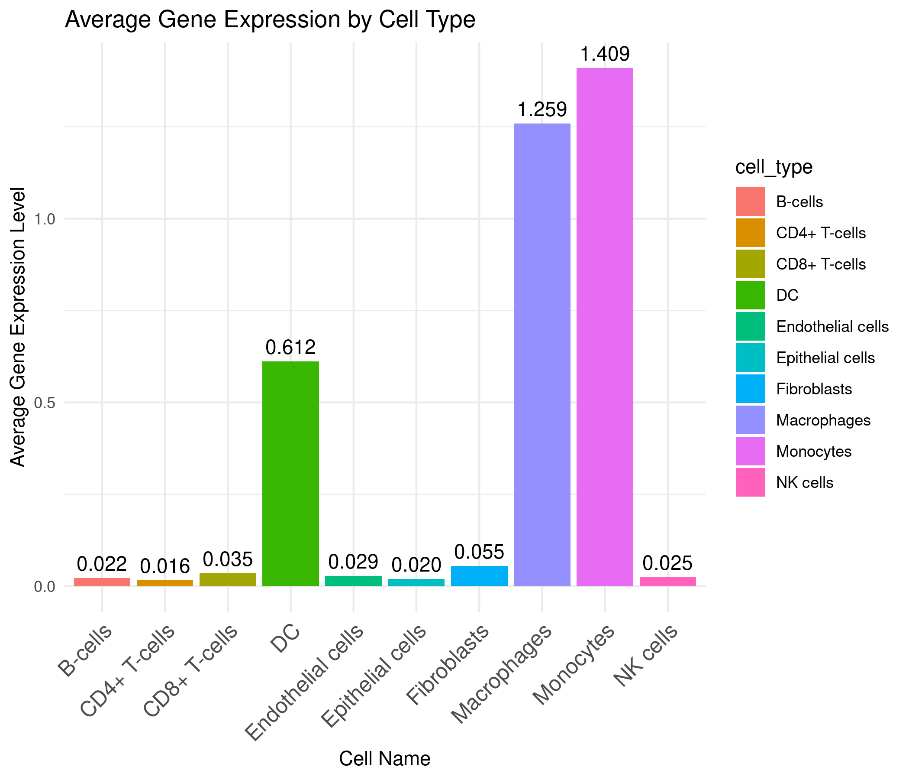
C**

**
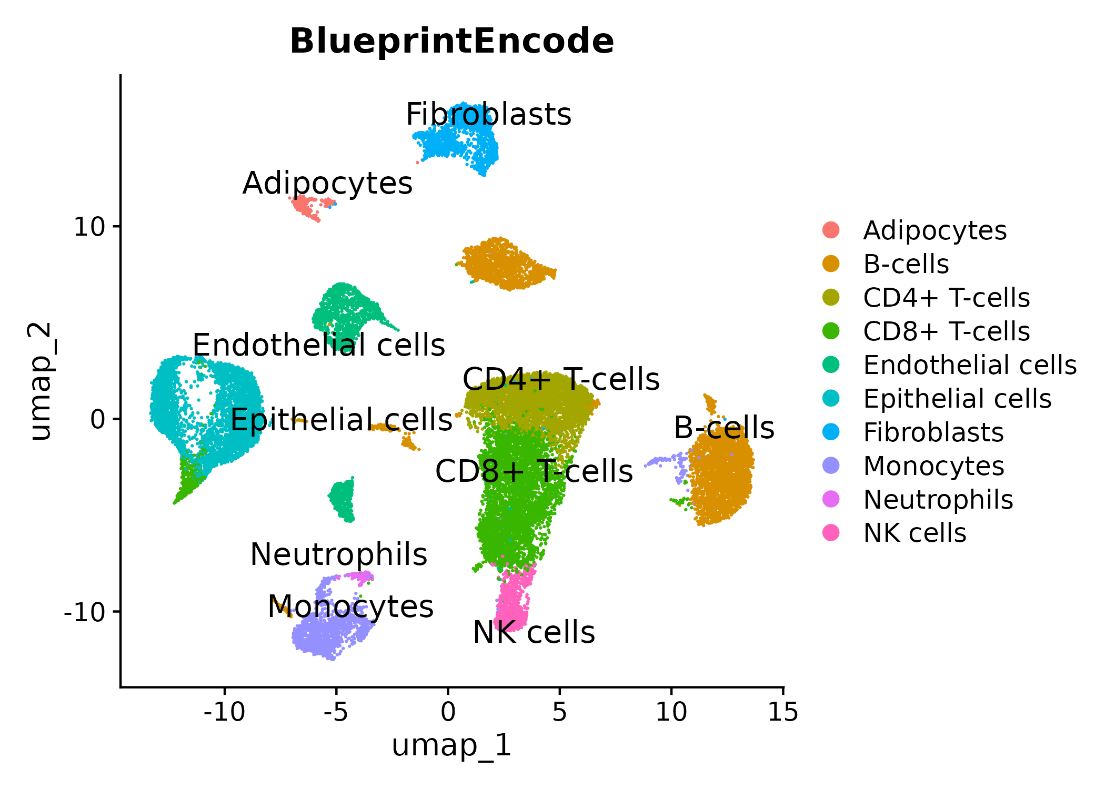
Figure S20.**

**A**

**
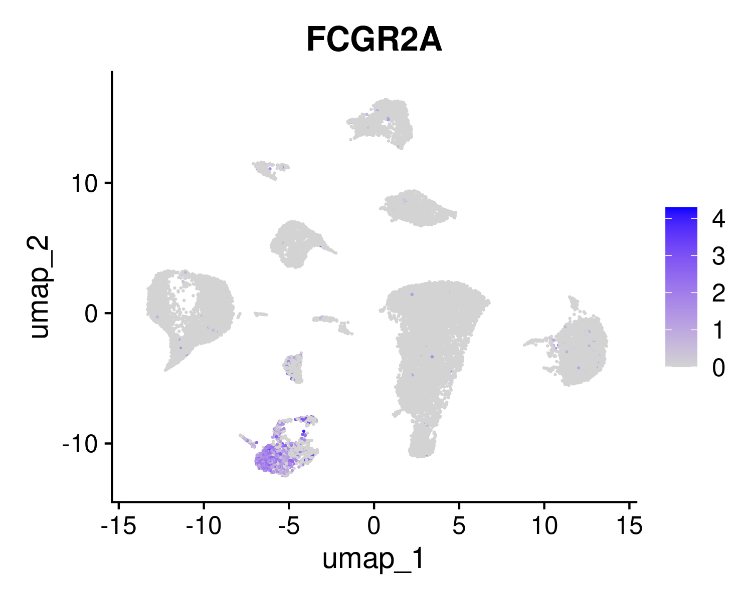
B**

**
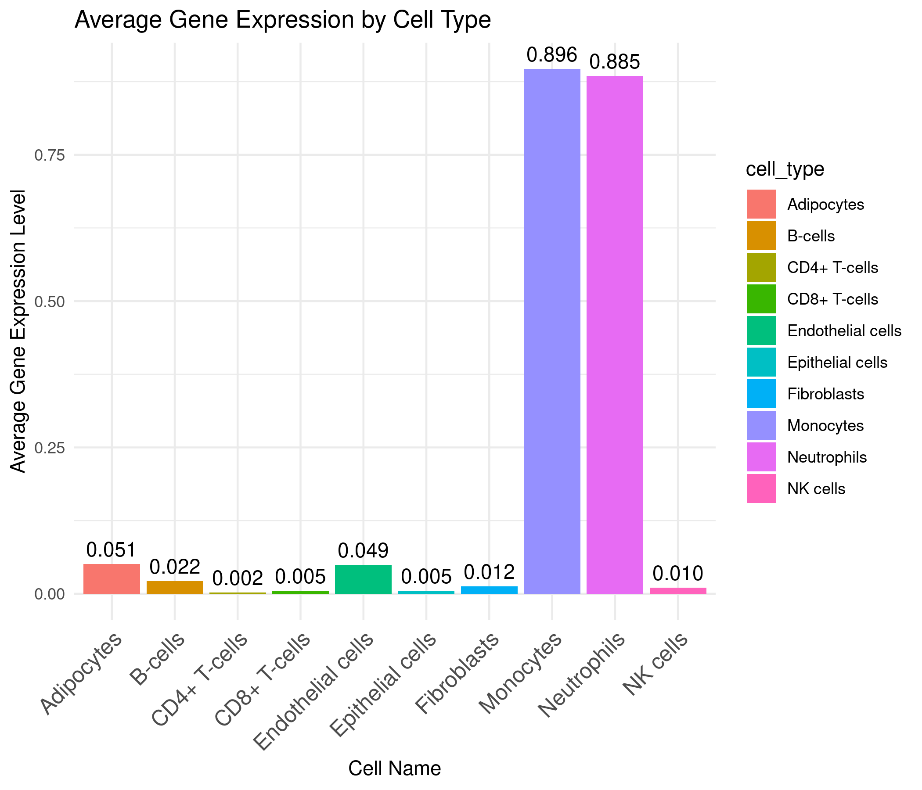
C**

**
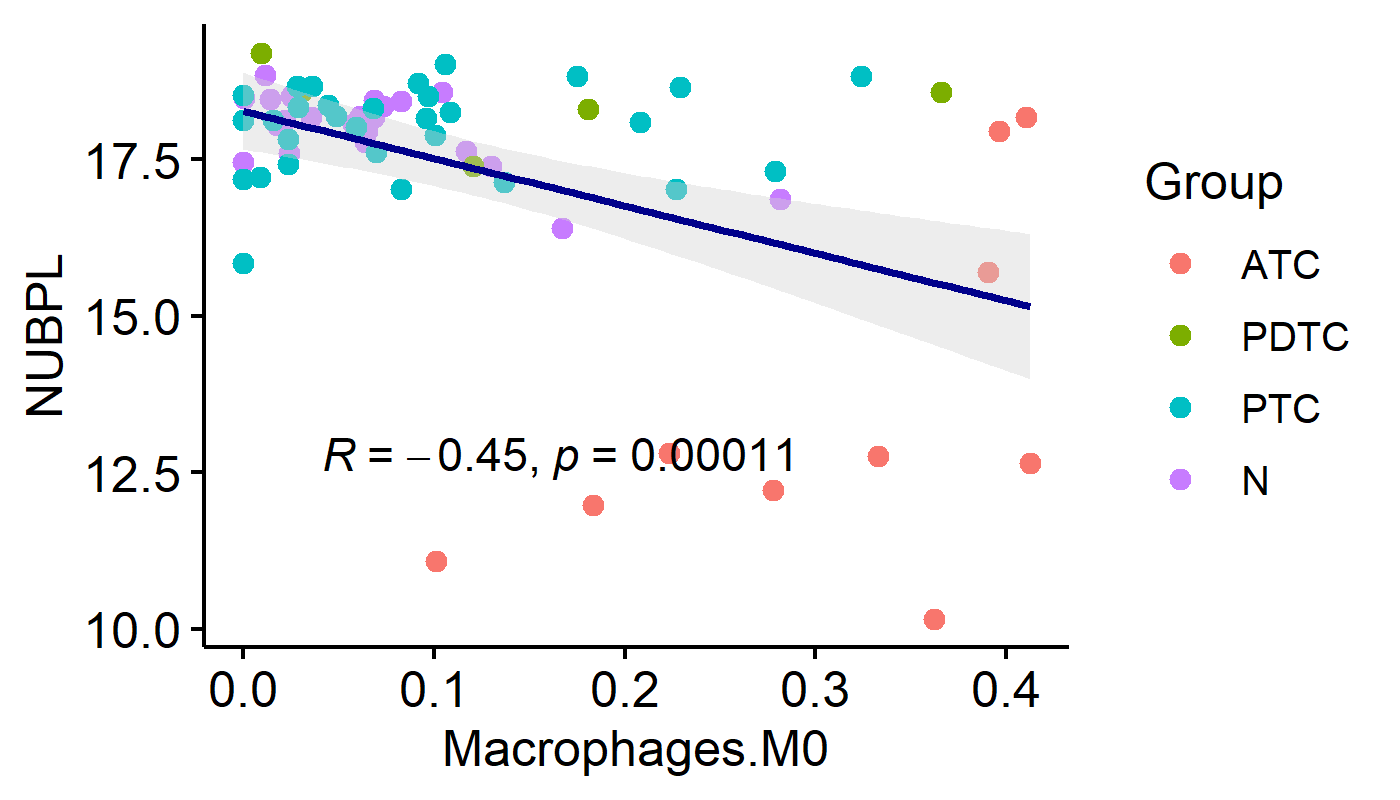

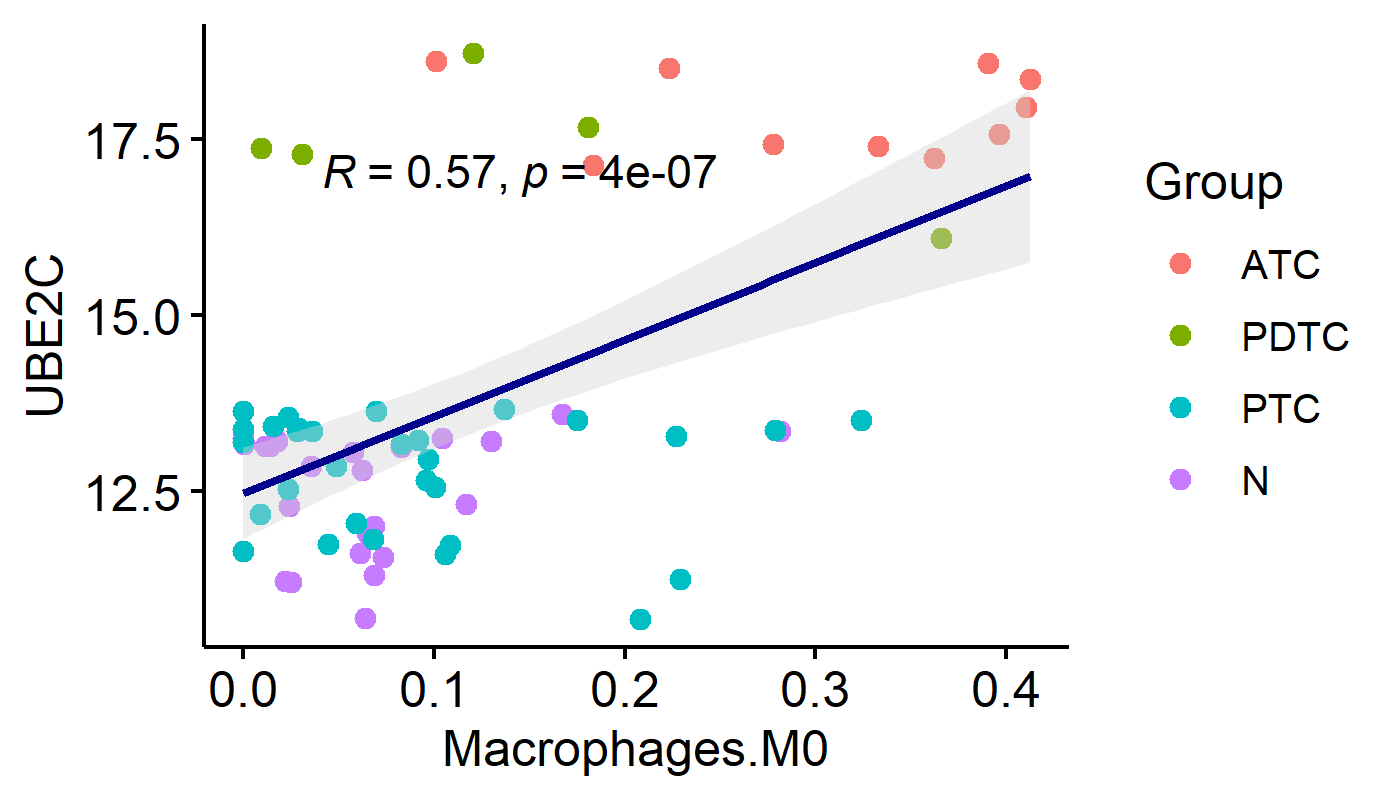
Figure S21.**

**
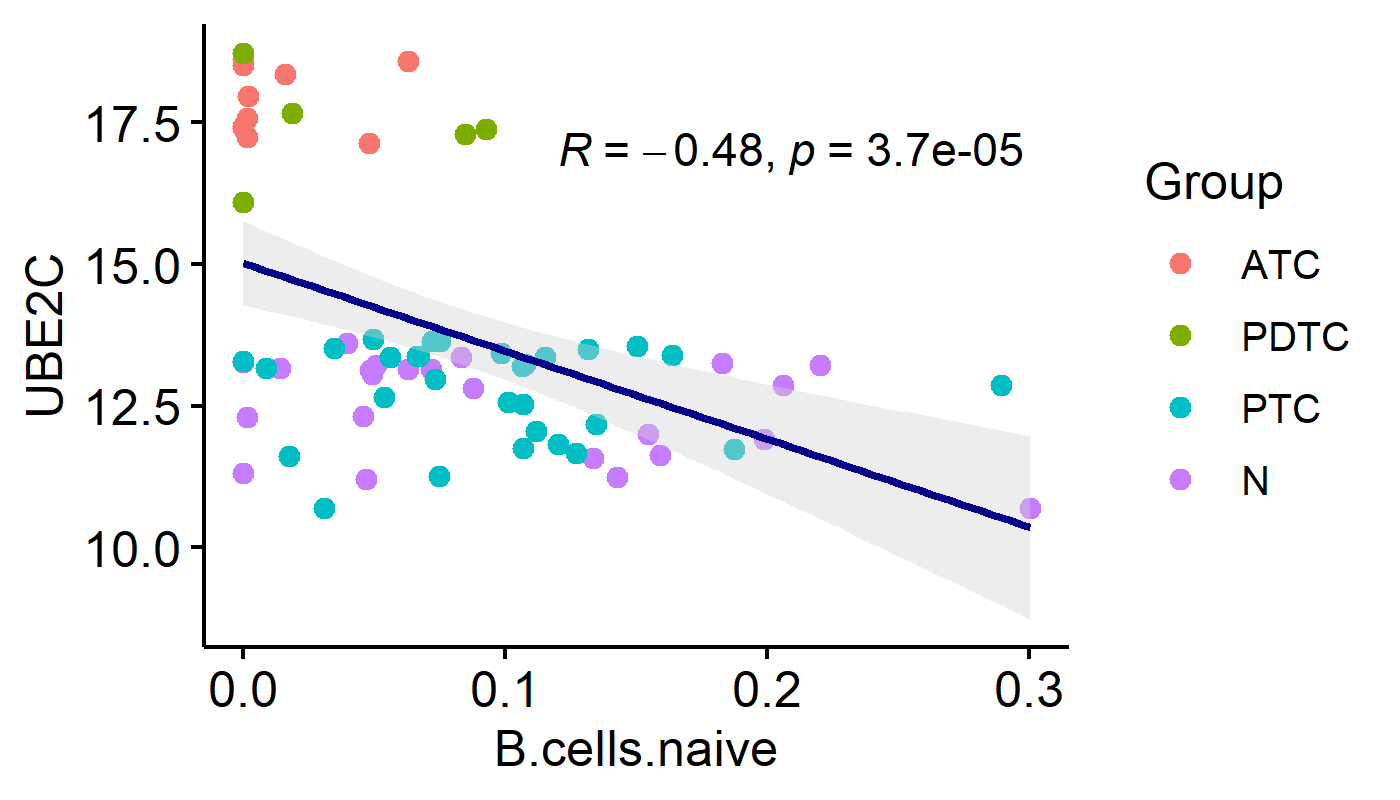
**

**
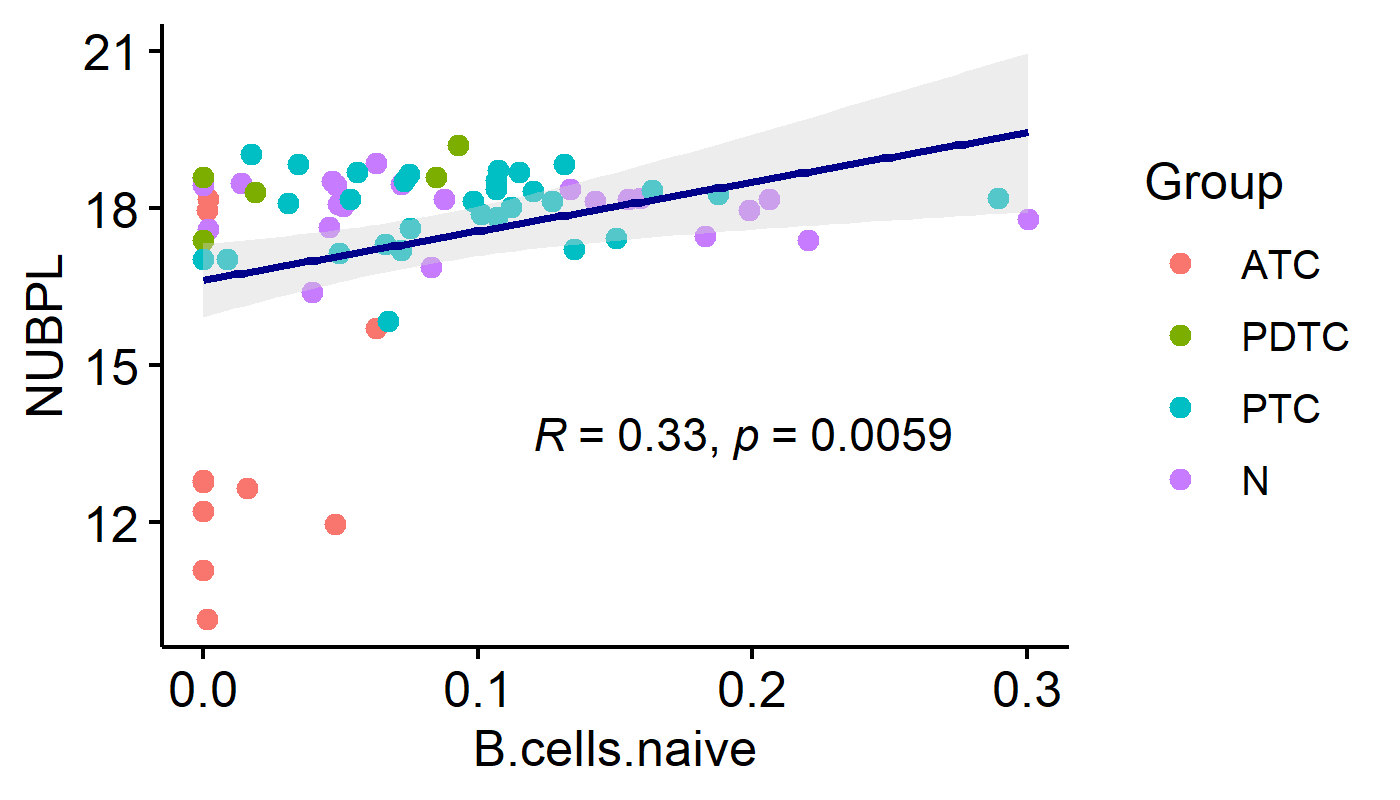
**

**
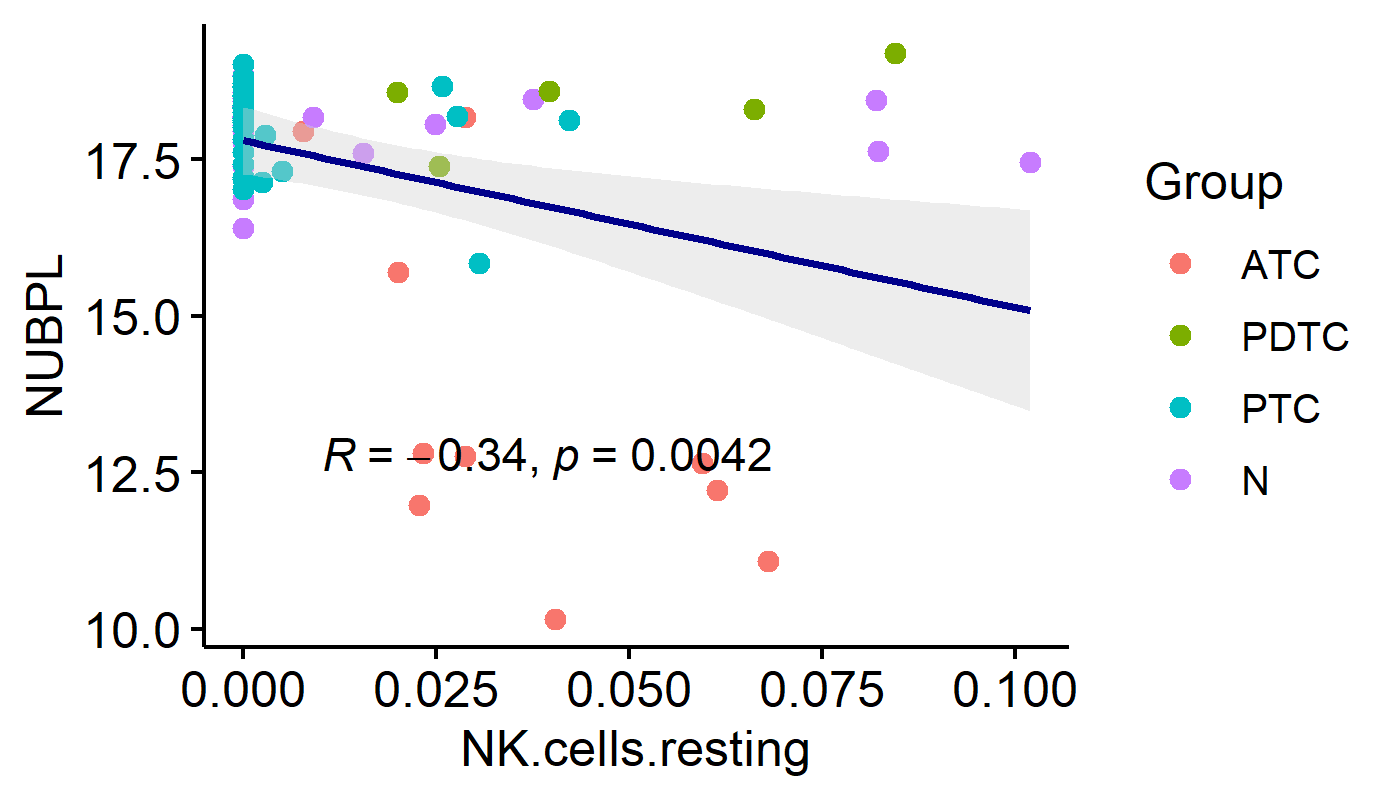

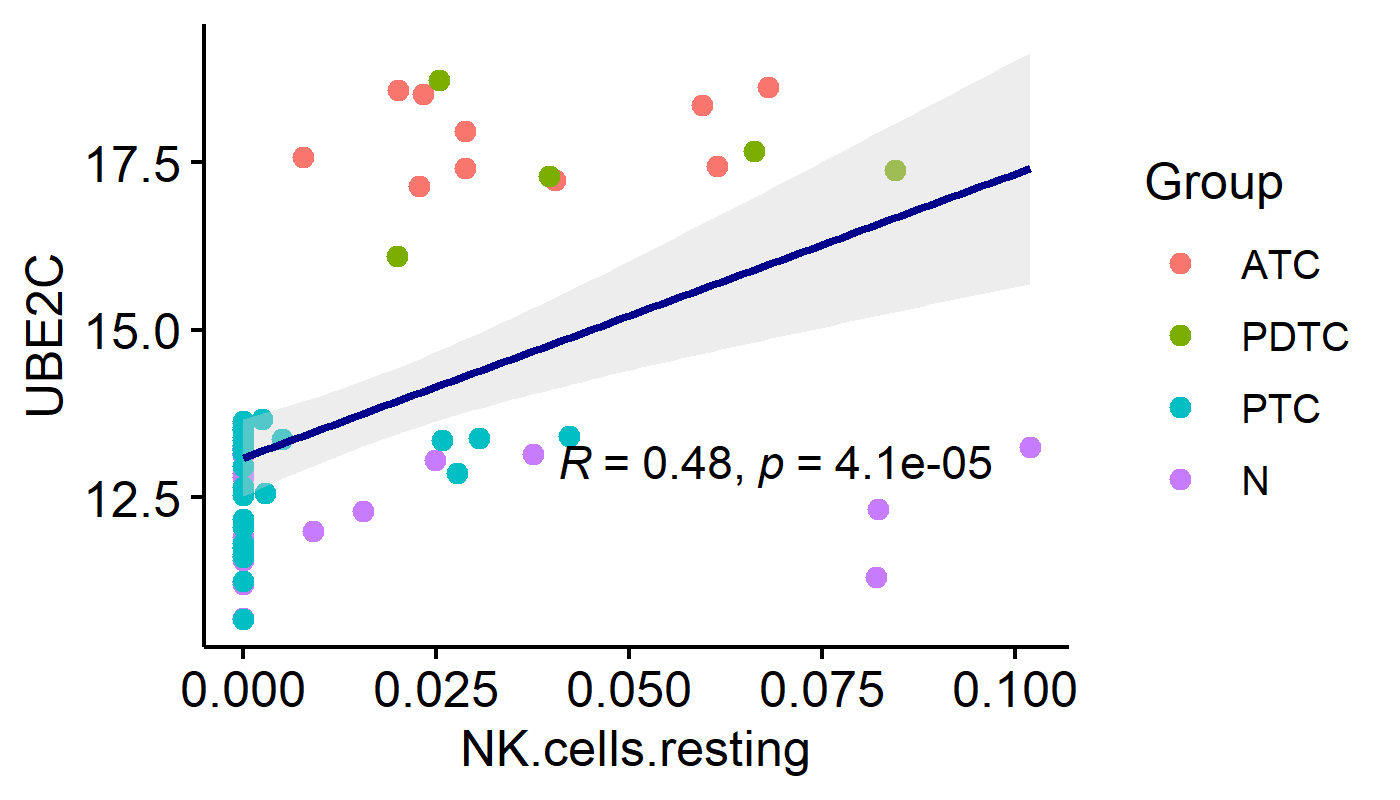
**

**
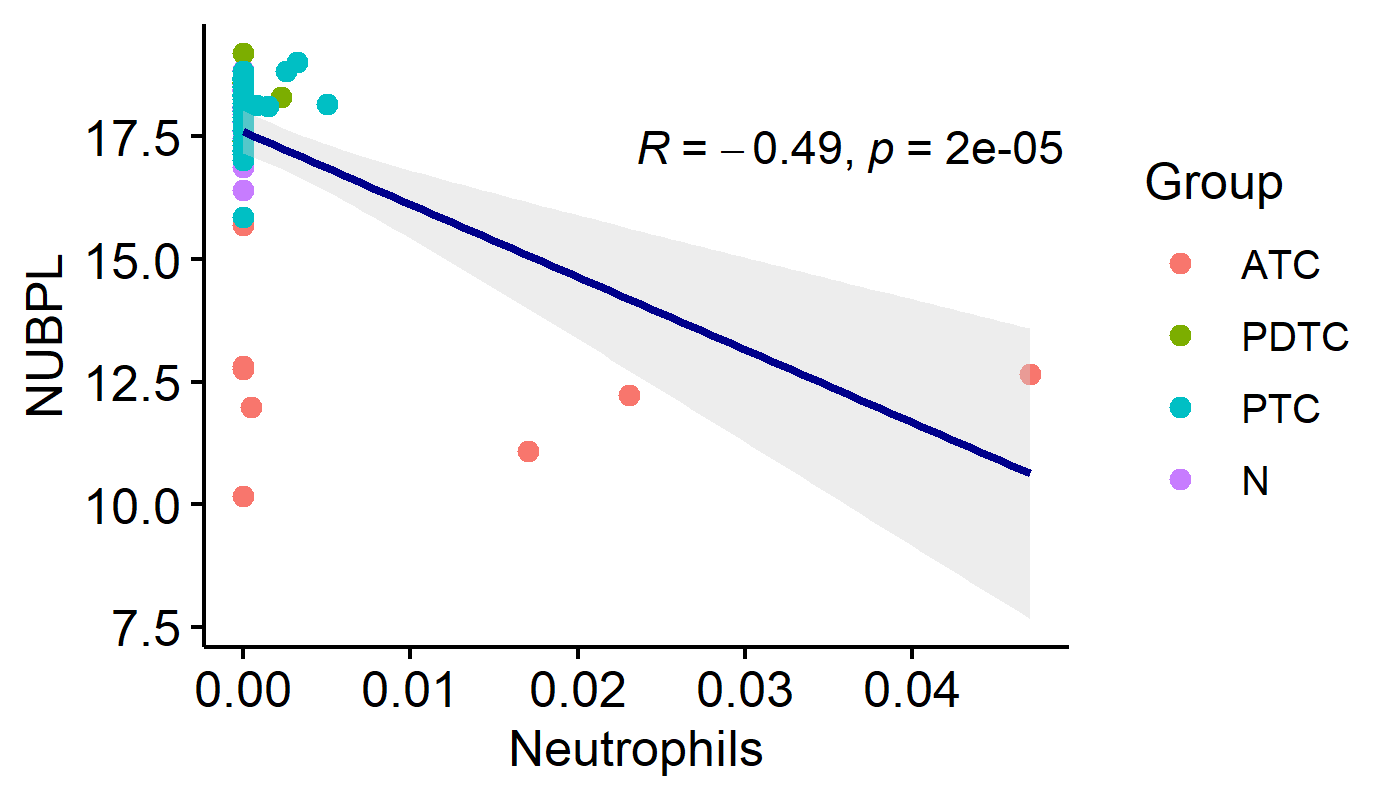

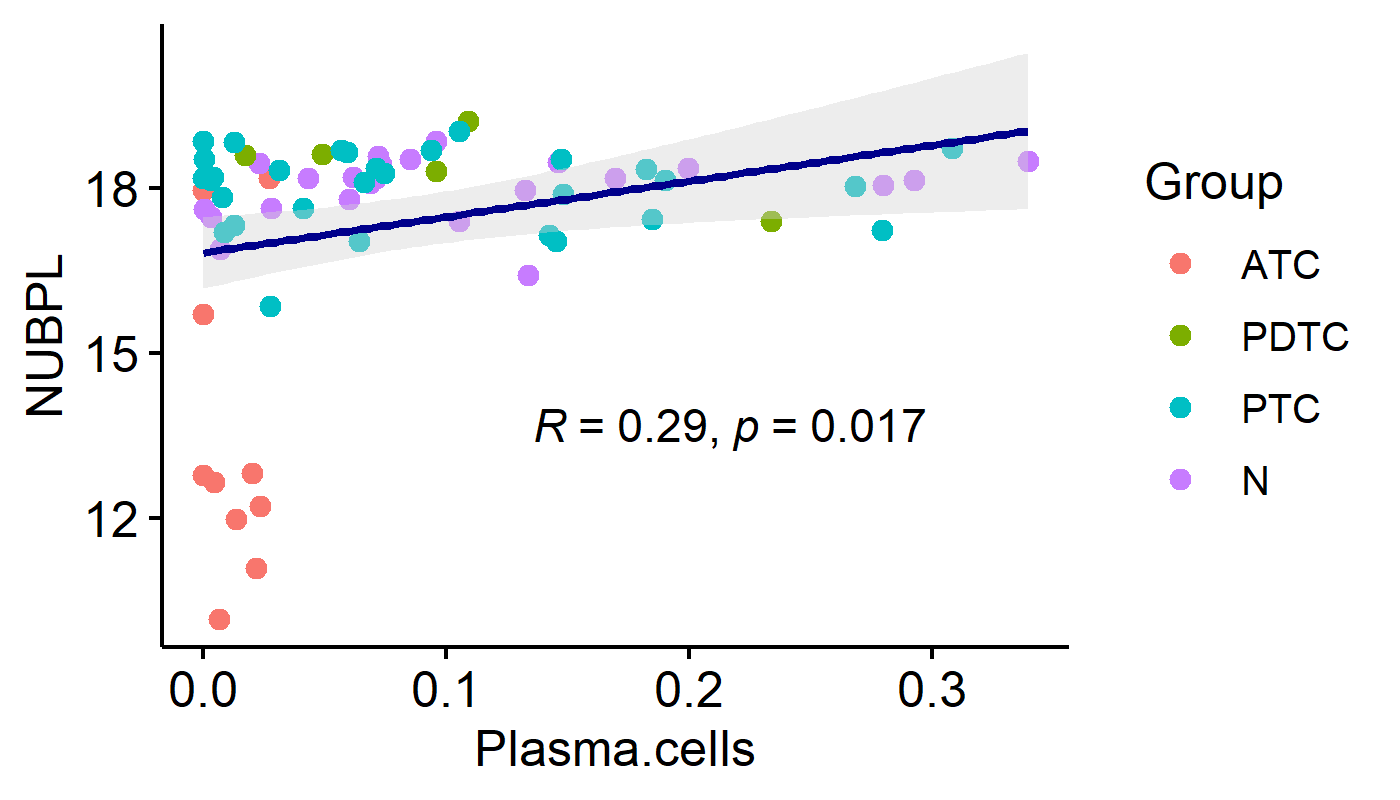

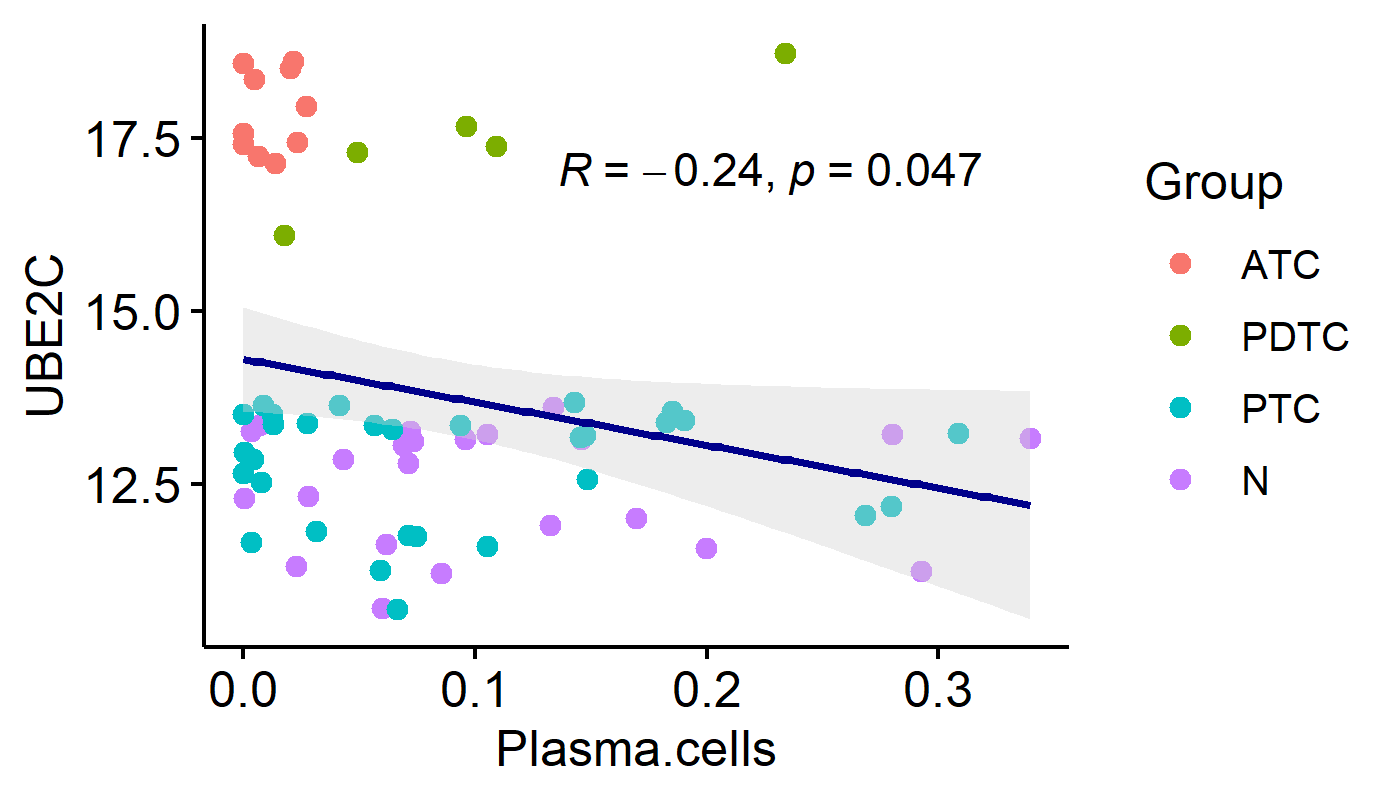

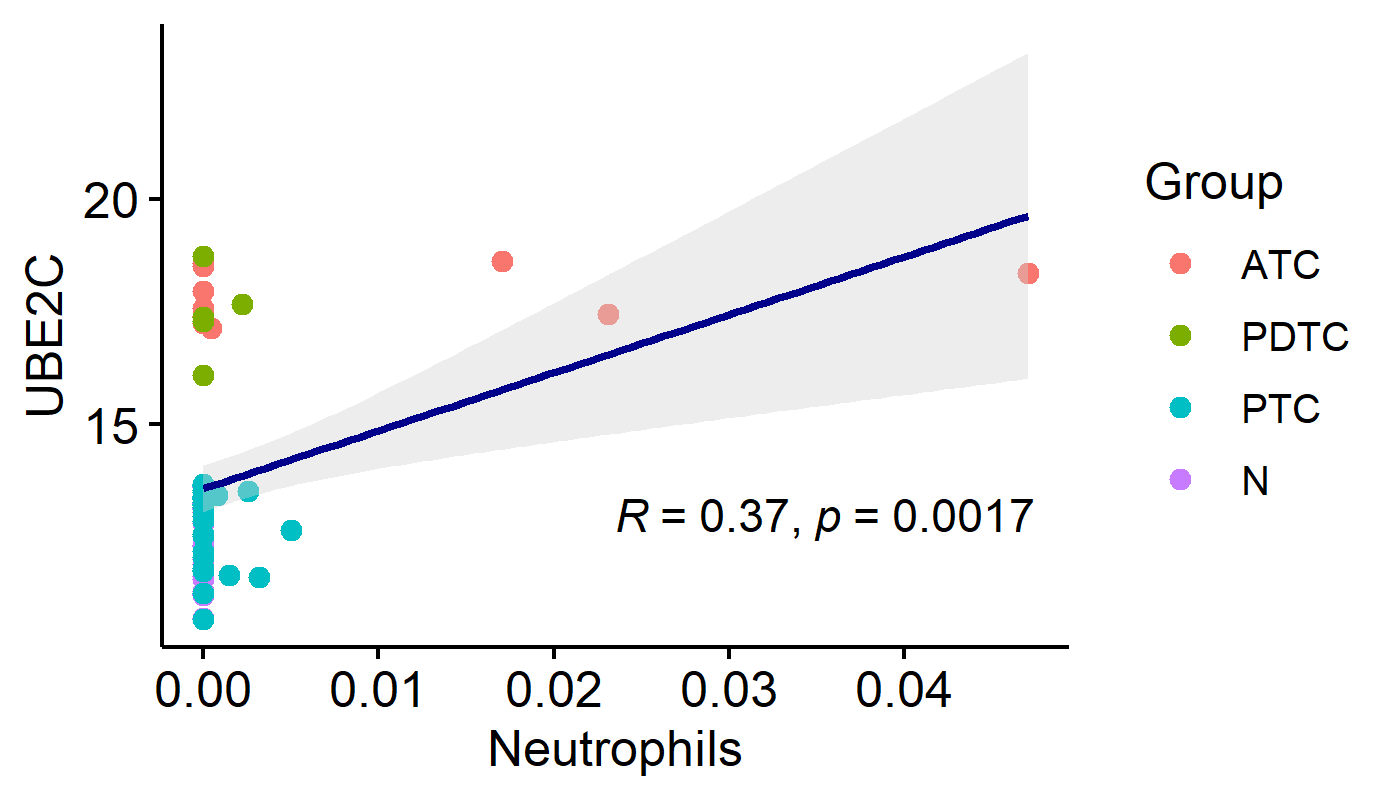
**
